# Rejuvenated Amorphous Alloys: Processing Methods, Microstructures and Mechanical Properties

**DOI:** 10.1002/advs.75703

**Published:** 2026-05-19

**Authors:** Can Yang, Yu Chen, Chunguang Tang, Si Lan, Jun Tan, Heng Ye, Hua Chen, Zhifu Yao, Jürgen Eckert, Dongxian Zhang, Jian‐Zhong Jiang

**Affiliations:** ^1^ Sino‐German College of Intelligent Manufacturing Shenzhen Technology University Shenzhen Guangdong P. R. China; ^2^ Institute For Frontier Materials Deakin University Geelong Victoria Australia; ^3^ Herbert Gleiter Institute of Nanoscience School of Materials Science and Engineering Nanjing University of Science and Technology Nanjing P. R. China; ^4^ College of Materials Science and Engineering Chongqing University Chongqing China; ^5^ Shenzhen Focalon Applied Academy Shenzhen Practical Scientific Research Co., Ltd. Shenzhen Guangdong P. R. China; ^6^ Erich Schmid Institute of Materials Science Austrian Academy of Sciences Leoben Austria; ^7^ Department of Materials Science Montanuniversität Leoben Leoben Austria; ^8^ Key Laboratory of Silicon‐based Materials Key Laboratory of Automotive Glass of Fujian Smart Automotive Glass Engineering Research Center of Fujian The Ministry of Education and School of Materials Science and Engineering Fuyao University of Science and Technology Fuzhou P. R. China

**Keywords:** amorphous alloys, disordered atomic structures, mechanical and thermal processing, mechanical properties, microstructures, rejuvenation

## Abstract

Amorphous alloys are considered promising structural and engineering materials owing to exceptional properties such as high strength/hardness and large elastic limit resulted from disordered atomic structures. However, the intrinsic brittleness and lack of work‐hardening severely restrict their widespread application in structural fields. To address these limitations, rejuvenation strategies have recently been proposed and rapidly developed. These approaches can be broadly categorized into mechanical processing, thermal rejuvenation, and alternative techniques. Rejuvenation not only can be applied to a wide range of alloy compositions including martensitic metallic glass, but also retains alloys disordered amorphous structures. Nevertheless, each route still faces unresolved engineering challenges. While enhanced plasticity and strain‐hardening have been reported in rejuvenated glassy alloys, issues such as premature mechanical failure and the competition and transition between aging and rejuvenation are frequently observed. The transition from brittle to ductile behavior as well as variations in mechanical performance has been closely linked to microstructural features, particularly atomic clusters. Therefore, a thorough understanding of the relationship among rejuvenation processes, microstructural evolution, and mechanical properties is essential. This work aims to provide a comprehensive and critical review of the current progress in this field.

## Graphical abstract

Three rejuvenation methods – mechanical and thermal rejuvenation, and other rejuvenation routes such as ultrasonic processing – can be used to modify structures of glassy alloys, especially atomic clusters, and therefore enhances plasticity and activates work‐hardening behaviors. The expected microstructures at atomic levels can be selected based on both processing parameters and rejuvenation methods.

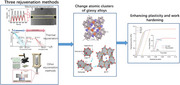

## 1 Introduction of Rejuvenated Amorphous Alloys

Amorphous alloys, also known as metallic glasses (MGs) or bulk metallic glasses (BMGs), are considered as promising structural and engineering materials owing to their outstanding properties such as high strength/hardness and large elastic limit originated from disordered atomic structures [1, 2, 3, 4]. However, such disordered (liquid‐like) atomic configurations also give rise to undesirable features, including catastrophic failure upon elastic deformation and the absence of work‐hardening behavior [3, 4, 5, 6, 7, 8, 9, 10, 11, 12, 13, 14]. For structural applications, two critical challenges remain unresolved: (*i*) achieving a mutualistic relationship between plastic strain and work‐hardening, and (*ii*) scaling up the size of amorphous alloys. These key issues are closely associated with the atomic‐level disordered structures, often described in terms of various atomic clusters [6, 7, 15, 16, 17]. Tailoring the atomic structures has proven an effective method in enhancing mechanical properties of amorphous alloys [2, 3, 12, 18, 19, 20, 21, 22, 23]. For instance, structural modifications at the atomic‐cluster level can promote work‐hardening behavior and mitigate catastrophic failure by suppressing propagation of fatal shear bands (SBs) [1, 2, 3, 6, 7, 10, 18, 19, 20, 21, 22, 23, 24]. Although work‐hardening has been observed in certain monolithic MGs under specific loading conditions [3, 25, 26, 27, 28, 29, 30, 31, 32, 33, 34] or at nanometer scale [15, 35, 36, 37, 38, 39, 40, 41, 42, 43], such restrictions in composition, stress state, and size significantly limit their broader applications in structural fields.

Rejuvenated amorphous alloys, processed through various routes such as mechanical and thermal treatments, exhibit modified regions that consist of both soft and hard domains at the macroscopic scale [5, 7, 9, 11, 18, 21, 44]. At the microscopic or atomic level, these modified structures involve diverse atomic clusters such as atomic arrangements, cluster types, and packing configurations [16, 17], which directly governs deformation behavior of the alloys, including brittleness, ductility, and strain‐hardening [8, 9, 11, 21, 22, 23]. It should be noted, however, that mechanical and thermal processing may lead to either rejuvenation or aging, and a transition between these two states has also been reported [34, 45, 46, 47]. Consequently, rejuvenation, aging, and their transitions have all been closely linked to these underlying atomic structures, particularly the nature of atomic clusters, packing modes, and arrangements [47, 48, 49, 50, 51, 52, 53, 54, 55, 56, 57, 58, 59].

To obtain optimized microstructures and thereby achieve superior properties in processed glassy alloys, it is essential to categorize the reported processing routes. To date, processing approaches designed to activate rejuvenation can generally be classified into three groups: mechanical processing [18, 26, 29, 30, 60, 61, 62, 63, 64, 65, 66], thermal treatments ranging from liquid‐nitrogen temperatures up to the glass transition temperature (*T_g_*) with various cycling numbers [2, 22, 23, 39, 40, 41, 45, 49, 51, 67, 68, 69, 70, 71, 72, 73, 74, 75, 76], and other engineering methods [77, 78, 79, 80, 81, 82, 83, 84, 85, 86, 87, 88, 89, 90]. Within mechanical processing, the strategies can be further divided into cyclic loading and shock compression [26, 29, 30], notch compression [18, 63], and severe plastic deformation (SPD) such as cold rolling and high‐pressure torsion (HPT) [60, 61, 62, 64]. Specifically, in notch compression, notched samples are deformed under constrained compression to different strain levels, and distinct plasticity responses have been observed in notched alloys with a diameter of 1.5 mm [18, 63]. Moreover, thermal rejuvenation methods encompass deep cryogenic cycling/treatment (DCC/DCT) [45, 49, 51, 91], flash annealing [39, 68], and so forth. Notably, during thermal treatments, a mutual interplay between structural aging and rejuvenation has been reported [22, 23, 40, 41]. Finally, other processing routes include ultrasonic vibration (UV) treatments [83, 85, 87, 92], irradiation techniques [78, 81, 82, 88], imprinting [77, 79, 80, 86], etc. Each of these methods must be carefully tuned to manipulate specific atomic clusters and packing configurations within the glassy alloys.

Although some reviews have made some progress regarding rejuvenation methods and deformation, these reviews are not systematical and comprehensive in summarizing and discussing all rejuvenation methods and resulted reports. Furthermore, different from development of glassy composites to enhance plasticity, this review focuses on understanding of modifying atomic clusters and therefore tuning microstructures of glassy alloys and enhancing plasticity. The structure of the present review is as follows. Chapter 2 provides a brief introduction to the characteristics of rejuvenated structures. Chapters 3 to 5 present detailed discussions of three representative processing approaches, namely, the mechanical, thermal, and other related methods. The mechanical behaviors of rejuvenated glassy alloys are critically examined in Chapter 6. Finally, Chapter 7 addresses unresolved issues, proposes potential solutions, and offers a concluding summary.

## 2 Features of Rejuvenated Amorphous Alloys

The features of rejuvenated amorphous alloys can be categorized into macroscopic and microscopic characteristics. From a macroscopic perspective, rejuvenated alloys exhibit heterogeneous structures composed of soft regions and hard domains [5, 7, 8, 9, 12, 18, 19, 21, 22, 35]. At the microscopic or atomic level, their distinctive feature lies in hierarchical or graded atomic clusters with diverse types, arrangements, and packing configurations [47, 48, 49, 50, 51, 52, 53, 54, 55, 56, 93]. Quantification of these features can be fulfilled through different parameters. On the macroscopic scale, relaxation enthalpy, hardness, elastic modulus, and density fluctuation are commonly employed to characterize the heterogeneous soft and hard domains [5, 7, 9, 12, 18, 19, 21]. In contrast, microscopic traits such as atomic clusters can be quantified using descriptors like icosahedral geometries and Voronoi clusters [38, 94, 95]. Accordingly, both macroscopic and microscopic parameters will be briefly discussed in the following sections.

### 2.1 Macroscopic Parameters

**FIGURE 1:**
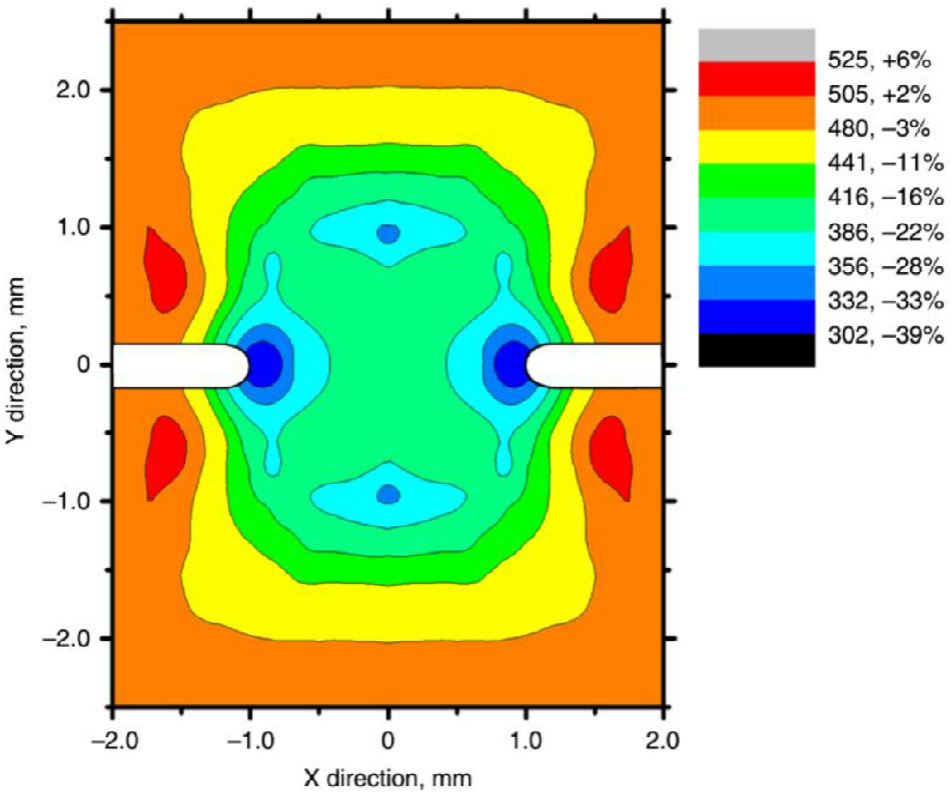
Hardness map of rejuvenated glassy alloys [63]. A gradient hardness can be found in controlled compression regions. The lowest hardness can be observed around the notched points. Reprinted from ref. [63], copyright (2018), with permission from Springer Nature publishing.

Some key parameters (e.g., relaxation enthalpy, hardness/density fluctuations, and elastic modulus) can be used to construct contour maps that distinguish soft and hard regions. Numerous studies have shown that these parameters exhibit regional variations [5, 18, 22, 96, 97, 98, 99, 100, 101, 102], although the trends among them are not always consistent. Some specific observations are given as follows. (i) Relaxation enthalpy reflects heat release or absorption with increasing temperature. A higher positive relaxation enthalpy commonly indicates a greater degree of rejuvenation [45, 51, 99, 100, 101]; (ii) Hardness and density fluctuations are relatively macroscopic indicators of localized structural differences [5, 18, 22, 63, 96, 98, 103]. As shown in Figure 1, areas with lower hardness typically correspond to regions that deform more easily, whereas higher‐hardness regions are generally more resistant to deformation and often located away from the applied stress field; (iii) Elastic modulus and Poisson's ratio are also useful for assessing the deformability of rejuvenated amorphous alloys [97, 98, 102, 104, 105, 106]. For example, a glassy alloy with a large Poisson's ratio (≈0.42) can sustain a compressive strain of up to 20%, with distinct soft and hard regions frequently observed [97]. These deformation behaviors are closely related to the initiation and propagation of SBs, which will be further discussed in Chapter 6.

**FIGURE 2:**
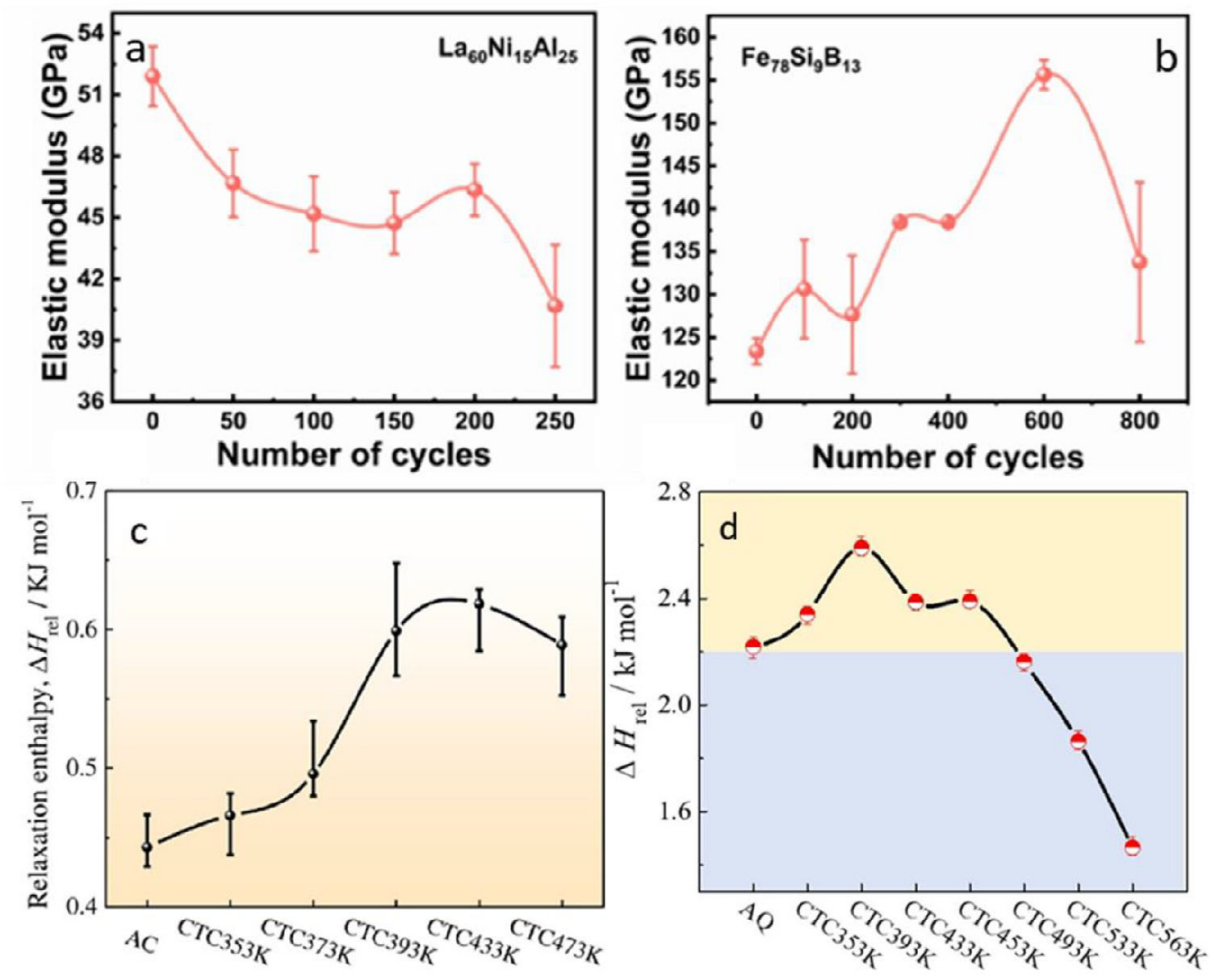
Trend of elastic modulus and relaxation enthalpy of rejuvenated glassy alloys as a function of processing parameters: (a, b) the elastic modulus evolution [45]; (c, d) the relaxation enthalpy [51, 101]. Reprinted from refs. [45, 51, 101], copyright (2022), with permission from Elsevier BV and Chinese Society of Metals Publishing Group.

Here some specific examples illustrating the relationship between rejuvenation behavior of amorphous alloys and their mechanical properties. For glassy alloy of La_60_Ni_15_Al_25_, an increase in the number of processing cycles leads to a continuous decrease in hardness. In contrast, for glassy alloy of Fe_78_Si_9_B_13_, the hardness first decreases and then increases with increasing processing cycles [45]. A similar trend is observed for elastic modulus of La_60_Ni_15_Al_25_, which decreases in parallel with hardness as the number of cycles increases. However, in Fe_78_Si_9_B_13_, the elastic modulus exhibits an opposite trend to hardness, increasing initially and then decreasing with further cycling [45]. For both glassy alloys, the relaxation enthalpy of [(Fe_0.5_Co_0.5_)_0.75_B_0.2_Si_0.05_]_96_Nb_4_ shows a similar trend as that of elastic modulus in Fe_78_Si_9_B_13_ alloy, namely an initial increase followed by a decrease for two distinct initial states [51]. The variations of elastic modulus and relaxation enthalpy with cycling and temperature are summarized in Figure 2.

### 2.2 Microscopic Features

**FIGURE 3:**
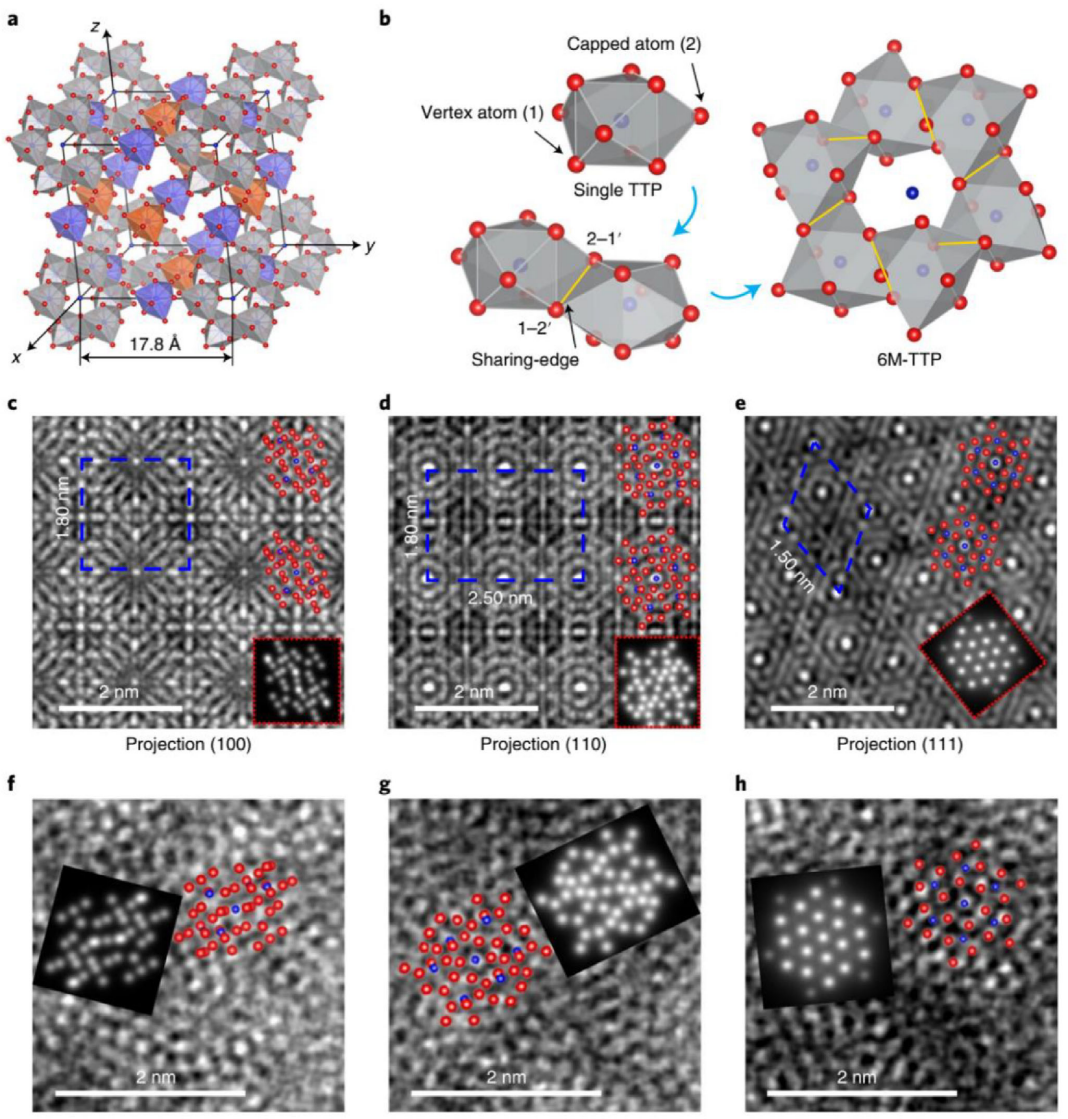
Illustration of atomic packing for cube phase and its backbone building block [16]: (a) unit cell and atomic clusters packing of the cube phase, red ball are for Pd and Ni atoms while blue balls are P atoms; (b) arrangement and packing of atomic clusters showing construction of the 6M‐TTP by the edge‐sharing scheme; (c–e) 2D‐DF HAADF‐STEM images in annealed sample at 653 K; (f‐h) 2D‐DF HAADF‐STEM images for annealed sample at 583 K. Reprinted from ref. [16], copyright (2021), with permission from Springer Nature Publishing Group.

Microscopic features of rejuvenated amorphous alloys are usually associated with fluctuations in atomic clusters [33, 46, 107, 108, 109, 110, 111, 112, 113, 114, 115, 116, 117, 118]. In particular, icosahedral geometries and Voronoi clusters are commonly regarded as representative descriptors of atomic clusters. Reported differences in atomic clusters typically involve variations in types, spatial arrangements, packing density, and atomic linkage length. For instance, the relationship between Voronoi clusters and processing parameters has been studied. The volume fractions of Voronoi clusters such as <0,0,12,0>, <0,2,8,2>, and <0,1,10,2> increase with applied pressure, whereas those like <0,2,8,1> and <0,3,6,3> decrease with increasing pressure [38]. Similarly, the fraction of ordered Voronoi clusters including <0,0,12,0> and <0,2,8,2> are found to increase with a decrease in cooling rate [110]. Figure 3 illustrates representative examples of cluster types, atomic packing motifs, and connection modes. Overall, atomic clusters exhibit variations in terms of type, packing, connectivity, and linkage length, depending on factors such as alloy compositions and processing conditions. These aspects warrant further investigation in future studies. However, icosahedral geometries and Voronoi clusters alone cannot fully capture diversities of atomic cluster configurations. Therefore, additional structural descriptors are required to characterize the complex evolution of atomic clusters under different rejuvenation conditions.

## 3 Mechanical Processing to Induce Rejuvenation

Mechanical processing strategies are widely employed to modify microstructures, for example by increasing heterogeneities or producing graded structures, with an ultimate goal of optimizing mechanical properties [5, 14, 18, 21, 60, 63, 119, 120, 121, 122, 123]. These approaches differ in parameters, and hence the resulting microstructural modifications also vary. Therefore, a comprehensive understanding of different processing methods, the relationships between processing routes, and the resulting microstructural features are essential.

### 3.1 Types of Mechanical Processing

**FIGURE 4:**
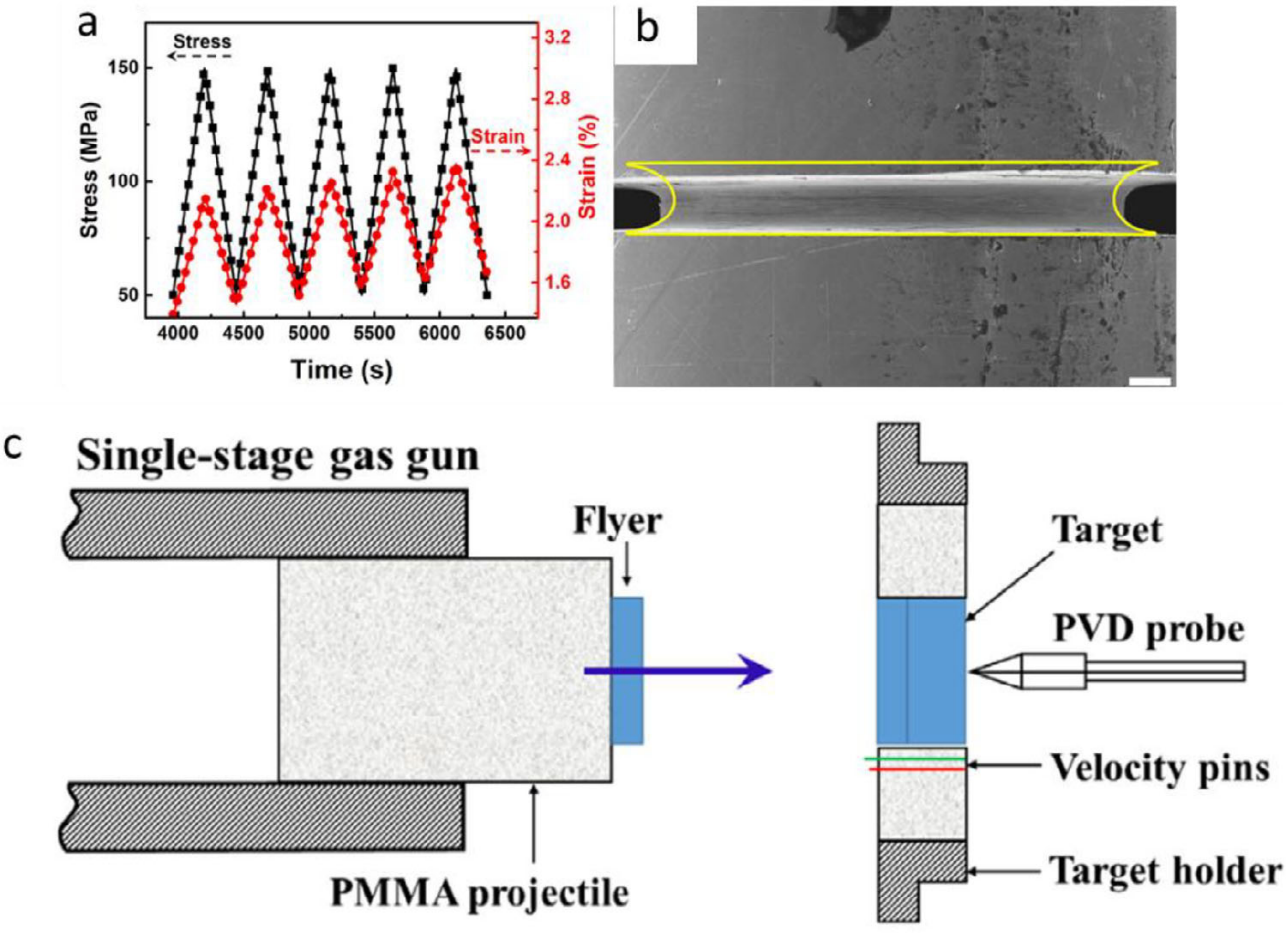
Setups of some processing methods for (a) elastic static compression loading [14], (b) notch compression [63], and (c) shock compression [95]. Reprinted from ref. [14, 63, 95], copyright (2018, 2019, 2022), with permission from Elsevier Ltd, Springer Nature, and American Association for the Advancement of Science Publishing Group.

To modify microstructures and thereby enhance plasticity as well as activate work‐hardening, several mechanical rejuvenation processing methods have been proposed [13, 14, 18, 25, 28, 60, 61, 62, 63, 64, 121, 122, 123, 124, 125, 126, 127, 128, 129, 130, 131, 132, 133, 134]. The methods include the SPD [25, 121, 122], notch compression (a combination of notch and compressive loading) [13, 28, 63, 123, 124, 125, 126, 127, 128, 129, 130, 131], elastic static compression with different loadings up to yielding stress [14], HPT [60, 61, 62, 64, 119, 120], and elastic static loading studied via molecular dynamics (MD) simulations [13]. Representative setups of some processing methods are shown in Figure 4. Each method involves distinct processing parameters (detailed in Section 3.2.2), which in turn leads to different microstructural modifications.

#### 3.1.1 Mechanical Compression With Various Conditions

##### 3.1.1.1 Experimental Designs

The cycling compression (or mechanical cycling compression) refers to dynamic loading–unloading cycles in a linear elastic regime, as illustrated in Figure 4a. Effects of cycling compression on degrees of rejuvenation can be modulated by stress amplitude, stress rate, and mean stress [14]. An increase in any of these parameters can intensify the rejuvenation effect. The notched compression process is carried out as shown in Figure 4b [63]. A selected glassy rod is circumferentially notched by gentle grinding in a custom‐made machine, followed by careful surface polishing and ultrasonic cleaning. The laterally elasto‐static loading (ESL) is defined as compression applied under a stress level lower than yielding stress of glassy alloys [123]. According to recent studies [63, 123, 128, 132], the applied stress typically corresponds to 40, 60, 70, 80, or 90% of the yielding stress. The shock compression [95], shown in Figure 4c, is performed using a setup that includes a light‐gas gun, flyer, target materials, contact plates, and a projectile. The light‐gas gun launches the projectile, causing the flyer to impact the target at a controlled initial velocity. A range of impact velocities (e.g., 270, 360, 480, and 520 m/s) is applied to tailor degrees of rejuvenation in glassy alloys. Finally, pre‐compression shares similarities with cycling compression. However, in this case the applied stress may exceed the yielding stress, after which the compressed alloys are unloaded.

##### 3.1.1.2 Experimental Operations

**FIGURE 5:**
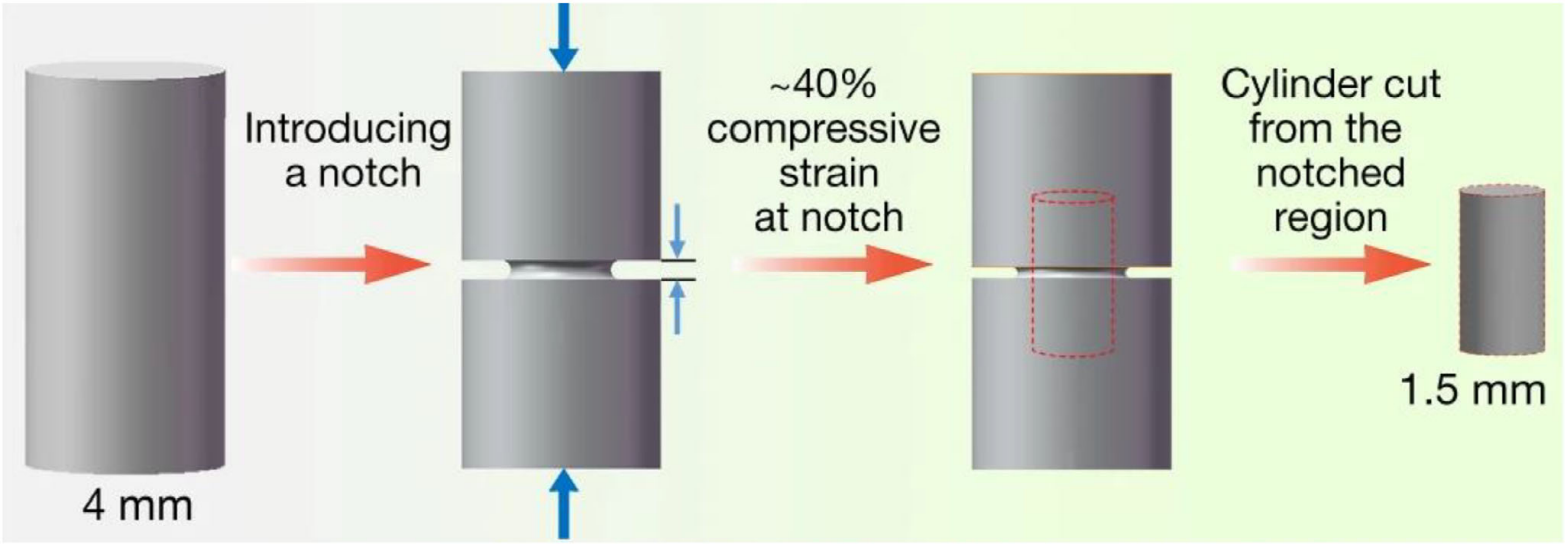
Constrained loading compression [18, 63]. A 4 mm‐diameter rod of Zr_64.13_Cu_15.75_Ni_10.12_Al_10_ BMG is machined to have a circumferential notch. Uniaxial compression induces plastic deformation, reducing the notch width by 40%. This processing produces a highly rejuvenated region within the notched rod, from which a glass cylinder with a diameter of 1.5 mm and a length of 3 mm is machined. Reprinted from refs. [18, 63], copyright (2018, 2020), with permission from Springer Nature Publishing Group.

Experimental operations or procedures may vary depending on processing methods and alloy compositions. For instance, glassy Zr_64.13_Cu_15.75_Ni_10.12_Al_10_ samples were naturally aged for 6, 6.5, and 8 years at room temperature, and subsequently subjected to lateral ESL compression at 60% of the yielding stress for 1 h [129]. The rejuvenated glassy alloy aged for 6 years exhibited a pronounced *β*‐relaxation and the highest plastic strain. Representative notched glassy alloys underwent processing and treatment are shown in Figure 5. To achieve different levels of plastic strain (e.g., 20% and 40%), various yielding stresses were applied to amorphous alloys [63]. In another procedure, glassy alloys were partially annealed and then subjected to lateral ESL compression. Specifically, annealed samples were compressed at 60% of the yielding stress for different durations (e.g., 0.5, 1, 2.5, 4, 7, and 10 h) at room temperature [28].

##### 3.1.1.3 Experimental Results

The slight loading‐unloading compression within an elastic limit (yielding stress) is an important approach to increase the relaxation enthalpy of glassy alloys [13, 28, 57, 123, 124, 125, 126, 127, 128, 129, 130, 131, 135]. For instance, in notched compression [18, 63], an increase in deformation in notched parts under constrained compression has been shown to enhance the relaxation enthalpy [18], accompanied by a corresponding increase in plastic strain. According to Refs. [37, 63, 136], processed glassy alloys exhibit decreased hardness and increased relaxation enthalpy as the deformation in notched parts increases. Similarly, the relaxation enthalpy rises with the as‐cast glassy alloys. For example, they show a relaxation enthalpy of 0.49 kJ/mol, while that of the un‐notched sample strained to 40% increases to 0.65 kJ/mol, and that of the notched sample strained to 40% further increases to 1.13 kJ/mol. In the case of shock compression [95], the degree of rejuvenation can be effectively controlled by appropriately selecting the shock stress amplitude. The authors [95] also argued that the observed rejuvenation jump primarily results from anelastic deformation, while adiabatic heating plays only a minor role. For mechanical cycling [14], the process can be classified into three modes defined by two fixed parameters and one variable parameter.

For laterally applied ELS compression [28, 64, 127, 128, 130, 132, 137, 138], the relationship between relaxation enthalpy and degree of rejuvenation have been investigated in several studies. For the Zr_35_Ti_30_Be_27.5_Cu_7.5_ glassy alloy, two levels of applied yielding stress (50 and 90%) were examined [128]. The processed alloys exhibited different relaxation enthalpies, namely, 2.967, 1.211, and 5.299 J/g after compression at 50%, 70%, and 90% of the yield stress, respectively. It's found that compared with that of as‐cast state (3.935 J/g), alloys treated at 50 and 70% yield stress showed reduced relaxation enthalpies, whereas the alloy compressed at 90% yield stress displayed an increased value. Similarly, an elasto‐static compression at stresses corresponding to 40%, 70%, 80%, and 90% of the yield stress was applied to rejuvenate Zr_55_Cu_30_Al_10_Ni_5_ [130]. A maximum relaxation enthalpy of 13.2 J/g was obtained at 90% yield stress, accompanied by a reduction in hardness, a steady increase in plastic strain, and a decrease in Young's modulus.

Moreover, the transition between rejuvenation and aging can also be tuned. For example, mechanical relaxation‐to‐rejuvenation of a Zr_35_Ti_30_Be_27.5_Cu_7.5_ glassy alloy has been reported under an elasto‐static compression at room temperature. The competitive interplay between activation enthalpy and activation entropy during stress‐driven and temperature‐driven processing of glassy alloys is believed to be the main factor governing this transition. In particular, lateral ESL compression can induce either relaxation or rejuvenation, depending on the processing time [123, 128]. In these experiments, ESL compression was applied at 90% of the yield stress to glassy samples with five distinct initial states in order to identify transition states such as relaxation and rejuvenation. The results indicated that the final energy states of the alloys are strongly determined by their initial configurations. It should be emphasized that the degree of deformation plays a crucial role in such a way that the elastic (small strain) deformation generally leads to aging, whereas non‐elastic (large strain or plastic) deformation can result in either aging or rejuvenation [65]. Specifically, under small‐strain conditions, aging dominates while rejuvenation is negligible, whereas under large deformation, both aging and deformation‐induced rejuvenation can occur simultaneously.

#### 3.1.2 Spd

##### 3.1.2.1 Experimental Designs

**FIGURE 6:**
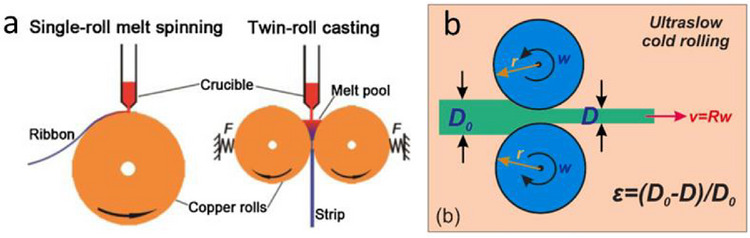
Setups of some SPD processing methods such as (a) twin‐roll and single‐roll [121]; (b) ultraslow cold rolling [25]. Reprinted from refs. [25, 121], copyright (2020, 2024), with permission from Elsevier and Chinese Society of Metals Publishing Group.

The SPD process includes several routes such as cold rolling [25, 121], HPT [26, 29, 30, 33, 60, 61, 62, 64, 119, 120], and twin‐roll casting [121]. These processing methods can significantly modify morphology of the original glassy alloys. Setups of twin‐roll, single‐roll, and ultraslow cold rolling are illustrated in Figure 6. The glassy melt is spouted onto a single‐roll or twin‐roll copper caster [121]. For ultraslow cold rolling [25], the strain rate is as low as 10^−6^ s^−1^, whereas conventional cold rolling typically operates at a much higher rate. As another representative SPD technique, the HPT involves intensive deformation that not only introduces excess free volume but also promotes extensive atomic rearrangements [30]. The rotation speed during HPT can be adjusted according to processing requirements. Overall, SPD provides some efficient means to alter microstructures. However, its capability to precisely control the targeted formation of specific microstructures remains limited.

##### 3.1.2.2 Experimental Operations

In ref. [121], single‐ and twin‐roll casting machines were employed to fabricate glassy ribbons with thicknesses of 40, 110, 320, and 490 µm. The HPT processing has been applied to a variety of glassy compositions [26, 29, 60, 61, 62, 119, 120]. For example, glassy rods of Zr_65_Cu_18_Ni_7_Al_10_ with a diameter of 10 mm were processed by HPT at room temperature under a compressive pressure of 5 GPa [60]. The deformation was conducted at a rotation speed of 1 rpm with different numbers of rotations (*N*). Similarly, the glassy alloy Zr_52.5_Cu_17.9_Ni_14.6_Al_10_Ti_5_ was subjected to HPT at a compressive pressure of 6 GPa and room temperature [61]. Processing was performed at 0.6 rpm with rotation numbers ranging from 0 to 40. In addition, a glassy rod of Cu_45_Zr_45_Al_5_Ag_5_ was deformed under a compressive pressure of 4 GPa at room temperature [62], using a rotation speed of 0.2 rpm up to 80 turns. Another glassy alloy, Zr_50.7_Cu_28_Ni_9_Al_12.3_, prepared in the form of a disc (10 mm in diameter and 0.8 mm in thickness), was processed by HPT at a compressive pressure of 6 GPa at room temperature, with deformation carried out for 1 and 2 revolutions. A glassy rod (10 mm in diameter) of Zr_50_Cu_40_Al_10_ was also subjected to HPT processing [26] at 5 GPa and room temperature, with rotation numbers ranging from 1 to 50. Furthermore, the Cu_45_Zr_45_Al_5_Ag_5_ alloy was deformed by HPT under compressive pressures of 4 or 8 GPa at either room temperature or liquid nitrogen temperature, up to 80 turns [29]. In the case of ultraslow cold rolling, glassy alloys with thicknesses of ∼14.25 and ∼12 µm were processed at rolling speeds of approximately 8.25 × 10^−8^ and 5.98 × 10^−8^ s^−1^, respectively [25].

##### 3.1.2.3 Experimental Results

A series of SPD methods have been applied to modify the microstructures of both crystalline and amorphous alloys. It has been reported that SPD processing can effectively alter volume fraction of shear transformation zones (STZs) [61], thereby influencing formation of SBs [33]. In addition, a transition between structural aging and rejuvenation has been observed in amorphous alloys subjected to HPT [26]. The rejuvenation process induced by HPT can be divided into four stages, that's, an initial rapid rejuvenation, a quiescent period, a subsequent rejuvenation stage, and a final saturation stage [33]. For instance, when the number of revolutions is between 5 and 50, rejuvenation behavior is not apparent. However, once the revolutions increase to about 100, a significant rejuvenation characterized by an increased structural enthalpy can be detected. The relaxation enthalpy can also be tuned by SPD. These calculated relaxation enthalpies for two ribbons and two strips are 1253 ± 30, 976 ± 12, 1050 ± 24, and 917 ± 12 J/mol for alloys with thicknesses of 40, 110, 320, and 490 µm, respectively [121]. These results indicate that twin‐roll casting increases the relaxation enthalpy, as the enhancement associated with larger thicknesses outweighs the reduction in enthalpy. The increase in relaxation enthalpy of amorphous alloys produced by twin‐roll casting may be attributed to presence of additional 2‐ and 4‐atom connections. Moreover, another study demonstrated that the relaxation enthalpy gradually increases with the volume fraction of severely deformed regions [29].

#### 3.1.3 Bending/High‐Pressure Rejuvenation

##### 3.1.3.1 Experimental Design and Operations

**FIGURE 7:**
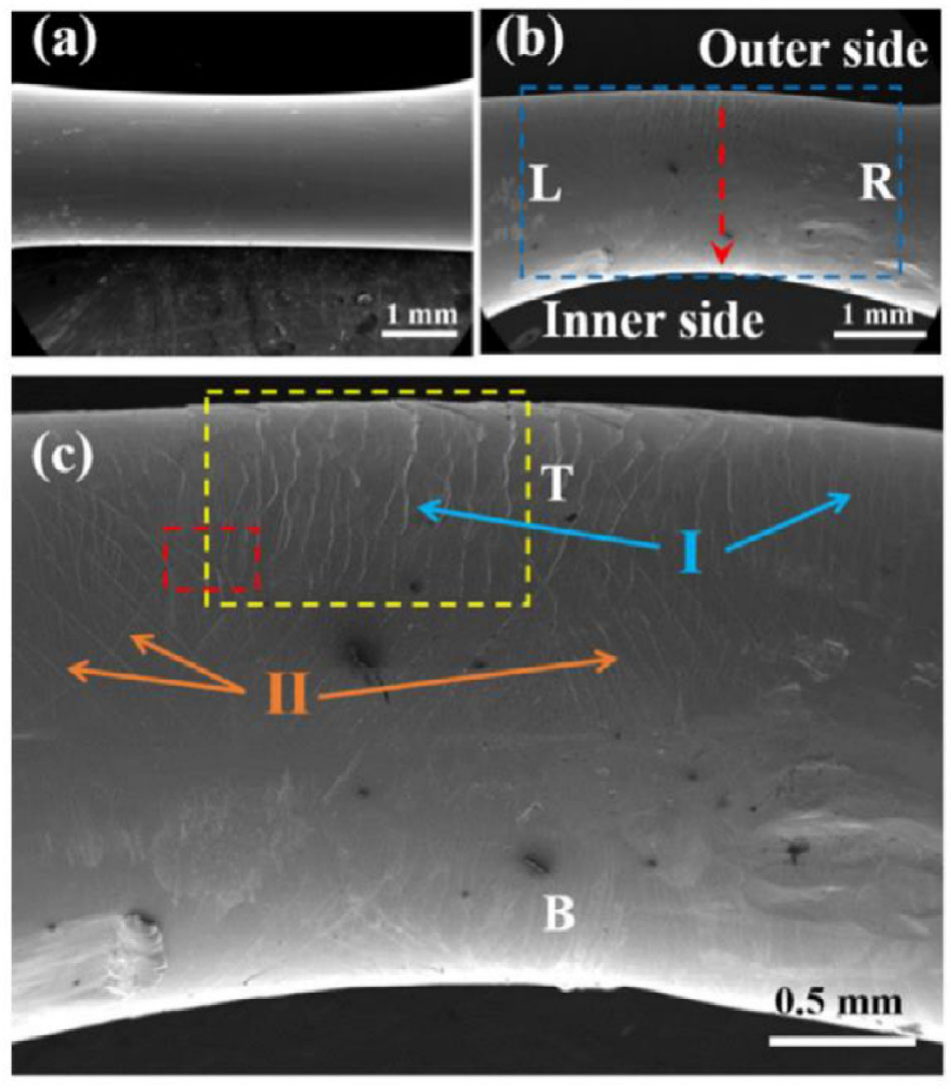
Description of bending glassy alloys in terms of inner and outer sides [94]. For both sides, different stresses (compressive and tensile loadings) were applied on glassy alloys. (a) as‐cast samples; (b) bent samples; (c) zoom‐in view of (b). Reprinted from ref. [94], copyright (2020), with permission from Elsevier Ltd Publishing Group.

**FIGURE 8:**
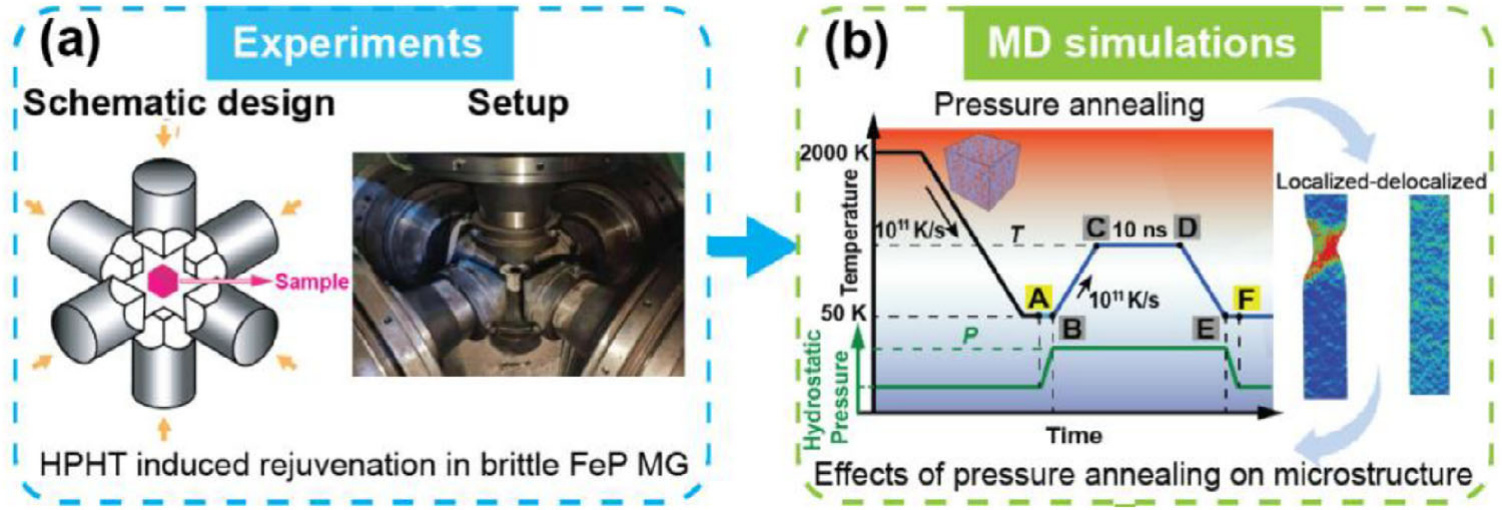
Schematic setup of high‐pressure rejuvenation experiment and followed the MD simulations [31]: (a) setup of high temperature and high pressure experiments; (b) setup of pressure annealing by MD simulations. Reprinted from ref. [31], copyright (2021), with permission from Elsevier Ltd Publishing Group.

The high‐pressure annealing (or rejuvenation) method refers to the process of applying elevated pressure on glassy alloys [3, 31, 32, 139]. High‐pressure‐induced rejuvenation of glassy alloys has been previously reported [94]. For instance, a glassy alloy rod with a diameter of 2 mm was successfully bent, as shown in Figure 7a,b. During the bending process, a pair of pincers was used in a straightforward manner, and the glassy rod was not intended to fracture. The entire bending operation was completed within just a few seconds. The appearance of the glassy rods before and after bending is presented in Figure 7a,b. Furthermore, the schematic setup for high‐pressure rejuvenation is illustrated in Figure 8. To provide a clear overview of experimental procedures, a few examples are given. Amorphous ribbons with a composition of Fe_80_P_20_, a width of 3 mm and a thickness of 30 µm, were fabricated using the single‐roller method [31]. These specimens were subjected to a pressure of 5.5 GPa at room temperature and subsequently heated to 573 K under the same pressure for 1 h, followed by quenching to room temperature. For bending rejuvenation, the glassy samples were bent to a curvature corresponding to an inner radius of approximately 7 mm [94].

##### 3.1.3.2 2 Experimental Results

The Pd‐Si BMGs subjected to bending deformation exhibit a hierarchical structural heterogeneity [94]. The microstructures formed on two sides of the bent alloys differ, likely due to the applied distinct stresses. Specifically, the inner side experiences compressive loading, while the outer side undergoes tensile loading, as shown in Figure 7b. A cooperative structural rearrangement, spanning from the atomic to the nanoscale, has been observed in these bent glassy alloys. The static structures revealed by high‐energy synchrotron X‐ray diffraction further confirm the deformation‐induced atomic rearrangements.

Pressure annealing can induce the transformation of glassy alloys into delocalized regions [31]. For instance, high temperature and pressure treatments have been applied to brittle Fe_80_P_20_ glassy alloys, and subsequent MD simulations confirmed the transition from localized domains to delocalized regions during high‐pressure annealing. In related studies, the rejuvenation effect of high pressure to Ce_65_Al_10_Co_25_ glassy alloys as well as the theoretical analysis of high‐pressure effects on hardening behavior and atomic dynamics has been also investigated [3, 32]. High‐pressure processing can also effectively tune the relaxation enthalpy. For example, the relaxation enthalpy of processed glassy alloys was reduced to ∼25.4 J/g, compared with 45.9 and 43.4 J/g for as‐cast and partially relaxed alloys, respectively [140]. Although the relaxation enthalpy decreases after high‐pressure treatment, the plasticity is significantly enhanced. The mechanism of pressure‐induced rejuvenation is believed to be associated with formation of higher‐energy secondary minima. In this context, the local cage dynamics plays a decisive role in governing structural relaxation under pressure‐compression conditions [32]. Simultaneous increases in atomic (packing) density and plasticity have been observed during high‐pressure processing [32], providing an additional pathway for tailoring microstructures. Moreover, strain hardening in the rejuvenated metastable state induced by high‐pressure compression has also been reported.

#### 3.1.4 Other Types of Mechanical Processing Methods

**FIGURE 9:**
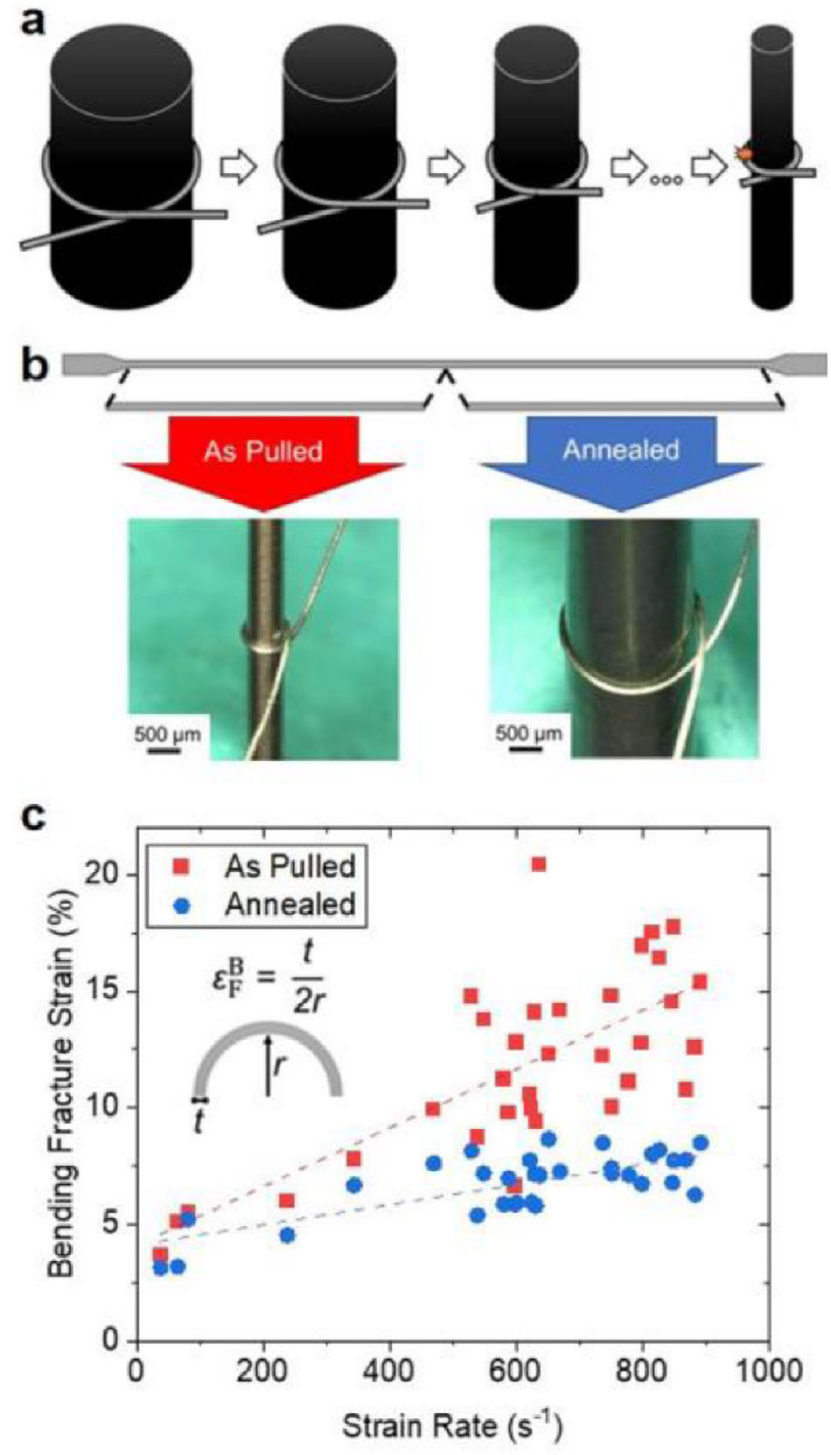
Correlation between bending fracture strain of Zr_44_Ti_11_Ni_10_Cu_10_Be_25_ BMG as a function of strain rate [141]: (a) wires are mechanically characterized through bending around cylinders of successively small diameter until fracture; (b) two types of wires that are characterized two processed alloys: one is as‐pulled and the other is as‐annealed; (c) for as pulled glassy alloys, a positive correlation between strain rate and bending fracture ductility has been found. By contrast, as annealed alloys have very small enhancement of ductility. Reprinted from ref. [141], copyright (2021), with permission from Springer Nature Publishing Group.

Both high strain rate and immediately rapid cooling have been employed to rejuvenate amorphous alloy Zr_44_Ti_11_Ni_10_Cu_10_Be_25_ [141]. The liquid state excited by applied strain rate can be preserved during the subsequent rapid cooling, thereby producing a rejuvenated glassy state with higher‐energy structural configurations. To investigate this effect, both as‐pulled (processed under strain rate and rapid cooling) and as‐annealed glassy alloys were prepared under identical processing parameters, as shown in Figure 9. It is evident that the as‐pulled alloys exhibit a clear positive correlation between bending fracture ductility and strain rate, whereas the as‐annealed ones show only a slight positive correlation. This rejuvenation approach can be classified as a thermo‐mechanical method. A key feature of this technique lies in achieving an ultrahigh cooling rate by thinning the glassy alloys within the Supercooled Liquid Regions (SCLRs). Such a high cooling rate effectively preserves portions of the excited and dilated liquid structures into the solid state.

Mechanical milling is a relatively simple technique that has been applied in several fields, such as fabrication of amorphous alloys from crystalline counterparts [142]. This method has also been reported to introduce a large amount of stored energy into both glassy alloys and crystalline materials [142]. For instance, a glassy alloy with the nominal composition Cu_40.6_Ti_37.5_Zr_9.4_Ni_9.4_Sn_3.1_ was found to reach an extremely rejuvenated state after mechanical milling. This highly rejuvenated glassy alloy exhibits remarkable resistance to long‐term aging, which may be attributed to the formation of a nanoglass‐like stable structures during the milling process. Specifically, the stored energy in the glassy powders increases with milling time, reaching a maximum enthalpy of 2.424 kJ/mol at 40 h (compared with 0.588 kJ/mol for as‐cast state). This maximum stored enthalpy remains nearly constant up to 100 h of milling. This study thus revealed that the stability of stored energy depends strongly on milling time. However, such a highly stable rejuvenated state has so far been achieved only in glassy powders. To date, no study has systematically investigated the stability of stored energy in bulk glassy alloys as a function of time.

### 3.2 Effects of Alloy Composition and Processing Parameters

#### 3.2.1 Effect of Alloy Composition

Alloy composition plays a crucial role in many aspects of amorphous alloys. For instance, atomic clusters are closely correlated with alloy composition, which in turn determines the glass‐forming ability (GFA) [16, 17]. During mechanical processing, the original atomic clusters in different glassy alloys undergo structural changes. Several examples have been reported to illustrate the relationship between alloy composition and mechanical properties after processing [38, 60, 131, 143, 144, 145, 146, 147]. In particular, the effect of composition on mechanical rejuvenation during HPT has been investigated [60]. Results revealed strain‐softening and improved plasticity in both rejuvenated Zr‐based glassy alloys Zr_55_Cu_30_Ni_5_Al_10_ and Zr_65_Cu_18_Ni_7_Al_10_. Notably, alloys with a lower Zr/Cu ratio are more easily rejuvenated. Specifically, compared with Zr_65_Cu_18_Ni_7_Al_10_, the Zr_55_Cu_30_Ni_5_Al_10_ alloy exhibits more pronounced strain‐softening behavior and deformation delocalization under HPT. These mechanical responses are likely linked to inherent structural features, such as the distribution of icosahedral clusters. Another example involves La‐ and Zr‐based BMGs, where La‐based alloys exhibit a higher rejuvenation ability than the Zr‐based counterparts [131]. Importantly, chemical composition also governs the degree of rejuvenation [143, 144, 145, 146]. For instance, alloys with a large negative mixing enthalpy and high mismatch entropy generally show greater relaxation enthalpy [143]. To verify this correlation, six different alloy compositions (Cu_50_Zr_50_, Ni_50_Zr_50_, Cu_40_Zr_50_Al_10_, Cu_40_Zr_50_Ag_10_, Al_60_Fe_40_, and Al_70_Ni_15_Co_15_) were examined. It's shown that both GFA and rejuvenation mechanisms originate from inherited structural features of the quenched melts. Thus, the compositional effect on mechanical rejuvenation can be primarily attributed to tuning short‐ and medium‐range orders (SROs and MROs), which is consistent with the influence of other rejuvenation strategies.

In a striking example with Fe_39_Ni_39_B_12.8_2Si_2.75_Nb_2.3_P_4.13_ BMG, fluxing treatment enhanced the plastic strain from 10.7 to over 50% [144]. This remarkable improvement was attributed to optimization of atomic‐scale structures, specifically the increase of icosahedral‐like clusters (ILCs) and crystal‐like clusters (CLCs), accompanied by a reduction in coordination number and MROs. Similarly, altering alloy composition by increasing Zr content modifies the fraction of Zr‐Zr atomic pairs [145]. With higher Zr content, both fracture strength and plastic strain increase, reaching 1816 MPa and 7.4%, respectively. The authors [145] suggested that excess Zr suppresses formation of full icosahedral clusters, which otherwise has a detrimental effect on mechanical properties of Zr‐Cu‐Al BMGs. A similar trend of increasing the MRO number is observed with reduced Cu content [146]. Finally, compared with the relatively ductile Zr‐based alloys, the brittle La‐based alloy (La_0.5_Ce_0.5_)_65_Co_25_Al_10_ demonstrates a higher degree of rejuvenation [131]. Collectively, these studies further indicate that alloy composition strongly influences rejuvenation behavior, structural ordering, and ultimately the mechanical performance of amorphous alloys.

#### 3.2.2 Effect of Processing Parameters

##### 3.2.2.1 Rotation Speed of HPT

It has been reported that degree of deformation strain or plastic strain is correlated with rotation speeds during HPT [61]. A Zr‐based BMG with the composition Zr_52.5_Cu_17.9_Ni_14.6_Al_10_Ti_5_ was subjected to HPT processing under a quasi‐hydrostatic pressure of 6 GPa at room temperature, with different numbers of rotation (*N* = 0, 10, 20, and 40) at a rotation speed of 0.6 rpm. It's demonstrated that deformation behaviors of the amorphous alloy are strongly dependent on numbers of applied rotations. In particular, at *N* = 40, strain localization is markedly suppressed, leading to a reduced yield stress and enhanced tensile plasticity. Another noteworthy observation is the variations in relaxation enthalpy with rotation number [60]. Specifically, the relaxation enthalpy (Δ*H*_rel_) of Zr_55_Cu_30_Ni_5_Al_10_ increases from 85 J/mol in as‐cast state to 761 J/mol after 50 rotations.

##### 3.2.2.2 Applied Stress and Amplitude of Compressive Loading

Different loading stresses and orientations (in two or three alternating directions) have been applied to process glassy alloys via MD simulations [13]. A change in loading orientation leads to an increase in potential energy of treated samples [14]. Mechanical cycling with varying amplitudes has also been employed to modulate the local structures of glassy alloys [14]. Moreover, increasing the intensity of mechanical cycling through higher stress amplitude, stress rate, or mean stress introduces additional energy into the alloys, thereby inducing structural rejuvenation. It should be noted that dynamic cyclic loading can activate frozen flow defects, and the extent of frozen rejuvenated regions can be controlled by adjusting the shock stress amplitude [95]. Thermo‐mechanical creep (anelastic creep strain) has also been shown to induce rejuvenation in glassy alloys (e.g., Zr_55_Cu_30_Ni_5_Al_10_), whereas plastic strain does not produce a comparable effect [148]. A higher creep stress results in a greater degree of structural rejuvenation. Similarly, twin‐roll casting has been demonstrated to modify the rejuvenation state of glassy alloys (e.g., Zr_41.2_Ti_13.8_Cu_12.5_Ni_10_Be_22.5_) [121].

##### 3.2.2.3 Processing Time and Impact Velocities

During shock compression, the stored relaxation enthalpy can be fully released within an extremely short time (on the order of ∼365 ns) [95]. The applied impact velocities are 270, 360, 480, and 520 m/s, respectively. Different velocities lead to distinct levels of relaxation enthalpy and mechanical properties at corresponding processing times. Both the modification of free volume at the atomic scale and structural rearrangements at the nanoscale contribute to the ultrafast rejuvenation of amorphous alloys [95]. To accurately describe the time scale of rejuvenation, authors [95] introduced a Deborah number (De) as a characteristic timescale parameter. When De<1, rejuvenation of the amorphous alloy is likely to occur, whereas De>1 indicates insufficient processing time for rejuvenation. Notably, when De≪1 (e.g., ∼0.1), structural rejuvenation can take place very rapidly. Furthermore, the relaxation enthalpy increases with increasing impact velocity (or shock stress and processing time). Specifically, the relaxation enthalpy rises from 0.423 kJ/mol in the as‐cast alloy to 0.712, 0.797, 1.170, and 1.321 kJ/mol at 270, 360, 480, and 520 m/s, respectively. This remarkable enhancement in rejuvenated states is primarily attributed to anelastic deformation. Authors [95] argued that adiabatic heating associated with plasticity plays only a minor role in enthalpy increase, while higher impact velocities contribute more significantly to the relaxation enthalpy.

### 3.3 Hardness and Elastic Modulus

The micro‐hardness and elastic modulus generally exhibit similar noticeable variation trends in rejuvenated glassy alloys [45, 51, 101]. For instance, in Zr_55_Cu_30_Ni_5_Al_10_ and Zr_65_Cu_18_Ni_7_Al_10_ alloys subjected to HPT, increasing the number of rotations up to 50 leads to a reduction in both elastic modulus and micro‐hardness. However, the decrease is less pronounced in Zr_65_Cu_18_Ni_7_Al_10_, suggesting that Zr_55_Cu_30_Ni_5_Al_10_ undergoes more significant strain softening and deformation delocalization during HPT [60]. At lower rotation numbers, the hardness variations are relatively small, whereas further plastic deformation results in a more distinct hardness evolution. In addition to the number of HPT rotations, the radial distance from the center also influences hardness evolution. However, the effect of radial distance is less significant compared with that of rotation numbers. Relative to the as‐cast state, hardness can decrease by as much as 20% in highly deformed regions. For example, alloys with radial distances of 40, 110, 320, and 490 µm exhibit hardness values of 6.17 ± 0.45, 7.48 ± 0.20, 7.29 ± 0.13, and 7.56 ± 0.23 GPa, respectively.

A lower hardness, localized softening, and homogeneous deformation have been observed in rejuvenated glassy alloys subjected to HPT treatment [31]. The reduction in hardness is generally attributed to increased mean atomic volume [29]. Moreover, heterogeneity in rejuvenated alloys has been reported, along with formation of highly strain‐softened regions during HPT. Such heterogeneity is reflected not only in hardness but also in elastic properties [29]. Interestingly, the hardness of soft regions and hard domains remains comparable for alloys processed under both 4 and 8 GPa [29]. For alloys previously annealed and then subjected to lateral elasto‐static preloading for 1 h (LP1h), hardness shows a clear dependence on the distance from the center [28]. With the partially annealed (PA)+LP1h condition, the regions near the center exhibit higher hardness than the as‐cast state, whereas regions farther from the center show lower hardness. In contrast, for PA + LA1h alloys, the hardness near the center is also higher than that in the as‐cast sample, but it becomes nearly identical to that of the as‐cast alloy when the distance from the center exceeds 0.75 mm. For a Zr_50_Cu_40_Al_10_ glassy alloy processed by HPT with 50 rotation cycles, both hardness and elastic modulus decrease significantly (hardness ∼4.9 vs. 6.1 GPa in as‐cast, elastic modulus ∼76.1 vs. 103.9 GPa in as‐cast), which can be ascribed to the emergence of softened regions with lower local elastic modulus [26]. Similarly, compared to as‐cast La‐based glassy alloys, deformed ones show a reduction in hardness by about 7% [131]. In order to have a clear map of evolution of hardness and elastic modulus together with relaxation enthalpy under varied processing parameters, a lot of related correlations can be found in Table 1.

**TABLE 1:** Hardness and elastic modulus as well as relaxation enthalpy as a function of processing parameters.

Alloy composition	Processing parameters	Hardness (HV)	Hardness (GPA)	Elastic modulus (GPa)	Relaxation enthalpy (J/mol)	Relaxation enthalpy (J/g)	References
Zr_55_Cu_30_Ni_5_Al_10_	HPT: N = 50	5.63± 0.24		92.5± 2.3	761		[60]
	As‐cast one	6.87± 0.17		105± 0.24	85		[60]
Zr_65_Cu_18_Ni_7_Al_10_	HPT: N = 50	5.46± 0.17		91.0± 2.1	492		[60]
	As‐cast one	5.85± 0.19		94.7± 2.7	64		[60]
Zr_52.5_Cu_17.9_Ni_14.6_Al_10_Ti_5_	HPT: N = 40	5.7± 0.3		65.1± 1.2			[61]
	As‐cast one	7.05± 0.3		99.0± 0.7			[61]
Cu_45_Zr_45_Al_5_Ag_5_	HPT:N = 80, 4GPa	4.87± 0.1					[62]
		5.62± 0.18					
	As‐cast one	5.95± 0.16					[62]
Cu_50_Zr_50_	Preloaded to 90%σ_*y*_			77.6± 3.0		5.26	[124]
	As‐cast one			82.5± 1.5		4.70	[124]
Cu_57_Zr_43_	Preloaded to 90%σ_*y*_			87.6± 2.0		5.53	[124]
	As‐cast one			99.0± 4.0		3.85	[124]
Cu_65_Zr_35_	Preloaded to 90%σ_*y*_			98.0± 2.8		5.38	[124]
	As‐cast one			115.5± 2.5		3.00	[124]
Zr_44_Ti_11_Ni_10_Cu_10_Be_25_	Strain rate at 215/s				210		[141]
Zr_44_Ti_11_Ni_10_Cu_10_Be_25_	Strain rate at 830/s				810		[141]
Pd_43_Cu_27_Ni_10_P_20_	EL for 48 h				307± 28		[125]
Zr_52.5_Cu_17.9_Ni_14.6_Al_10_Ti_5_	EL for 72 h				415± 21		[125]
Zr_55_Cu_30_Al_10_Ni_5_	As‐cast					115.3	[126]
Zr_55_Cu_30_Al_10_Ni_5_	Load at 5% of σ_*y*_					115.9	[126]
Zr_55_Cu_30_Al_10_Ni_5_	Load at 10% of σ_*y*_					117.1	[126]
Zr_55_Cu_30_Al_10_Ni_5_	Load at 15% of σ_*y*_					118.2	[126]
Zr_55_Cu_30_Al_10_Ni_5_	Load at 20% of σ_*y*_					118.5	[126]
Zr_64.13_Cu_15.75_Ni_10.12_Al_10_	As‐cast one		495± 5		490		[63]
Zr_64.13_Cu_15.75_Ni_10.12_Al_10_	20%plastic strain		460± 12				[63]
Zr_64.13_Cu_15.75_Ni_10.12_Al_10_	40%plastic strain		401± 14		1130		[63]
Zr_55_Cu_30_Al_10_Ni_5_	As‐cast one				410		[63]
Zr_55_Cu_30_Al_10_Ni_5_	Load at 5% of σ_*y*_				470		[63]
Zr_55_Cu_30_Al_10_Ni_5_	Load at 10% of σ_*y*_				600		[63]
Zr_55_Cu_30_Al_10_Ni_5_	Load at 15% of σ_*y*_				710		[63]
Zr_50.7_Cu_28_Ni_9_Al_12.3_	As‐cast one	∼4.8				2.0	[64]
Zr_50.7_Cu_28_Ni_9_Al_12.3_	HPT: 6 GPa 1 R	∼4.2				3.8	[64]
Zr_35_Ti_30_Be_27.5_Cu_7.5_	As‐cast one					3.935	[128]
Zr_35_Ti_30_Be_27.5_Cu_7.5_	Load at 50% of σ_*y*_					2.967	[128]
Zr_35_Ti_30_Be_27.5_Cu_7.5_	Load at 70% of σ_*y*_					1.211	[128]
Zr_35_Ti_30_Be_27.5_Cu_7.5_	Load at 70% of σ_*y*_					5.299	[128]
Zr_41.2_Ti_13.8_Cu_12.5_Ni_10_Be_22.5_	Single‐roll: 40µm	6.17± 0.45			1253± 30		[121]
Zr_41.2_Ti_13.8_Cu_12.5_Ni_10_Be_22.5_	Single‐roll: 110µm	7.48± 0.2			976± 12		[121]
Zr_41.2_Ti_13.8_Cu_12.5_Ni_10_Be_22.5_	Twin‐roll: 320µm	7.29± 0.13			1050± 24		[121]
Zr_41.2_Ti_13.8_Cu_12.5_Ni_10_Be_22.5_	Twin‐roll: 490µm	7.56± 0.23			917± 12		[121]
La_55_Al_25_Ni_20_	37% strain				230		[137]
La_55_Al_25_Ni_20_	55% strain				500		[137]
La_55_Al_25_Ni_20_	85% strain				670		[137]
Zr_55_Cu_30_Al_10_Ni_5_	As‐cast one	7.23± 0.11		93.0± 0.5		6.20± 0.30	[130]
Zr_55_Cu_30_Al_10_Ni_5_	ESL 40% of σ_*y*_					7.46± 0.45	[130]
Zr_55_Cu_30_Al_10_Ni_5_	ESL 70% of σ_*y*_					9.71± 0.25	[130]
Zr_55_Cu_30_Al_10_Ni_5_	ESL 80% of σ_*y*_					11.63± 0.20	[130]
Zr_55_Cu_30_Al_10_Ni_5_	ESL 90% of σ_*y*_	6.59± 0.11		87.1± 0.11		13.20± 0.40	[130]
(La_0.5_Ce_0.5_)_65_Co_25_Al_10_	As‐cast one					1.159	[131]
(La_0.5_Ce_0.5_)_65_Co_25_Al_10_	Notch 2.6% plasticity					1.391	[131]
(La_0.5_Ce_0.5_)_65_Co_25_Al_10_	Notch 4.8% plasticity					1.668	[131]
La_55_Al_25_Ni_5_Cu_10_Co_5_	As‐cast one	3		45.6	180		[25]
La_55_Al_25_Ni_5_Cu_10_Co_5_	5% cold rolling	2.5		35.2	210		[25]
La_55_Al_25_Ni_5_Cu_10_Co_5_	20% cold rolling	2.0		31.2	290		[25]
Zr_50_Cu_40_Al_10_	As‐cast one	6.1± 0.1		103.9± 1.5			[26]
Zr_50_Cu_40_Al_10_	HPT: rotation = 1						[26]
Zr_50_Cu_40_Al_10_	HPT: rotation = 10						[26]
Zr_50_Cu_40_Al_10_	HPT: rotation = 20						[26]
Zr_50_Cu_40_Al_10_	HPT: rotation = 50	4.9± 0.2		76.1± 1.4			[26]
Zr_64.13_Cu_15.75_Ni_10.12_Al_10_	As‐cast					4.84	[28]
Zr_64.13_Cu_15.75_Ni_10.12_Al_10_	PA‐ESL + 0.5h					2.85	[28]
Zr_64.13_Cu_15.75_Ni_10.12_Al_10_	PA‐ESL + 1h					3.28	[28]
Zr_64.13_Cu_15.75_Ni_10.12_Al_10_	PA‐ESL + 2.5h					2.28	[28]
Zr_64.13_Cu_15.75_Ni_10.12_Al_10_	PA‐ESL + 4h					2.43	[28]
Zr_64.13_Cu_15.75_Ni_10.12_Al_10_	PA‐ESL + 7h					1.07	[28]
Zr_64.13_Cu_15.75_Ni_10.12_Al_10_	PA‐ESL + 10h					0.66	[28]
Cu_45_Zr_45_Al_5_Ag_5_	As‐cast	5.95± 0.16		103± 1	380± 0.07		[29]
Cu_45_Zr_45_Al_5_Ag_5_	20turns/8 GPa/LNT	5.30± 0.23		93.9± 2.5	1140± 0.07		[29]
Cu_45_Zr_45_Al_5_Ag_5_	80turns/4GPa	S:4.87± 0.10		86.3± 2.5	1910± 0.15		[29]
Cu_45_Zr_45_Al_5_Ag_5_		H:5.62± 0.21		96.7± 2.0			[29]
Cu_45_Zr_45_Al_5_Ag_5_	80turns/8GPa	S:4.87± 0.18		87.1± 3.3	1370± 0.07		[29]
Cu_45_Zr_45_Al_5_Ag_5_		H:5.58± 0.18		98.0± 3.3			[29]
Pd_40_Ni_40_P_20_	As‐cast					2.92	[33]
Pd_40_Ni_40_P_20_	5 rotation/12GPa					6.61	[33]
Pd_40_Ni_40_P_20_	10 rotation/12GPa					6.27	[33]
Pd_40_Ni_40_P_20_	20 rotation/12GPa					6.25	[33]
Pd_40_Ni_40_P_20_	50 rotation/12GPa					6.84	[33]
Pd_40_Ni_40_P_20_	100 rotation/12GPa					10.04	[33]
Zr_55_Cu_30_Ni_5_Al_10_	As‐cast				423		[95]
Zr_55_Cu_30_Ni_5_Al_10_	SC: 270m/s				712		[95]
Zr_55_Cu_30_Ni_5_Al_10_	SC: 360m/s				797		[95]
Zr_55_Cu_30_Ni_5_Al_10_	SC: 480m/s				1170		[95]
Zr_55_Cu_30_Ni_5_Al_10_	SC: 520m/s				1321		[95]

### 3.4 Atomic Clusters

**FIGURE 10:**
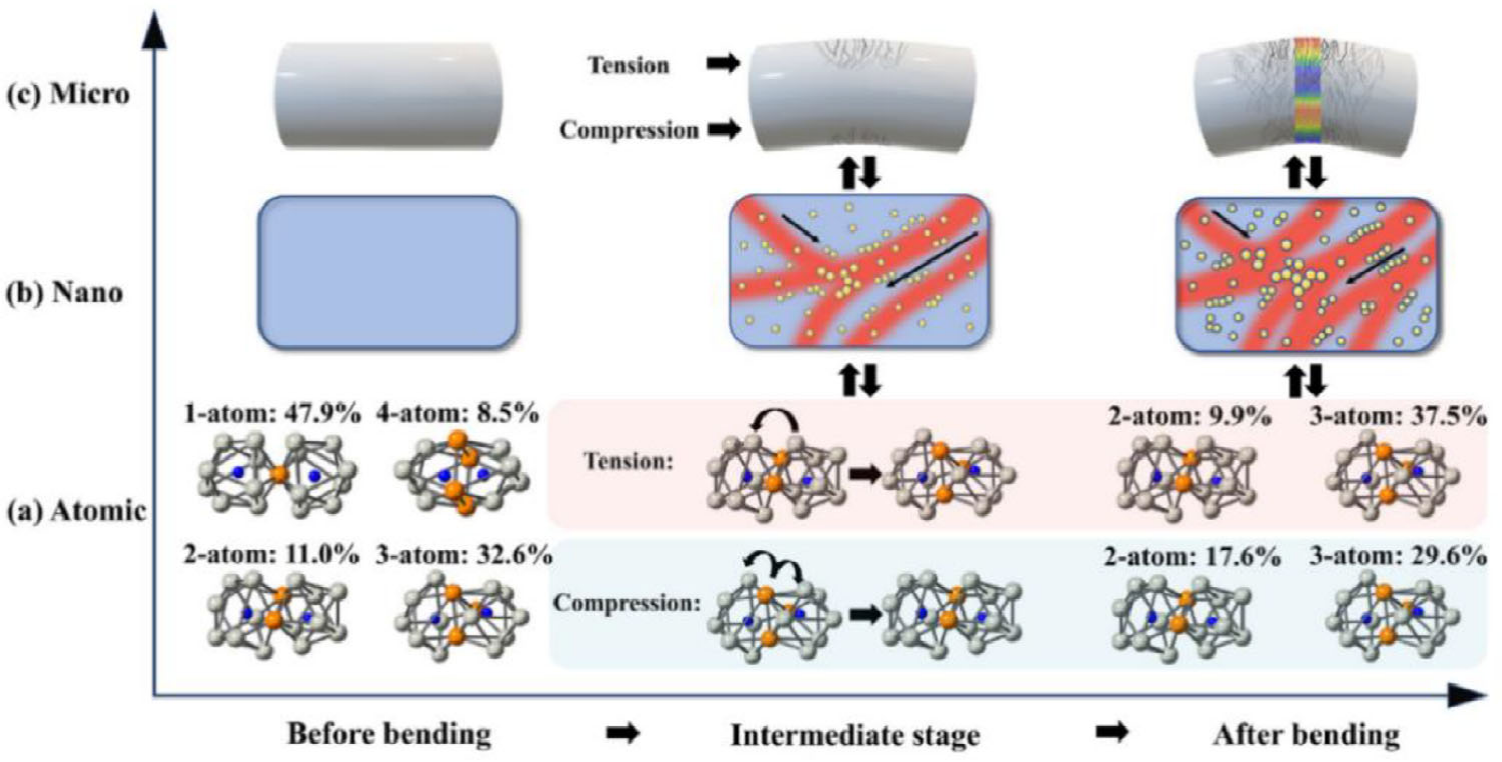
Schematic diagram of the evolution of (a) atomic‐, (b) nano‐, and (c) micrometer‐scale heterogeneity for the Pd‐Si glassy alloys before bending/deformation, intermediate stage, and after bending/deformation [94]: (a) for the atomic‐scale, the connection modes of 2‐, 3‐atom in the bending process of the tensile region and the compression region are mutually transformed. For the convenience of observation, the Pd‐Si and Si‐Si bonds are omitted; (b) the mobility of atoms during deformation, and especially in the shear bands, may be enhanced, resulting in the accumulative of nano‐scale heterogeneous structures in shear bands and especially in their convergence; (c) along with the bending process, there are abundant of multiple shear bands on both sides. The heterogeneous stress distribution from the tensile side to the compression side can be found in the color band in the bent samples. Reprinted from ref. [94], copyright (2020), with permission from Elsevier Ltd Publishing Group.

Different types of atomic clusters are critical structural features in rejuvenated glassy alloys [94]. The structural disordering associated with rejuvenation occurs across multiple length scales of atomic rearrangements [95], which can be further tuned by SPD [30] and lateral ESL [124]. The rate of disordering is largely governed by the atomic packing density, determined by SRO clusters. Specifically, the types and fractions of both SROs and MROs can be modified under lateral ESL compression. For instance, compared with Cu_50_Zr_50_ and Cu_57_Zr_43_, the Cu_65_Zr_35_ alloy contains a relatively higher fraction of densely packed SRO clusters such as <0,0,12,0> and <0,1,12,0>, and a lower fraction of loosely packed clusters such as <0,3,6,4>, <0,3,6,5>, and <0,3,6,6>. The presence of various structural heterogeneities likely arises from differences in the arrangement of SROs and MROs, and distinct types of MRO structures have been observed in different regions of the bent glassy alloy (Figure 10). It can be also found that the number of atoms connecting or constituting different clusters (SROs or MROs) varies under compression and tension. Generally, tensile loading promotes the transformation of 2‐atom into 3‐atom connected clusters, whereas compressive loading tends to drive the opposite transformation.

The transition from relaxation to rejuvenation is closely correlated with changes in local atomic symmetry [128, 129]. During relaxation, the bond‐orientational order (BOO) decreases while five‐fold local symmetry increases [128]. In contrast, rejuvenation generally enhances BOO and reduces five‐fold symmetry. In other words, both BOO and five‐fold symmetry can be regarded as one type of SROs and MROs. Accordingly, the occurrence of relaxation and rejuvenation is intrinsically linked to evolution of SROs and MROs. Notably, the aged glassy alloy Zr_64.13_Cu_15.75_Ni_10.12_Al_10_ can be rejuvenated through ESL [129]. Such structural rejuvenation, accompanied by dilatation at short‐ and medium‐range atomic scales (i.e., volumetric expansion induced by increased average atomic bond length) has been successfully achieved under compressive ESL treatment.

**FIGURE 11:**
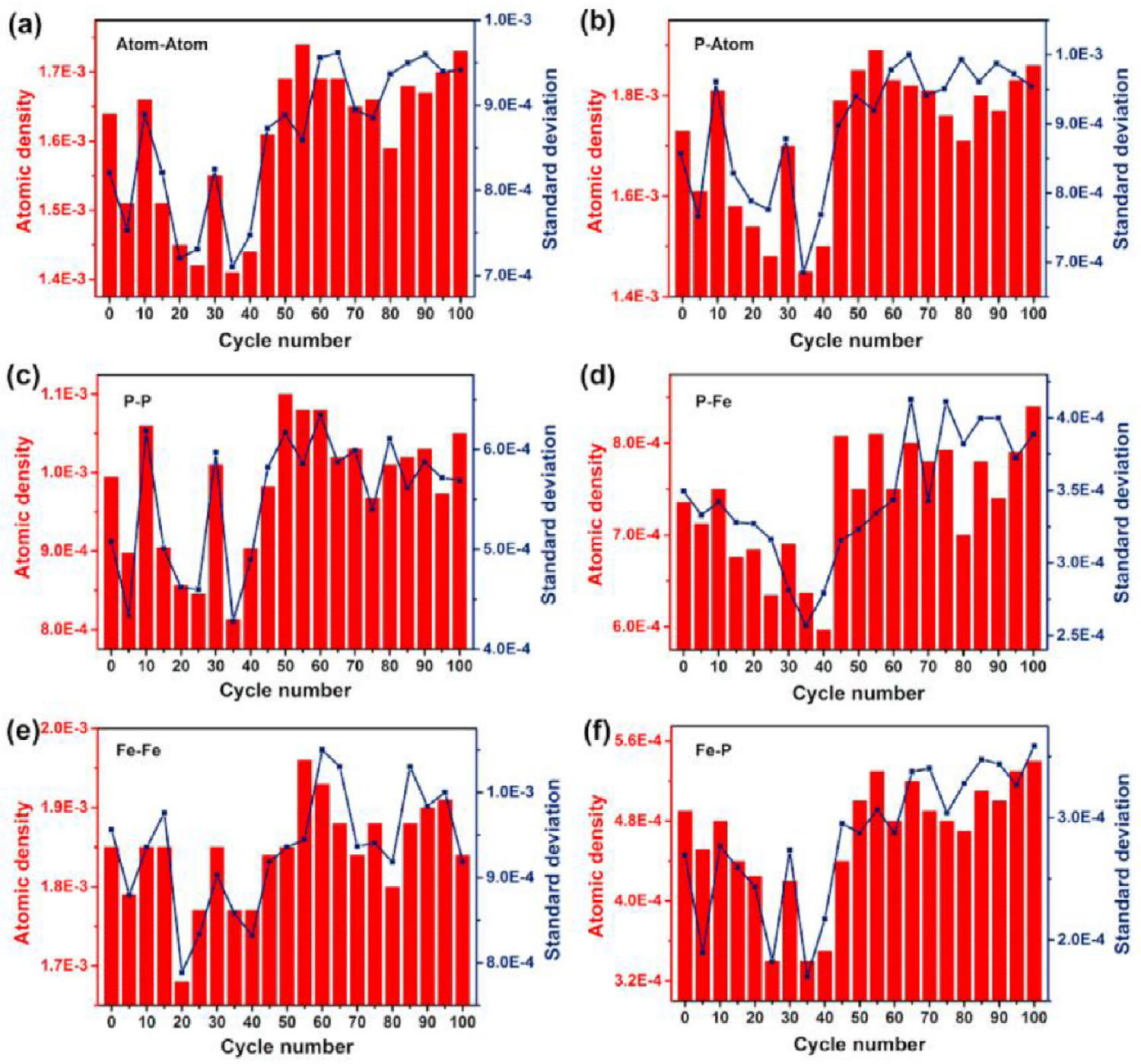
Atomic density as a function of cycling number [147]: (a) all atoms; (b) atoms around P atoms; (c) P atoms around P atoms; (d) Fe atoms around P atoms; (e) atoms around Fe atoms; and (f) P atoms around Fe atoms. The radius value used for the calculation is 8 Å. Reprinted from ref. [147], copyright (2021), with permission from Elsevier BV Publishing Group.

The structural disordering can be significantly enhanced by combined effects of pressure and temperature annealing [31, 139, 147]. Pressure annealing has been also shown to induce structural rejuvenation in Fe_80_P_20_ glassy alloys, leading to multiple variations in atomic packing structures [147]. Moreover, it was reported that increasing the pressure during annealing can trigger a transition from localized to delocalized deformation. For rejuvenated Fe‐P glassy alloys, the observed delocalized deformation behavior arises from reduced atomic density fluctuations and the presence of dispersed clusters of P atoms surrounded by Fe atoms. Corresponding simulation studies further confirmed that pressure annealing indeed alters the atomic packing structures [31, 147]. For instance, the redistribution of P atoms modifies their spatial correlations and distributions, thereby influencing deformation mechanisms, as illustrated in Figure 11. At higher annealing pressures, the otherwise brittle Fe‐P glassy alloy becomes rejuvenated, accompanied by an increase in atomic packing density [31].

The densely packed structures can lead to a high degree of atomic coordination. For instance, with increasing pressure, the fractions of clusters such as <0,0,12,0>, <0,2,8,2>, and <0,1,10,2> increase, while those of clusters like <0,2,8,1> and <0,3,6,3> decrease [38]. In glassy alloys of Cu_64_Zr_36_, Cu_50_Zr_50_, and Cu_46_Zr_54_, a higher Cu content tends to suppressed rejuvenation, which is accompanied by increased fraction of <0,0,12,0> polyhedra. This indicates that the degree of rejuvenation can be strongly affected by alloy composition, particularly the Cu concentration. A previous study reported that the transition from localization to delocalization occurs when the pressure during annealing exceeds 10 GPa in CuZr glassy alloys [38]. In pressure‐induced rejuvenated alloys, the atomic structures become more homogeneous, leading to a reduction in spatial heterogeneity of atomic number density as both pressure and temperature increase [31]. Moreover, changes in atomic density may be associated with specific atomic species. For example, when the temperature rises from 300 to 1300 K under constant pressure, P atoms in FeP alloys are more sensitive to temperature variations than Fe atoms [147]. At pressures up to 30 GPa, the atomic volume distribution of P atoms narrows, suggesting that P atoms adopt smaller atomic volumes. Additionally, higher pressures lead to the shrinkage of large cavities [147].

## 4 Thermal Processing to Induce Rejuvenation

Thermal rejuvenation is another approach that can be employed to modify microstructures and mechanical properties of glassy alloys. This processing strategy encompasses several methods such as CTC or DCT, ultrafast heating and cooling of glassy alloys (flash annealing), and thermal cycling at temperatures above room temperature. These techniques are designed to stabilize altered microstructures, thereby enhancing plasticity and promoting work‐hardening behavior. However, it should be noted that the effectiveness of thermal rejuvenation strongly depends on the specific processing parameters that play distinct roles in modifying the microstructures, particularly at the nano‐scale or even atomic‐scale. One of the most significant advantages of thermal rejuvenation is overcoming the size limitations associated with mechanical rejuvenation that greatly hinder the practical application of glassy alloys.

### 4.1 Main Types of Thermal Rejuvenation

#### 4.1.1 Cryogenic Thermal Cycling

##### 4.1.1.1 Experimental Design and Operations

The CTC or DCT process, also referred to as differential thermal cycling (DCC), has been widely employed to modify microstructures of glassy alloys [40, 41, 45, 49, 51, 53, 55, 67, 72, 147, 149, 150, 151, 152, 153, 154, 155, 156, 157, 158]. In a typical CTC treatment, alloys are first immersed in liquid nitrogen for a certain period (e.g., 1 min), and subsequently exposed to various heating conditions such as airflow at room temperature [40, 41, 45, 49, 72, 147, 150], boiling water [67], absolute alcohol or high‐temperature silicone oil (ranging from 353 to 563 K) for a controlled duration (e.g., 1 min) [51, 53]. In addition to these general procedures, CTC experiments with more specific processing parameters were designed in some studies as summarized in Table 2.

**TABLE 2:** Summary of Processing Parameters Regarding the CTC/DCT Experiments in Some Previous Studies.

Alloy composition	Operation parameters	References
Zr_61_Cu_25_Al_12_Ti_2_	Flash annealing, heating rate of 265± 35 K/s	[39]
Pd_43_Cu_27_Ni_10_P_20_	DCT: LN and RT for 1 min, up to 200cycles	[40]
Zr_59.5_Nb_4.8_Cu_14.4_Ni1_1.6_Al_9.7_	DCT: LN for 5 min and boiling water for 3 min and then RT for 1 min, up to 40 cycles	[67]
La_62_Al_14_Ag_2.34_Ni_10.83_Co_10.83_	DCT: LN for 2 min and alcohol of 303 K for 2 min, up to 120 cycling	[148]
Zr_47.5_Cu_47.5_Al_5_	DCT: LN for 1 min and RT for 1 min, up to 30 cycling	[49]
La_60_Ni_15_Al_25_	DCT: LN and 293/323/373K for up to 200 cycling	[50]
Fe‐Co‐B‐Si‐Nb‐Cu	DCT:LN for 1 min and silicone oil for 353, 373, 393, 433, 473 K for up to 15 cycling	[51, 53]
La_60_Ni_15_Al_25_	DCT: LN for 1 min and dryer for 1 min, up to 100 cycling	[45]

##### 4.1.1.2 Experimental Results

The CTC processing can induce both rejuvenation and relaxation, which can be distinguished from the corresponding microstructural features. Specifically, rejuvenation is likely associated with local atomic motion in loosely packed domains, whereas relaxation may originate from cooperative atomic motion in densely packed regions [45]. The microstructural evolution during CTC processing is highly dependent on processing parameters. For instance, glassy alloys have been subjected to thermal cycling between liquid nitrogen and different temperatures (293, 323, and 373 K) for specific durations [50]. Varying the numbers of thermal cycles leads to different degrees of structural disordering, which can be macroscopically reflected by changes in relaxation enthalpy. At a lower number of cycles, relaxation enthalpy tends to increase, whereas with further cycling, it decreases.

In one study, a FeCo‐based alloy exhibited enhanced plasticity of 7.4% and a yield strength of 4350 MPa after undergoing CTC between 393 K and cryogenic temperature [51]. The improvement in mechanical properties was attributed to rejuvenated microstructures with nanometer‐scale heterogeneities induced by Cu addition. Similarly, the alloy [(Fe_0.5_Co_0.5_)_0.75_B_0.2_Si_0.05_]_96_Nb_4_ processed by CTC exhibited a rejuvenated state with a plastic strain of 6.1% [53]. The enhancement was proposed to arise from a higher degree of structural disorder and the formation of more CLO structures. Furthermore, processing temperature played a critical role: increasing the CTC temperature from 433 to 513 K promoted plasticity, while a further increase to 563 K reduced it. Accordingly, the optimized rejuvenated state in FeCo‐based glassy alloys was achieved at 513 K. Additionally, DCT was reported to reduce the atomic spacing of MRO clusters in both as‐quenched and relaxed alloys [49]. Another study investigating CTC effects up to 800 cycles revealed that local atomic motion in loosely packed regions predominantly drives rejuvenation, while cooperative atomic motion in densely packed regions tends to result in relaxation [45].

The activation energy of *β* relaxation in three groups functions with processing temperature and numbers of thermal cycles [50]. Regarding the relaxation enthalpy (Δ*H_rel_*), it is higher than that of the as‐quenched alloys at CTC293, while Δ*H_rel_* at CTC373 is lower than that of the as‐quenched alloys [50]. This indicates that increasing the processing temperature does not necessarily promote rejuvenation. In another experiment using DCT, when the processing temperature increased from 433 to 553 K, Δ*H_rel_* initially rose but then dropped sharply at 563 K [53]. Similarly, with increasing cycle numbers, Δ*H_rel_* first increased to 5.964 J/g at DCT10, then decreased to 5.279 J/g at DCT20, which remained higher than that of the as‐quenched state (4.376 J/g) [150]. Another study on DCT cycling revealed that the Δ*H_rel_* of DCT30 reached 14.6 J/g, exceeding that of the as‐cast alloy (12.7 J/g) [91]. When the number of cycles exceeds 200, Δ*H_rel_* decreases further and becomes lower than that of rapidly quenched alloys [50].

**FIGURE 12:**
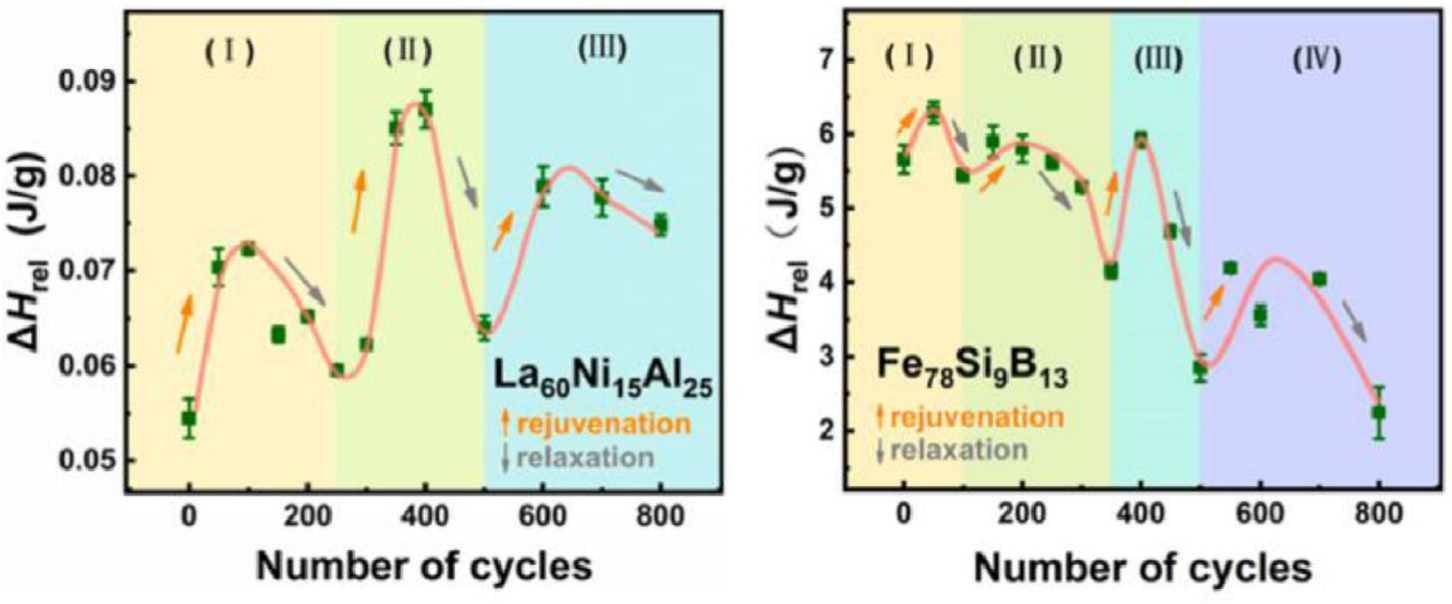
Variation of Δ*H_rel_* with error bars as a fucntion of numbers of cycles for as‐cast and CTC processed glassy alloys of (a) La_60_Ni_15_Al_25_; (b) Fe_78_Si_9_B_13_ [45]. Reprinted from ref. [45], copyright (2022), with permission from Elsevier BV Publishing Group.

Overall, Δ*H_rel_* shows a characteristic trend with processing temperature: it increases from the as‐quenched state to 393 K, reaches a peak, and then decreases with further temperature elevation. The evolution of enthalpy as a function of cycle numbers for two glassy alloys, La_60_Ni_15_Al_25_ and Fe_78_Si_9_B_13_, is presented in Figure 12. Alternating increases and decreases in enthalpy (corresponding to rejuvenation and aging) are clearly observed with increasing CTC.

#### 4.1.2 Thermal Cycling

##### 4.1.2.1 Experimental Design and Operations

**FIGURE 13:**
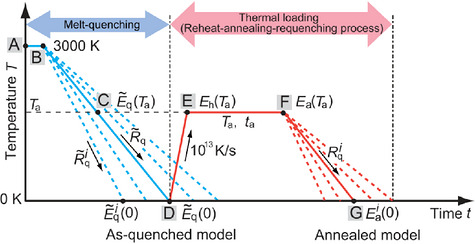
Description of the initial glass quenching and subsequent thermal processing [68]. To be specific, the initially quenching rate is at constant cooling rate of *R_q_* (B→D) and the *E_q_* stands for the instantaneous potential energy at the temperature of T in the initially quenched stage. And then the quenched glassy alloys were subjected to thermal processing: heating (D→E) and isothermal annealing (E→F) and quenching (F→G) under various annealing temperatures of *T_a_* and constant cooling rates of *R_q_*. The $E_{q}^{i}$ represents another instantaneous potential energy at temperature of T in the second quenching stage (F→G). Reprinted from ref. [68], copyright (2015), with permission from Springer Nature Publishing Group.

Thermal cycling was typically applied to heat as‐quenched glassy alloys to a specific temperature, with a heating rate faster than quenching rate of the original as‐cast alloys [68, 69, 159, 160, 161, 162]. The procedures of thermal cycling are illustrated in Figure 13. In this process, the glassy alloys were heated to a temperature above 1.1 *T_g_* and subsequently quenched to room temperature at a cooling rate higher than that used in the initial quenching [68]. Furthermore, Zr‐Cu‐Al‐Ag glassy alloys were subjected to bending tests at temperatures ranging from 77 to 573 K, and their plasticity was found to vary significantly with the processing temperature [69].

##### 4.1.2.2 Experimental Results

**FIGURE 14:**
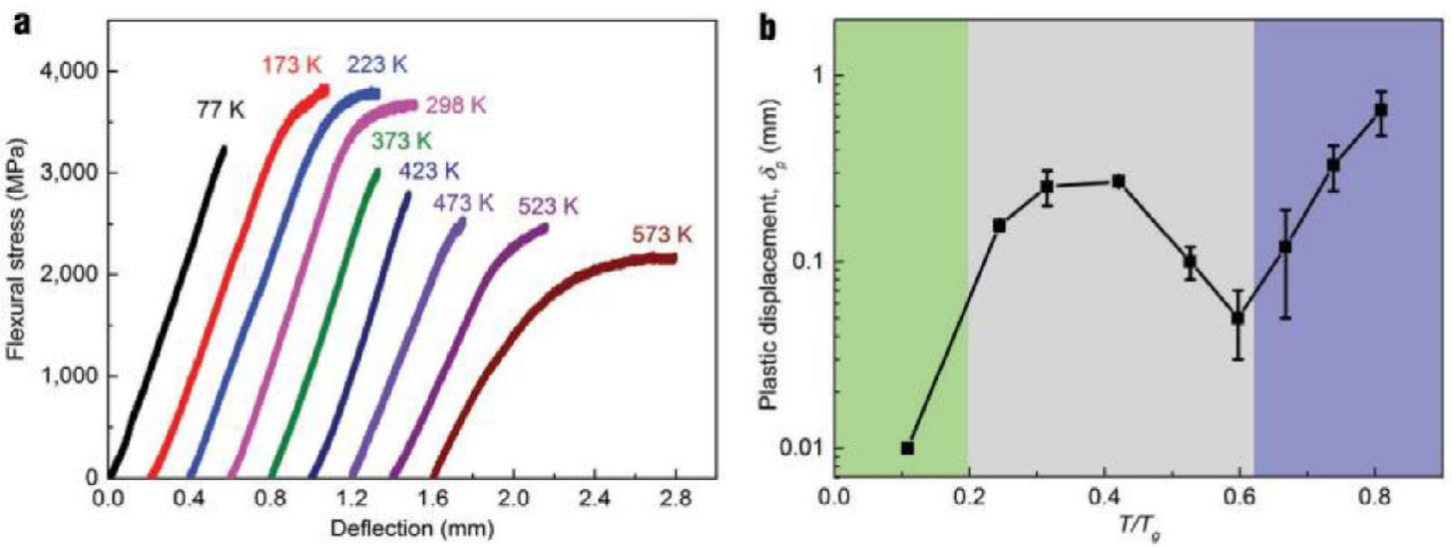
Mechanical properties of Zr_46_(Cu_4.5/5.5_Ag_1/5.5_)_46_Al_8_ with a thickness of 0.8 mm via the three bending test [69]: (a) at different testing temperature, the representative flexural stress‐deflection responses of ZrCu‐based alloys; (b) the plastic displacement as a function of $T/T_{g}$. Reprinted from ref. [69], copyright (2017), with permission from Wiley‐Blackwell Publishing Group.

The ductility of glassy alloys is highly sensitive to testing temperatures [69]. With increasing temperature, these alloys exhibit intermediate‐temperature brittleness. For the tested Zr_46_(Cu_4.5/5.5_Ag_1/5.5_)_46_Al_8_ alloy with a thickness of 0.8 mm under three‐point bending (Figure 14), the plastic displacement (or bending ductility) initially increases with rising *T*/*T_g_*, decreases at 0.6 *T*/*T_g_*, and then reaches its maximum at 0.8 *T*/*T_g_*. In this context, rejuvenation occurs at 0.4 *T*/*T_g_* and 0.8 *T*/*T_g_*, whereas structural relaxation is dominant at 0.6 *T*/*T_g_*. To further clarify effects of intermediate temperatures on structural states (rejuvenation or relaxation), Some glassy alloys such as Zr_46_Cu_38_Al_8_Ag_8_ glassy alloys were studied systematically to explore the relationship between rejuvenation and annealing near 0.9 *T_g_* for various durations [37, 56, 70]. The concept of enthalpy relaxation has been proposed by a few studies [37, 56] which reported an interesting finding. A relaxed glassy alloys annealed at temperature far below *T_g_* can be rejuvenated at temperature near *T_g_*. This type of rejuvenated glassy alloys has other peculiar features: the identified enthalpy approaches (lower than) that of as‐cast counterparts and is higher than annealed ones but the hardness is slightly higher than that of as‐cast ones, and plasticity of most processed glassy alloys processed by enthalpy relaxation is higher than that of as‐cast counterparts. Authors argue that it is the atomic density not the atomic structures that is responsible for enhanced mechanical properties. The studies regarding the enthalpy relaxation focus on two consecutive steps of thermal treatments: the first step is to anneal the as‐cast glassy alloys and the second step is to rejuvenate the annealed glassy alloys. Difference in this type of processing and intermediate‐temperature annealing [69, 76] might be located at the second rejuvenation step. The plasticity of annealed glassy alloys from low temperature to temperature near *T_g_* fluctuates: first increase, and then decrease and increase again. The possible origins indicated by studies [37, 56] might be changes of atomic clusters.

Other studies also made some investigations regarding correlation between plasticity and intermediate‐temperature annealing [69, 70, 76, 163]. Results demonstrated that the thermo‐cycling treatment can effectively rejuvenate short‐term annealed and relaxed alloys, but has little impact on long‐term annealed alloys. Specifically, thermal cycling reduces the potential energy of rapidly annealed glasses, while large cycling amplitudes increase the potential energy of slowly annealed glasses [71]. Thus, depending on cycling parameters (temperature or amplitude), either an overaged or a rejuvenated state can be induced. In another study [73], relaxed glassy alloys were heated above *T_g_* and subsequently cooled at different rates (100, 300, and 700 K/min) to retain the modified structures. Although all three samples exhibited nanometer‐sized heterogeneous structures, the volume fraction of nanoclusters varied with cooling rate. A slower cooling rate resulted in a higher volume fraction of nanometer‐sized clusters. Correspondingly, the enthalpy states of the samples differed, with higher final cooling rates leading to larger relaxation enthalpy.

The studies of enthalpy relaxation and intermediate‐temperature annealing make us to rethink that the decoupling between enthalpy and hardness, plasticity for rejuvenated glassy alloys. As reported by authors [56], the atomic density might be responsible for this decoupling. One study [164] studied correlation between internal states and plasticity via tuning the melting current. Authors measured density of quenched glassy alloys and found that density of alloys with same compositions are different and therefore differences in plasticity have been observed. The free volume might be one clue to explain changes in density and some other changes such as arrangements of atomic clusters might be another reason to be responsible for density changes. Previous and important studies regarding on atomic clusters of glassy alloys [9, 16, 17] indicated that significant differences/changes in atomic clusters if some changed processing parameters apply. According to knowledge of authors, the enthalpy, serving as a sole indicator of rejuvenation, cannot be applied to glassy alloys under multiple heat treatments, especially at intermediate‐temperatures annealing. These findings from enthalpy relaxation and intermediate‐temperature annealing indicated that some multiple heat treatments between a temperature far below *T_g_* and near *T_g_* might provide another route to rejuvenate glassy alloys and therefore enhance plasticity.

Modifying the fictive temperatures is another effective method to enhance the plasticity of glassy alloys [31, 36, 56, 165]. The fictive temperatures (*T_f_*) can be defined as the temperatures at which the frozen‐in liquid structure remains in equilibrium. To date, several studies have demonstrated a positive correlation between the *T_f_* and the rejuvenation features of glassy alloys [31, 56, 72, 165]. For La‐Al‐Ni glassy alloys, the fictive temperature is typically in the range of 1.04 *T_g_* – 1.1*T_g_* [165]. Within this range, increasing *T_f_* generally reduces the rejuvenation capacity. One study reported that after 200 thermal cycles, different glassy systems with distinct *T_f_* exhibited different structural states [72]. For instance, the Pd‐based BMGs with *T_f_* = 633 K showed relatively loosely packed structures, whereas the Zr‐based BMGs with *T_f_* = 683 K exhibited more densely packed structures. Moreover, the *T_f_* of rejuvenated glassy alloys can also be elevated by pressure annealing [31].

To investigate the role of *T_f_*, four representative alloys – Zr_44_Ti_11_Cu_10_Ni_10_Be_25_, Pd_43_Cu_27_Ni_10_P_20_, Pt_57.5_Cu_14.7_Ni_5.3_P_22.5_, and La_55_Al_25_Ni_20_ – were subjected to thermal cycling [72]. Results revealed that some alloys developed more loosely packed regions and improved fracture toughness, while others became more densely packed and exhibited reduced toughness. Thus, both alloy compositions and *T_f_* are critical in determining whether relaxation or rejuvenation dominates. Importantly, relaxed samples can undergo rejuvenation again depending on their *T_f_* [31, 56, 72, 165]. For example, an alloy relaxed by annealing at Ta (below *T_f_*) can subsequently be rejuvenated at a temperature higher than Ta [56]. Compared with alloys that are already rejuvenated, less rejuvenated or more aged alloys may actually achieve a higher degree of rejuvenation upon subsequent processing [47].

#### 4.1.3 Flash Annealing

**FIGURE 15:**
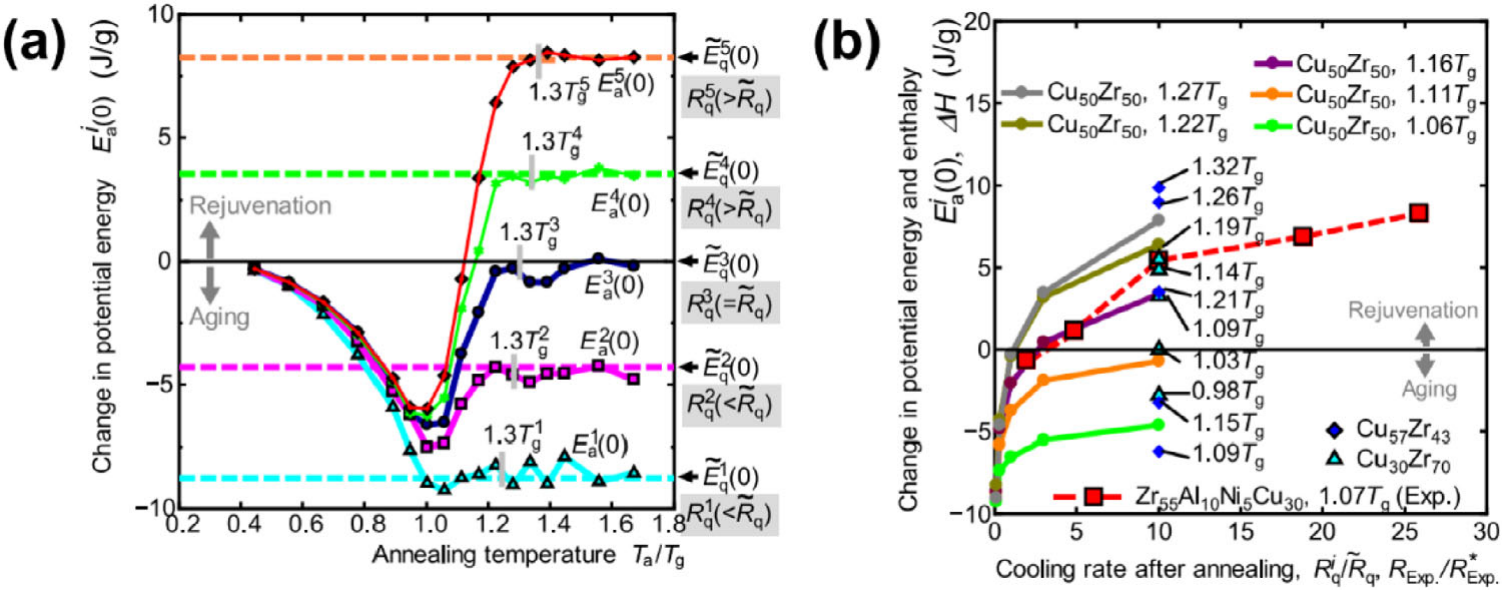
Maps of annealing and subsequent quenching processing [68]: (a) change in the potential energy in the thermal processing (D→G), where $E_{a}^{i}\left(0\right)$ stands for the potential energy under cooling rate of $R_{q}^{i}$. The potential energy under other cooling rate of ${\tilde{R}}_{q}^{i}$ can be found in dashed lines. The grey rectangles stands for 1.3 $T_{g}^{i}$, where $T_{g}^{i}$ is the estimated glass transition temperature in the rapidly quenched alloys with a cooling rate of $\tilde{R_{q}^{i}}$; (b) change in the potential energy via MD and in the enthalpy via experiment. For the cooling rate after annealing, the $R_{q}^{i}$ was used in MD simulations and ${\tilde{R}}_{q}$ was used in the experiment. Reprinted from ref. [68], copyright (2015), with permission from Springer Nature Publishing Group.

Flashing annealing combined with rapid quenching can effectively enhance rejuvenation of glassy alloys [39, 166, 167]. Processing outcomes – whether rejuvenation or aging – are strongly influenced by parameters such as annealing temperature and cooling rate, as illustrated in Figure 15. From Figure 15, several positive effects can be summarized: (i) thermal rejuvenation occurs when the excess potential energy remains positive; (ii) rejuvenated states are observed when the cooling rate exceeds that of as‐cast state; and (iii) in contrast, when the cooling rate is lower than that of initially quenched alloy, aging tends to occur. It should be noted that the final processed states also depend on the initial glassy structures. Specifically, the degree of rejuvenation is governed by two factors: the original state of the alloy and the cooling rate ratio between the initial quenching and subsequent quenching processes. Moreover, the evolution of topological SRO and MRO plays a crucial role in determining whether aging or rejuvenation occurs [166]. A reduction in topological SRO and MRO promotes rejuvenation while suppressing aging.

It has been reported that flash annealing, characterized by an extremely fast heating rate, can drive glass into a high‐energy state (in terms of enthalpy) within less than a millisecond. The subsequent rapid quenching then freezes this elevated enthalpy before the system can reach equilibrium [52]. Experimental results indicate that alloys processed at the shortest flash annealing time *T_FA_* (less than 10^−1^ s) exhibit a comparable magnitude of rejuvenation. In this context, both the heating and quenching rates are critical factors that determine the degree of rejuvenation in glassy alloys. Flash annealing offers several advantages in enhancing the stored energy [52]: (i) high efficiency in energy storage within the processed alloys, (ii) strong capability to recover well‐relaxed glasses, and (iii) extremely rapid heating, providing significant time efficiency.

#### 4.1.4 Thermo‐Mechanical/Pressure Rejuvenation

**FIGURE 16:**
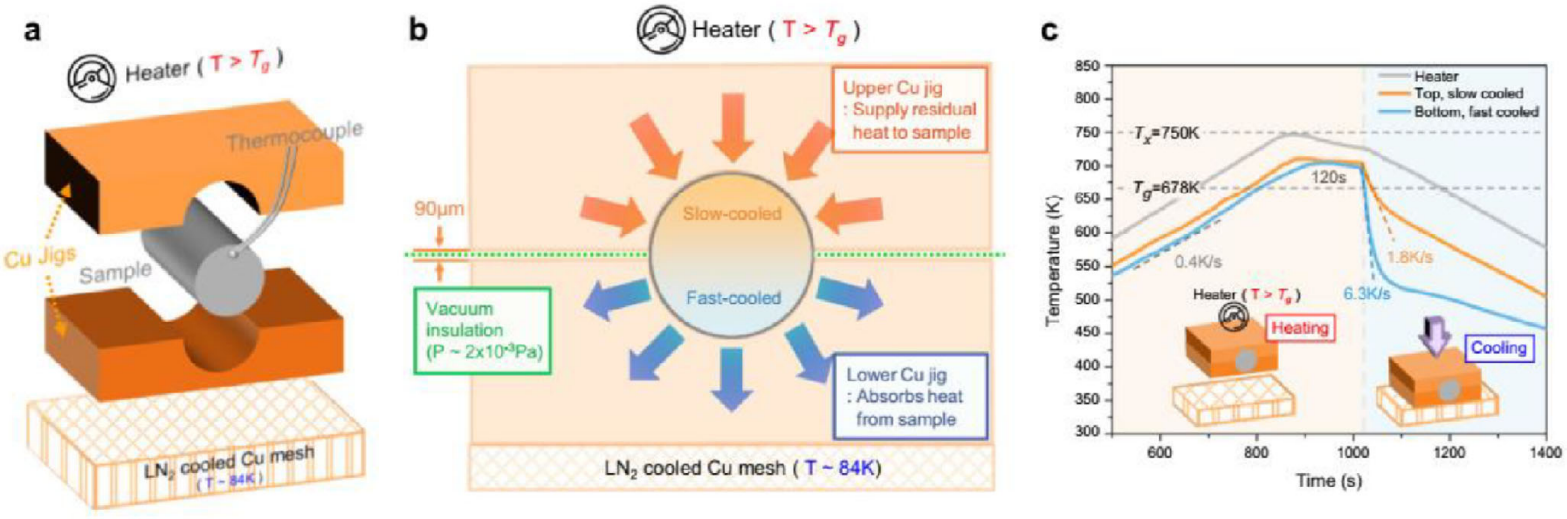
Detailed setup of the heat treatment of the BMG sample [168]: (a) schematic setup of heat treatment in this work; (b) diagram of the heat flow in the glassy alloy during cooling stage in vacuum insulation; (3) curves of temperature‐time for the top and bottom parts of the processed glassy alloy. Reprinted from ref. [168], copyright (2020), with permission from Springer Nature Publishing Group.

Types of thermo‐mechanical or pressure‐induced rejuvenation generally refer to processes that combine temperature with pressure or mechanical effects to rejuvenate glassy alloys [32, 38, 168, 169, 170, 171]. Such thermo‐mechanical/pressure treatments are illustrated in Figure 16. According to Figure 16, the upper and lower levels exhibit distinct cooling rates: the upper level cools at ∼1.8 K/s, while the lower level cools at ∼6.3 K/s. These different cooling rates give rise to gradient structures within glassy alloys. To examine influences of cryogenic temperatures during CTC on rejuvenation behavior, authors subjected three samples to different cryogenic temperatures: 220 K (S3), 180 K (S4), and 140 K (S5). The calculated enthalpies of these processed alloys were 13.4 J/g for S3, 14.1 J/g for S4, and 14.4 J/g for S5 [172], indicating varying degrees of rejuvenation. A schematic of the heat‐treatment setup for glassy alloy, along with the corresponding heat flow and temperature–time curves, is shown in Figure 16. The processed alloys exhibited different thermal release behaviors, resulting in gradient microstructures. Specifically, the bottom surface demonstrated a higher thermal release rate compared to the top surface [168].

Greer and Sun further compared the relaxation enthalpy of three treated alloys: as‐cast, laterally ESL‐loaded at 298 K, and laterally ESL‐loaded at 77 K. Their results showed that the alloys treated at 77 K possessed the highest enthalpy [173]. MD simulations suggested that lateral ESL compression increases the fraction of loosely packed clusters while decreasing the fraction of densely arranged clusters. Low‐temperature treatments, such as liquid‐nitrogen processing (77 K), were found to further amplify these structural changes at the atomic‐cluster level. Moreover, thermal‐mechanical creep (or anelastic creep strain) raises the *T_f_* and thereby increases the density of STZs [174]. Importantly, rejuvenation induced by thermal‐mechanical creep not only occurs locally but can also extend to the first atomic shell.

### 4.2 Thermal Rejuvenation: Atomic Changes and Stages

#### 4.2.1 Atomic Changes of Thermal Rejuvenation

**FIGURE 17:**
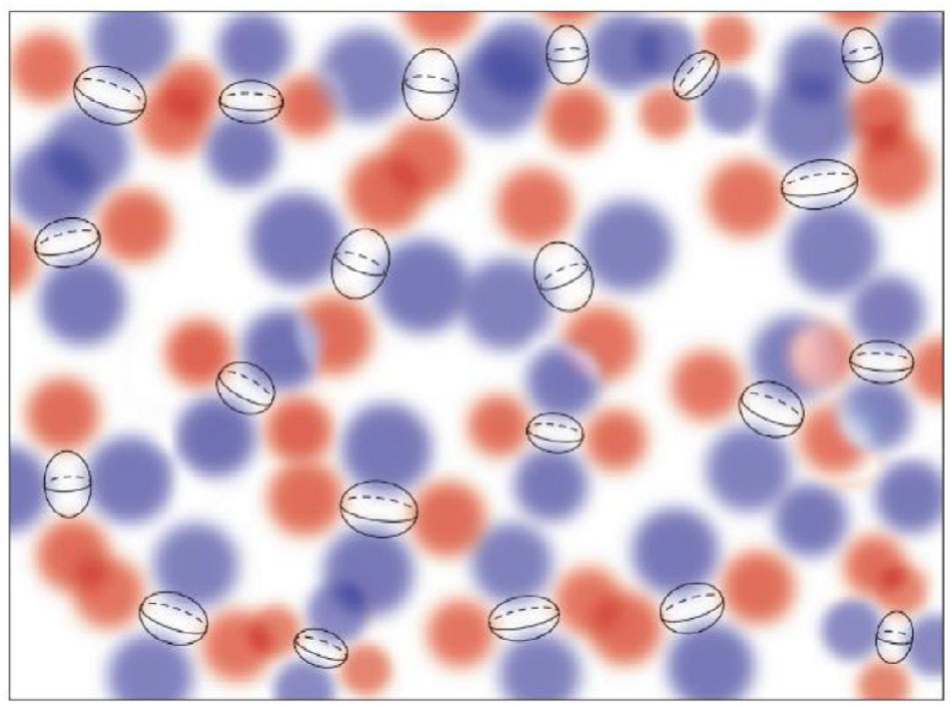
Heterogeneous thermal expansion coefficient [22]. The thermal expansion coefficient (represented by ellipsoids) of a glass varies locally due to the heterogeneous nature of the structure. When the temperature is changed, these local variations in thermal expansion cause internal stresses (red and blue regions indicate compression and tension, respectively) to develop in the glass. Reprinted from ref. [22], copyright (2015), with permission from Springer Nature Publishing Group.

For thermal processing, the local atomic structure is closely correlated with the processing temperatures [22, 67], and might be correlated with thermal expansion coefficient in glassy alloys, shown in Figure 17. Structural changes in interatomic distances may be origins of rejuvenation in glassy alloys during cryogenic treatment. In particular, heterogeneity in the local coefficient of thermal expansion or non‐affine atomic displacements plays a critical role in the overall volume change [46, 67, 147]. For example, under temperature fluctuations, a decrease in temperature causes inhomogeneous shrinkage in glassy alloys at atomic scale, especially among atoms in higher coordination shells (i.e., MRO) [41]. In other words, non‐uniform shrinkage has been observed across different atomic shells. Moreover, lowering the temperature increases the interatomic distance in the first nearest‐neighbor shell, while decreasing it in other shells [41]. This difference in interatomic distance between the first shell and subsequent shells is referred to as inhomogeneity.

Further evidence comes from studies on Zr_55_Cu_30_Al_10_Ni_5_ glassy alloy, which suggest that rejuvenation may arise from atomic‐level core–shell heterogeneous microstructures [91]. Minor alloying elements also play a critical role in rejuvenation and relaxation behavior [175]. For instance, increasing the Ta content in Zr‐Cu‐Al‐Ni alloys has been shown to raise the energy state of the system. Atomic clusters likewise contribute to rejuvenation [47]. The most significant difference between aged and rejuvenated alloys is a ∼4% variation in the <0,0,12,0> content of Cu‐centered clusters [47].

In one MD study, Cu–Zr glassy alloys underwent two annealing treatments: a moderate‐temperature annealing at 900 K and a high‐temperature annealing at 1100 K. The degree of rejuvenation observed after the second, higher‐temperature annealing was greater than that after the 900 K treatment. One possible explanation is the modification of icosahedral clusters: specifically, more icosahedral clusters were transformed into other types of clusters with lower local fivefold symmetry. This structural change reduces the SRO of the Cu–Zr system and increases the potential energy, which is primarily associated with the evolution of Cu‐centered clusters. In general, higher potential energy correlates with a decrease in SROs [47].

A negative example illustrating the relationship between heterogeneity and clusters is that a reduction in the population of <0,0,12,0> clusters results in an increased inhomogeneity or degradation of SROs in glassy alloys. The origins of rejuvenation have been further investigated in other studies [165, 172]. It has been proposed that rejuvenation may arise not from atomic ordering, but rather from changes in packing density [165] or the recovery of loosely packed structures [172]. For instance, Cu–Zr–Al BMGs subjected to recovery annealing and cold thermal cycling treatments exhibited more heterogeneous atomic structures, characterized by a lower fraction of icosahedral and defective icosahedral clusters, but a higher fraction of bcc‐ and FCC/HCP‐type clusters [54].

Meng et al. [165] further showed that the mechanism of rejuvenation below the *T_g_* differs fundamentally from that above *T_g_*. At the rejuvenation stage, the destruction of atomic structures may be linked to local bond‐breaking events through the *β*‐transition. Similar transitions have been reported in other systems, such as relaxation‐to‐rejuvenation in Fe_80_P_20_ and rejuvenation‐to‐relaxation in Ni_60_Nb_40_ [46]. These processes may be associated with a first‐order phase transition [108], although this interpretation remains under debate. Another study suggested that during CTC, rejuvenation could be reactivated or “recalled” from the liquid origin due to the liquid memory effects of glassy alloys [165].

#### 4.2.2 Stages of Thermal Rejuvenation

According to literature [40], the thermal rejuvenation process induced by the CTC can be divided into three stages: Stage I (rejuvenation) – characterized by an increase in enthalpy, Stage II (relaxation) – involving a decrease in enthalpy, and Stage III (steady state) – where the retained enthalpy approaches that of the as‐cast alloy. In Stage I, stored energy accumulates in the soft regions, leading to an increase in retained elastic distortion within the matrix. In Stage II, the enhanced mobility in the soft domains amplifies the elastic push‐back effect, which in turn accelerates the progressive collapse of CTC‐induced shears. In Stage III, shear‐related or stored energy is no longer accumulated owing to the sustained atomic mobility. Furthermore, rejuvenation of annealed alloys has been proposed as another promising route to enhance the ductility of glassy alloys [56]. However, the rejuvenation and annealing parameters must be carefully optimized.

In order to have a comprehensive understanding of rejuvenated states and enthalpy (or processing routes), a series of processing parameters such as annealing numbers, temperatures, etc., on energy states and properties will be careful examined. The memory effect is one important feature of glassy alloys and links two states – liquid state and solid state for amorphous alloys [24, 28, 165, 176, 177]. This memory effect makes the quenched or relaxed glassy alloys back to their liquid state via some processing methods such as intermediate temperature annealing. To be specific, at atomic perspective, some atomic structures can be rejuvenated to its original states while some atomic clusters cannot be rejuvenated. From kinetics perspective regarding the stability of rejuvenation, repeated thermal cycling can tuning the value of stored energy (enthalpy of rejuvenated glassy alloys) [176]. Wang et al. [176] defined the rejuvenation ability to describe the relationship between relaxed and rejuvenation states. Results indicated that the rejuvenation ability remains the same at initial three cycles and decreases with an increase in thermal cycling. One important aim of rejuvenation is to enhance the mechanical properties. These critical findings give a clear clue that one optimal processing thermal cycling via memory effect should be existed for any specific glassy composition. In future, optimizing mechanical properties of glassy alloys via thermal cycling is another important routes. After rejuvenation, the processed glassy alloys start to relax depending on temperature and stress [177]. The creep of glassy alloy is one important feature of transition from rejuvenation to relaxation. Authors examined the relaxation evolution via the thermos‐mechanical coupling experiments and found that during creep process, some physical phenomena like the *β*‐ relaxation and configurational entropy can be changed. In this sense, methods to examine these physical features via coupling of thermal‐mechanical processing can be applied to stabilize the rejuvenated glassy alloys. Specific methods corresponding to a given alloy composition should be pinpointed in future.

### 4.3 Alloy Composition and Processing Parameters

The degree of rejuvenation during thermal processing is influenced by multiple factors, including the original state of glassy alloys (e.g., as‐cast condition or alloy composition) as well as the processing parameters. The effects of these factors are complex and difficult to fully unravel, yet remain worthwhile to investigate. The original state of a glassy alloys can be tailored by adjusting the quenching rate, alloy composition, or melting power, whereas processing parameters, such as the number of thermal cycles, can also be employed to tune its structural state.

#### 4.3.1 Alloy Composition

The relaxation enthalpy is an important characteristic of various glassy alloys [23, 40, 164]. Its optimization strongly depends on the alloy composition, as the number of CTC plays a critical role. For example, 45 cycles of CTC yield the highest relaxation enthalpy of 333 J/mol in the Ti_40_Zr_10_Cu_34_Pd_14_Sn_2_ alloy, while 100 cycles lead to a maximum enthalpy of 208 J/mol in the Pd_43_Cu_27_Ni_10_P_20_ alloy. In comparison, the maximum enthalpy values of 349 and 296 J/mol are achieved after 35 cycles in Pt‐based alloys and 30 cycles in Zr‐based alloys, respectively [40]. For La_55_Ni_20_Al_25_ glassy ribbons and La_55_Ni_10_Al_35_ amorphous rods, distinct enthalpy behaviors are observed [23]: the former reaches its maximum enthalpy after 10 cycles, whereas the latter requires 15 cycles. Wang et al. [165] further investigated Δ*H_rel_* as a function of cycle number (0–200). For sample S1 (ribbon fabricated at 1000 r/min), the relaxation enthalpy increases and then decreases, reaching its maximum at 100 cycles – about 36.4% higher than the as‐cast state. For sample S2 (fabricated at 2000 r/min), the maximum occurs at 50 cycles, corresponding to a 17.7% increase. However, for sample S3 (fabricated at 3000 r/min), the enthalpy continuously decreases with increasing cycles, indicating dominant relaxation.

For Zr_59.5_Nb_4.8_Cu_14.4_Ni_11.6_Al_9.7_ alloys, optimized microstructures and mechanical properties can be achieved through controlled thermal cycling [67]. Wang et al. [47] demonstrated that the modification of icosahedral SROs during repeated thermal cycling is highly sensitive to the Cu content. A transition between aging/relaxation and rejuvenation can occur, governed by the relative proportions of Cu‐centered and Zr‐centered polyhedra within STZs [178]. During thermal‐pressure rejuvenation, increasing the Cu content promotes the formation of <0,0,12,0> polyhedra, thereby enhancing yield stress [38]. At early stages of thermal cycling, the loosely packed Nb‐centered cores tend to relax into a low‐energy state, whereas the densely packed Ni‐centered shells are prone to rejuvenation into a high‐energy state [147]. Overall, influences of alloy composition are complex and multifaceted, and further studies are required to clarify the underlying mechanisms of rejuvenation.

#### 4.3.2 Processing Parameters

Although alloy composition is a critical factor influencing rejuvenation, other parameters, such as processing conditions, also play an important role in modifying atomic microstructures [38, 39, 40, 50, 53, 55, 178]. Both processing time and the number of thermal cycles exert significant effects on microstructural evolution and mechanical properties. For instance, different holding times at both ambient and cryogenic temperatures during CTC have been applied to Cu–Zr binary glassy alloys [55]. The main findings can be summarized as follows: (*i*) both aged and rejuvenated states can be obtained in samples subjected to different holding times, as confirmed by experiments and simulations; (*ii*) shorter holding times generally promote rejuvenation, whereas longer holding times tend to induce structural aging. Simulation results further support this trend: rejuvenation was observed at holding times of 2 and 5 ps, while aging or structural relaxation occurred at 10 and 200 ps [55].

**FIGURE 18:**
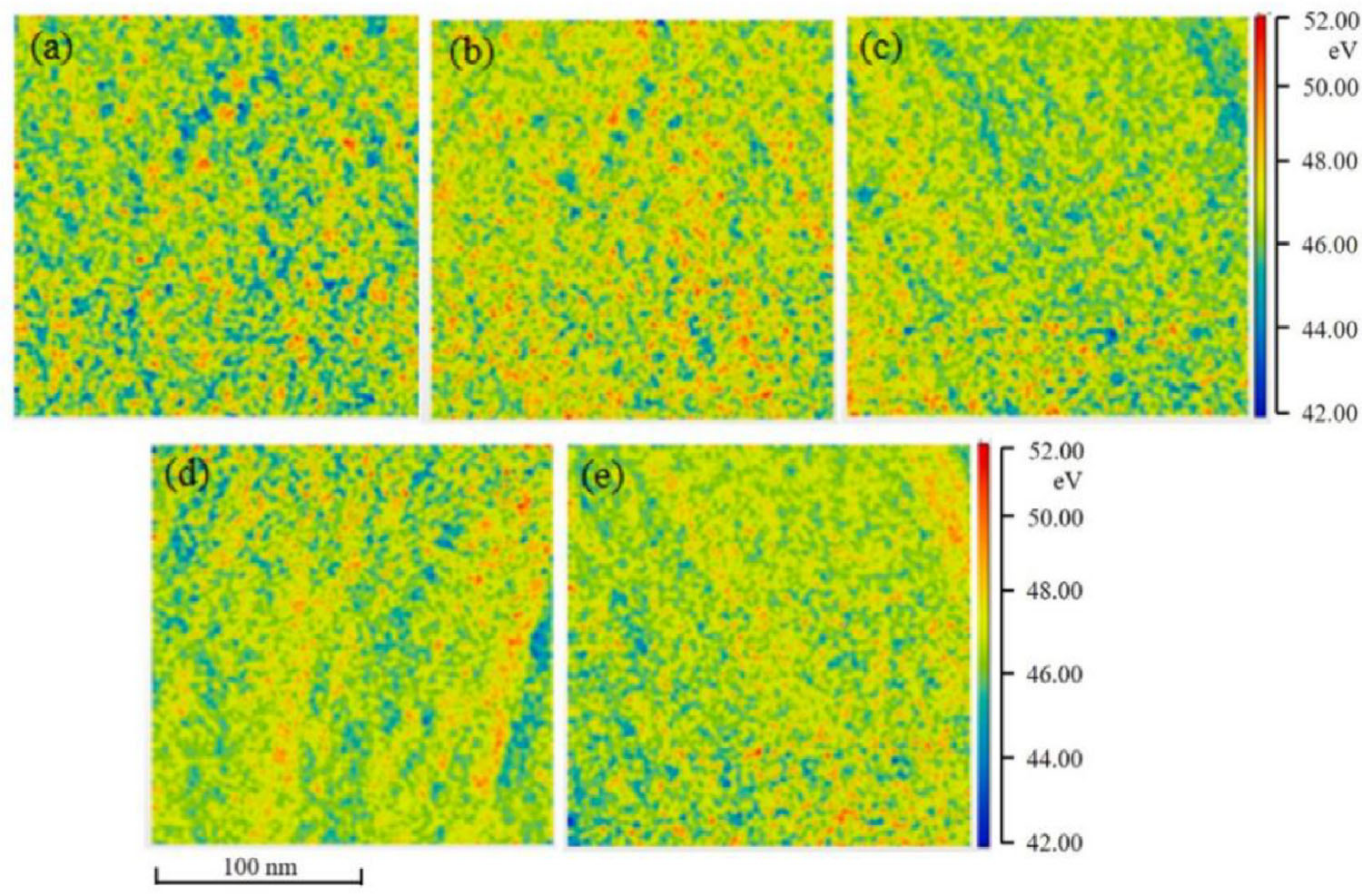
Images of AM‐AFM energy dissipation for various structures of (a) as‐cast; (b) CTC 293K after 15 cycles; (c) CTC 293K after 200 cycles; (d) CTC 373K after 15 cycles; (e) CTC 373K after 200 cycles [50]. Reprinted from ref. [50], copyright (2022), with permission from Elsevier BV Publishing Group.

It has been reported that energy dissipation in loosely packed regions occurs much faster than in densely packed domains. For example, in CTC at 293 K, the volume fraction of loosely packed regions increased after 15 DCT cycles, whereas it decreased after cycling at 373 K [50]. Similarly, the formation of loosely packed regions with increased average interatomic spacing has been identified in CTC at 513 and 533 K [51, 53]. Another structural indicator of rejuvenation is the formation of CLO structures [39, 51, 101]. The volume fraction of CLO structures increases from ∼15% in the as‐quenched alloy to ∼25% after CTC at 513 K. Likewise, the fraction of CLO regions increases from ∼23.5% in the as‐cast state to ∼25% at 673 K; however, further increases in temperature to 773 and 813 K lead to a gradual decrease in CLO fraction (to ∼19.5 and ∼18%, respectively) [39]. The energy dissipation behaviors of different structures as a function of processing temperature and cycling number are illustrated in Figure 18.

The annealing temperature exerts distinct effects on aging and rejuvenation states of metallic glasses [178]. Specifically, during prolonged isothermal holding, annealing at 900 K or lower accelerates structural aging, whereas annealing at 1000 K or higher promotes rejuvenation. At temperatures above 1000 K, the degree of rejuvenation becomes more pronounced. MD simulations on Cu–Zr alloys confirm these trends and are consistent with experimental observations. For instance, in flash annealing experiments [39], heating the glassy alloys to 673 K leads to a slight increase in yield strength, but catastrophic failure occurs after only limited plastic deformation. With further increases in temperature to 773 and 873 K, the yield strength decreases while plastic strain progressively increases. The correlation between annealing temperature and atomic cluster evolution can be described as follows: when the annealing temperature is below 1000 K, the fractions of Cu‐centered <0,0,12,0> and Zr‐centered <0,1,10,4> clusters increase with longer isothermal holding. In contrast, at annealing temperatures above 1000 K, the populations of these clusters decrease with prolonged annealing [178].

Three processing parameters – final cooling rate, isothermal time, and annealing temperature – have been employed to rejuvenate glassy alloys [73, 178]. Six samples are compared: M1, M3, and M5 in the aged state, and M2, M4, and M6 in the rejuvenated state. For instance, comparing M1 and M2, annealing at 900 and 1000 K leads to distinct structural states. By contrast, comparisons such as M1 with M4 and M3 with M2 reveal that higher annealing temperatures combined with relatively faster final cooling rates promote rejuvenation. In another case, M1 versus M5, and M2 versus M6 demonstrate that variations in isothermal holding time result in different extents of aging and rejuvenation. Generally, a slower final cooling rate suppresses rejuvenation, leading to formation of numerous nano‐clusters [73]. Specifically, increasing the final cooling rate decreases populations of <0,0,12,0> and <0,1,10,4> clusters, whereas higher annealing temperatures enhance their presence [178].

**FIGURE 19:**
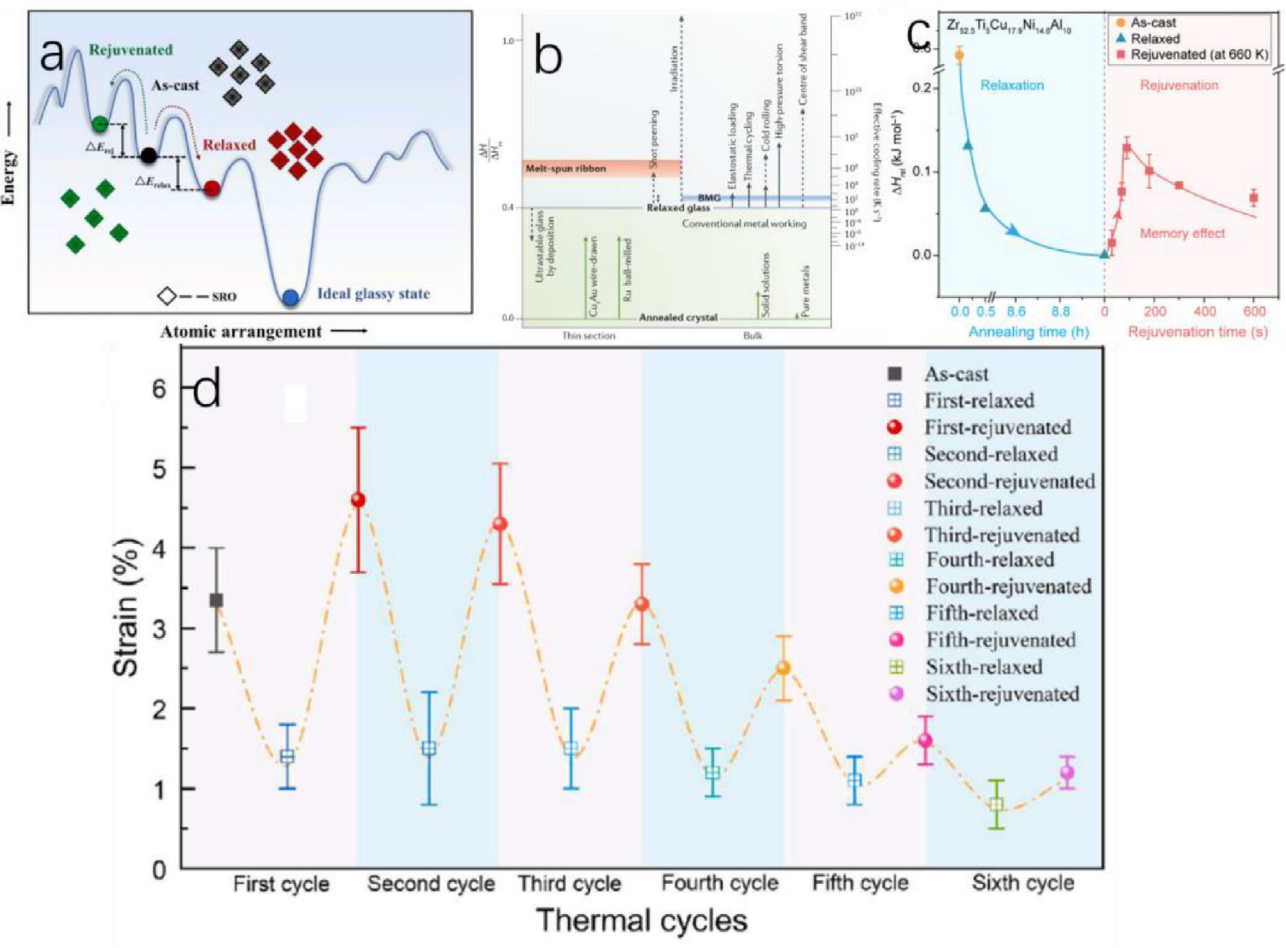
(a) Relative energy states for rejuvenated, as‐cast and relaxed glassy alloys and ideal glassy states [39]; (b) Various energy states for processing methods for glassy alloys [2]; (c) For thermal annealing, the relationship between memory effects, processing parameters and energy states [56]; (d) Decoupling between mechanical strain and enthalpy for repeated thermal annealing [176]. Reprinted from ref. [2, 39, 56, 176], copyright (2016, 2021, 2022, 2026), with permission from Elsevier Editora Ltda Publishing Group.

Different pressures exert distinct influences on degrees of rejuvenation [38]. For instance, when processed alloys were subjected to pressures ranging from 0 to 10 GPa, localized deformation and structural changes were observed. As the pressure increased from 10 to 30 GPa, however, the degree of rejuvenation gradually decreased. This suggests that an optimal level of structural disorder occurs within the 10–30 GPa range. At the same time, variations in STZs, SBs, and Poisson's ratio were also observed with increasing pressure from 0 to 30 GPa. In order to have an understanding of relationship between rejuvenation methods and energy states, a few critical correlations have been given, shown in Figure 19. Generally, the rejuvenated glassy alloys have a higher energy state than that of as‐cast and relaxed ones, shown in Figure 19a, and different processing routes usually result in different stored energy in rejuvenated glassy alloys, shown in Figure 19b. Thermal processing of annealed alloys at relative higher temperature is another unique example, shown in Figure 19c, which tells different pictures of decoupling relationship between mechanical strain and enthalpy, shown in Figure 19d. According to studies [37, 56, 176, 177], the aged/relaxed glassy alloys can be rejuvenated via the thermal annealing at relatively higher temperatures. All in all, rejuvenation via multiple processing routes might result in some interesting and different outcomes.

### 4.4 Microstructures

Modified microstructures of glassy alloys produced through thermal rejuvenation methods generally exhibit two distinct states: aged and rejuvenated [47, 175, 178]. Aged glassy alloys typically show reduced ductile plasticity, whereas their rejuvenated counterparts display enhanced plastic strain. To better characterize these modified structures, several critical features will be discussed. These features can be broadly categorized into two groups: macroscopic features (also referred to as physical traits) and microscopic features (also referred to as atomic traits).

#### 4.4.1 STZs, β Relaxation, Hardness, and Elastic Modulus, Defect Density and Enthalpy

**FIGURE 20:**
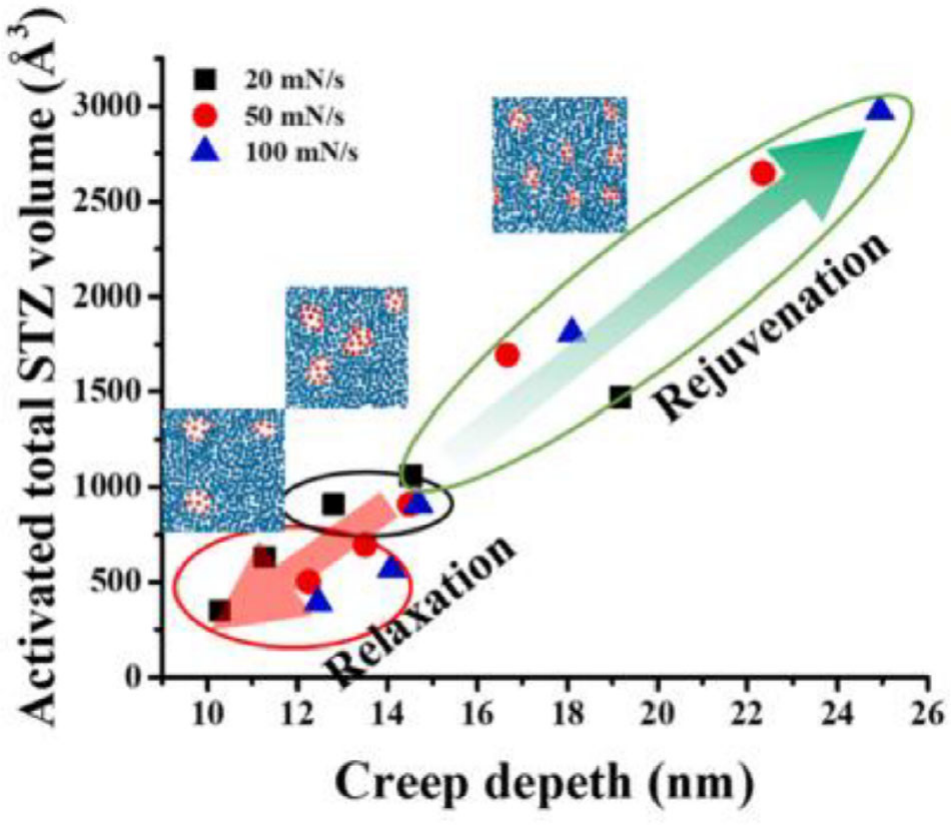
Schematic the mechanism of relaxation and rejuvenation of MG via DCT [49]. Reprinted from ref. [49], copyright (2022), with permission from Elsevier BV Publishing Group.

Rejuvenation of alloys is closely correlated with activation of STZs volume, as shown in Figure 20. The volume fraction of STZs in both as‐quenched and structurally relaxed glassy alloys decreases with an increasing number of DCT [49]. A study on La_60_Ni_15_Al_25_ revealed that with increasing numbers of CTC, rejuvenation initially occurs, followed by structural relaxation after a certain number of cycles [50]. Specifically, when the number of CTC cycles is fewer than 200, alloys processed under CTC at 293 K exhibit a higher energy state than the as‐cast state, whereas alloys processed under CTC at 373 K exhibit a lower energy state compared with the as‐cast state. This transition from rejuvenation to relaxation during CTC may be associated with variations in the volume fraction of STZs. However, when the number of CTC cycles exceeds 200, the energy states of alloys processed under both CTC at 293 and 373 K become lower than that of the as‐cast state. Potential STZs, regarded as clusters of atoms [46], have been reported to correlate with both rejuvenation and *β*‐relaxation process [108]. The number of potential STZs decreases in structurally relaxed glassy alloys, and in some cases, the reduction cannot be fully reversed through cryogenic cycling [46].

The plasticity or deformation of glassy alloys is characterized by various formation and propagation of SBs. The SBs is macroscopic features of STZ activation during deformation. The relationship between deformation and heterogeneous structures has been comprehensively investigated [3, 18, 19]. For glassy alloys, the spatial heterogeneity at nanoscale and its density fluctuation are closely correlated with the local atomic structure variations from icosahedron‐like to tetragonal crystal‐like order [3, 57]. In this regard, the deformation of glassy alloys should be correlated with variations of atomic clusters. The STZ activation is also correlated with various atomic clusters. Studies on structures of glassy alloys [16, 17] indicated that variations of atomic clusters consist of not only the clusters connectivity but also many factors such as length, types, arrangements, etc., of atomic clusters. All these defined atomic clusters form various stereo‐chemical structures, names the spatial atomic structures. Any dimension of spatially structured clusters (SROs and MROs) exerts different roles in deformation. Although it is relatively difficult to decode the relationship between STZs activation and atomic cluster connectivity, some examples are still necessary. The rejuvenation enhances the connectivity of Zr‐Zr pairs while breaks the Zr‐Cu pairs [39]. To be specific, for glassy alloy of Zr_61_Cu_25_Al_12_Ti_2_, the rejuvenated state has an increased volume fraction of the 4‐atom and 2‐atom cluster connections while aged state has a decreased density of the 3‐atom clusters. Feng et al. [113] also found that a decrease in 3‐atom connections in MROs improves rejuvenation while an increase in 2‐atom and 4‐atom connections in MROs enhances local plastic units. Actually, the STZs activation and corresponding deformation are sensitive to various atomic clusters, but this dependence is relatively complicated.

For alloys subjected to treatments in liquid nitrogen and high‐temperature silicone oil at different temperatures, the hardness and elastic modulus exhibit similar trends [51]. The CTC 473 process leads to a reduction in both hardness and elastic modulus compared with the as‐cast alloys. An increase in elastic modulus may result from the enhancement of icosahedral SROs [68]. A decrease in hardness is observed for the t25 sample, whereas gradient structures with varying hardness appear in the t70 and t150 samples. Moreover, the volume fraction of loosely packed regions with lower elastic stiffness increases with the number of thermal cycles [8]. Typical examples of hardness evolution are as follows. For Fe_80_P_20_, hardness initially increases with cycling, reaching a maximum of ∼8.45 GPa at 10 cycles, before gradually decreasing to ∼7.78 GPa at 40 cycles [147]. With further cycling, fluctuations in hardness are observed. In contrast, for Ni_60_Nb_40_, the hardness decreases from ∼7.8 GPa in the as‐cast state to ∼6.8 GPa after 10 cycles, followed by fluctuations during subsequent cycling. When the number of cycles reaches 100, hardness increases again to ∼7.25 GPa. Wang et al. [55] reported that the hardness of short‐time processed alloys is higher than that of as‐quenched alloys, while that of long‐time processed alloys surpasses the as‐cast state.

**FIGURE 21:**
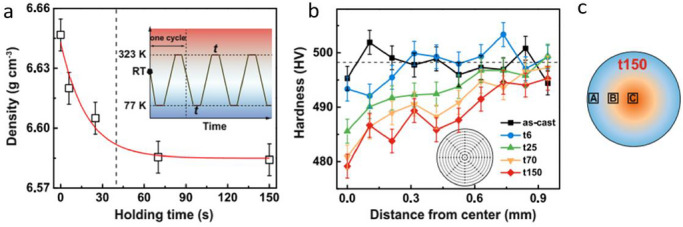
For glassy alloys processed by CTC, density and hardness are changed with processing parameters [8]: (a) values of measured density as a function of holding time for the processed amorphous alloys. The inset is the CTC from a high temperature of 323 K to temperature of 77 K together with the waiting time of t; (b) variations of measured average hardness along the distance from the center and the inset is the hardness measuring method along various circles; (c) the measured sites for density and hardness. Reprinted from ref. [8], copyright (2022), with permission from Springer Nature Publishing Group.

Defect density and enthalpy are key macroscopic indicators of rejuvenated glassy alloys [8], shown in Figure 21. According to ref. [8], the density of processed alloys decreases with increasing holding time, shown in Figure 21a, while evolution of hardness in rejuvenated glassy alloys also functions with distance between tested points and center regions, shown in Figure 21b,c. Moreover, the CTC promotes the accumulation of structural defects, and the defect density rises with increasing cycle numbers [179]. The maximum defect volume fraction is observed at around 60 cycles. Consistently, a reduction in alloy density is reported with increasing cycle number [150]. For Fe_80_P_20_ [147], the relaxation enthalpy initially decreases during the first 10 cycles, followed by a subsequent increase upon further cycling. In contrast, Ni_60_Nb_40_ exhibits an opposite trend, where relaxation enthalpy first increases within the initial 10 cycles and then decreases with additional cycles. Importantly, whether the relaxation enthalpy is higher or lower than that of the as‐cast state is not a decisive indicator of the degree of rejuvenation [56]. Related studies on the correlation between relaxation enthalpy and plasticity have also been reported by Wang et al. [55]. For thermal rejuvenation methods, related macroscopic features such as hardness, elastic modulus and relaxation enthalpy as a function of processing parameters can also be found in Table 3.

**TABLE 3:** Hardness and elastic modulus as well as relaxation enthalpy as a function of processing parameters.

Alloy composition	Processing parameters	Hardness (HV)	Hardness (GPA)	Elastic modulus (GPa)	Relaxation enthalpy (J/mol)	Relaxation enthalpy(J/g)	References
Zr_61_Cu_25_Al_12_Ti_2_	As‐cast			84.2± 0.8	99± 23		[39]
Zr_61_Cu_25_Al_12_Ti_2_	Heating to 673 K			90.2± 1.8	76± 14		[39]
Zr_61_Cu_25_Al_12_Ti_2_	Heating to 773 K			83.6± 0.9	151± 19		[39]
Zr_61_Cu_25_Al_12_Ti_2_	Heating to 813 K			79.2± 0.7	245± 14		[39]
Pd_43_Cu_27_Ni_10_P_20_	As‐cast				377		[40]
Pd_43_Cu_27_Ni_10_P_20_	100 cycling				585		[40]
Pd_43_Cu_27_Ni_10_P_20_	200 cycling				～ 377		[40]
Zr_59.5_Nb_4.8_Cu_14.4_Ni_11.6_Al_9.7_	As‐cast					11.4	[67]
Zr_59.5_Nb_4.8_Cu_14.4_Ni_11.6_Al_9.7_	10 number of DCT					12.15	[67]
Zr_59.5_Nb_4.8_Cu_14.4_Ni_11.6_Al_9.7_	20 number of DCT					13.2	[67]
Zr_59.5_Nb_4.8_Cu_14.4_Ni_11.6_Al_9.7_	30 number of DCT					12.3	[67]
Zr_59.5_Nb_4.8_Cu_14.4_Ni_11.6_Al_9.7_	40 number of DCT					11.55	[67]
Zr_47.5_Cu_47.5_Al_5_	0.8Tg‐R30		7.50± 0.12			0.473	[49]
Zr_47.5_Cu_47.5_Al_5_	0.8Tg		7.95± 0.11			0.692	[49]
Zr_47.5_Cu_47.5_Al_5_	R30		7.26± 0.11			3.68	[49]
Zr_47.5_Cu_47.5_Al_5_	R10		7.53± 0.24			1.03	[49]
Zr_47.5_Cu_47.5_Al_5_	As‐cast		8.14± 0.07			1.42	[49]
La_60_Ni_15_Al_25_	As‐cast		2.64		187		[50]
La_60_Ni_15_Al_25_	CTC 293 K		2.54				[50]
La_60_Ni_15_Al_25_	CTC 293 K: 5times				212		[50]
La_60_Ni_15_Al_25_	CTC 293 K: 15times				230		[50]
La_60_Ni_15_Al_25_	CTC 293 K: 200times				147		[50]
La_60_Ni_15_Al_25_	CTC 323 K		2.54				[50]
La_60_Ni_15_Al_25_	CTC 373 K		2.68				[50]
La_60_Ni_15_Al_25_	CTC 373 K: 5times				224		[50]
La_60_Ni_15_Al_25_	CTC 373 K: 15times				175		[50]
La_60_Ni_15_Al_25_	CTC 373 K: 200times				134		[50]
Ba_96_Nb_4_	As‐cast		9.60	142.25	379		[51]
Ba_96_Nb_4_	CTC 473 K		9.04	140.58	643		[51]
Ba_96_Nb_4_	CTC 393 K		8.99	140.02			[51]
Ba_96_Nb_4_	CTC 433 K				467		[53]
Ba_96_Nb_4_	CTC 513 K				663		[53]
Ba_96_Nb_4_	CTC 533 K				705		[53]
Ba_96_Nb_4_	CTC 563 K				410		[53]
Ti_20_Zr_20_Hf_20_Be_20_Cu_20_	As‐cast					4.376	[150]
Ti_20_Zr_20_Hf_20_Be_20_Cu_20_	DCT 5					5.802	[150]
Ti_20_Zr_20_Hf_20_Be_20_Cu_20_	DCT 10					5.964	[150]
Ti_20_Zr_20_Hf_20_Be_20_Cu_20_	DCT 15					5.237	[150]
Ti_20_Zr_20_Hf_20_Be_20_Cu_20_	DCT 20					5.279	[150]
Zr_46_Cu_46_Al_8_	As‐cast					5.451	[179]
Zr_46_Cu_46_Al_8_	10 numbers of cycling					9.356	[179]
Zr_46_Cu_46_Al_8_	20 numbers of cycling					8.053	[179]
Zr_46_Cu_46_Al_8_	60 numbers of cycling					7.033	[179]
Fe_80_P_20_	As‐cast		7.60				[147]
Fe_80_P_20_	10 cycling of CTC		8.45				[147]
Fe_80_P_20_	40 cycling of CTC		7.78				[147]
Ni_60_Nb_40_	As‐cast		7.8				[147]
Ni_60_Nb_40_	10 cycling of CTC		6.8				[147]
Ni_60_Nb_40_	100 cycling of CTC		7.25				[147]
Zr_55_Cu_30_Al_10_Ni_5_	As‐cast		4.42± 0.05	115		12.7	[91]
Zr_55_Cu_30_Al_10_Ni_5_	DCT 30		4.30± 0.08	106		14.6	[91]
(Zr_0.55_Cu_0.3_Ni_0.05_Al_0.1_)_99_Ta_1_	As‐cast		4.46± 0.04	116		13.6	[175]
(Zr_0.55_Cu_0.3_Ni_0.05_Al_0.1_)_99_Ta_1_	DCT30		4.32± 0.02	106		15.7	[175]
(Zr_0.55_Cu_0.3_Ni_0.05_Al_0.1_)_97_Ta_3_	As‐cast		4.53± 0.04	117		9.2	[175]
(Zr_0.55_Cu_0.3_Ni_0.05_Al_0.1_)_97_Ta_3_	DCT30		4.35± 0.02	106		10.8	[175]
(Zr_0.55_Cu_0.3_Ni_0.05_Al_0.1_)_95_Ta_5_	As‐cast		4.63± 0.05	119		10.8	[175]
(Zr_0.55_Cu_0.3_Ni_0.05_Al_0.1_)_95_Ta_5_	DCT30		4.40± 0.04	108		12.8	[175]
Zr_46_Cu_46_Al_8_	As‐cast	683± 31		107± 5		10.5	[54]
Zr_46_Cu_46_Al_8_	Relax	719± 33		113± 7		0	[54]
Zr_46_Cu_46_Al_8_	Rej300	692± 63		110± 5		2	[54]
Zr_46_Cu_46_Al_8_	DCT30	656± 30		105± 4		12.1	[54]
Zr_52.5_Cu_17.9_Ni_14.6_Al_10_Ti_5_	As‐cast	511± 1.6			852± 25		[56]
Zr_52.5_Cu_17.9_Ni_14.6_Al_10_Ti_5_	Relaxed at 570 K for 9h	526± 1.6			0		[56]
Zr_52.5_Cu_17.9_Ni_14.6_Al_10_Ti_5_	Heating to 640 K	523.8± 1.1			19.3± 21		[56]
Zr_52.5_Cu_17.9_Ni_14.6_Al_10_Ti_5_	Heating to 650 K	521.4± 1.7			76.8± 28		[56]
Zr_52.5_Cu_17.9_Ni_14.6_Al_10_Ti_5_	Heating to 655 K	519.5± 1.3			95.7± 36		[56]
Zr_52.5_Cu_17.9_Ni_14.6_Al_10_Ti_5_	Heating to 660 K	517.7± 1.5			129± 13		[56]
Zr_52.5_Cu_17.9_Ni_14.6_Al_10_Ti_5_	Heating to 661 K	518.1± 2.0			75± 6		[56]
Zr_52.5_Cu_17.9_Ni_14.6_Al_10_Ti_5_	Heating to 662 K	518.4± 2.0			102± 31		[56]
Zr_52.5_Cu_17.9_Ni_14.6_Al_10_Ti_5_	Heating to 663 K	518.4± 2.6			90± 14		[56]
Zr_52.5_Cu_17.9_Ni_14.6_Al_10_Ti_5_	Heating to 664 K	520.2± 2.1			83± 20		[56]
Zr_52.5_Cu_17.9_Ni_14.6_Al_10_Ti_5_	Heating to 665 K	520± 2.4			60± 21		[56]
Zr_52.5_Cu_17.9_Ni_14.6_Al_10_Ti_5_	Heating to 666 K	518.7± 1.6			95± 36		[56]
Zr_52.5_Cu_17.9_Ni_14.6_Al_10_Ti_5_	Heating to 670 K				18.5± 11		[56]
Zr_52.5_Cu_17.9_Ni_14.6_Al_10_Ti_5_	Heating to 680 K	515.7± 0.8			19.6± 29		[56]
Zr_52.5_Cu_17.9_Ni_14.6_Al_10_Ti_5_	Heating to 720 K	511± 1.5			535± 8		[56]
Zr_47.5_Cu_47.5_Al_5_	As‐cast			101		15.9	[73]
Zr_47.5_Cu_47.5_Al_5_	Relaxed			104			[73]
Zr_47.5_Cu_47.5_Al_5_	Rej700 (700 K/min)			102± 2		7.5	[73]
Zr_47.5_Cu_47.5_Al_5_	Rej300 (300 K/min)			102± 3		4.9	[73]
Zr_47.5_Cu_47.5_Al_5_	Rej100 (100 K/min)			107± 3		2.1	[73]

#### 4.4.2 Atomic Clusters and Packing Density

Atomic clusters and packing density are key microscopic features of glassy alloys subjected to thermal processing. To better understand their evolution, several examples are discussed. Thermal cycling can reduce the fraction of 3‐atom connections (associated with ordered or solid‐like atomic packing) while increasing the fractions of 2‐atom and 4‐atom connections (characteristic of liquid‐like packing) [48]. In Cu‐Zr glassy alloys with high Cu content, highly centrosymmetric <0,0,12,0> and <0,0,12,4> polyhedra have been identified [48]. For Zr‐Cu‐Al alloys processed via DCT, the SRO (0.2–0.4 nm) remains essentially unchanged, whereas the MRO (0.5–1.0 nm) is reduced [49]. Moreover, increasing pressure from 0 to 30 GPa leads to a higher fraction of <0,0,12,0>, <0,2,8,2>, and <0,1,10,2> clusters, accompanied by a decrease in <0,2,8,1> and <0,3,6,3> clusters [38]. MD simulation indicated that clusters such as <0,2,8,1> and <0,3,6,3> serve as soft indicator while clusters of <0,0,12,0>, <0,2,8,2>, and <0,1,10,2> act as dense/hard packed regions with decreased FV [39]. The transition between loosely‐packed clusters (soft regions) and densely‐packed clusters (hard regions) changes with processing parameters such as pressure/stress, and temperature, etc.

The Cu‐Zr and Zr‐Zr atomic pairs also play significant roles in structural relaxation and rejuvenation [51]. In rejuvenated Zr‐Cu‐Al glassy alloys, Zr, Cu, and Al atoms tend to concentrate in different types of atomic clusters. For instance, small Cu atoms predominantly occupy clusters such as <0,2,8,1>, <0,2,8,2>, <0,0,12,0>, and <0,3,6,3>, while large Zr atoms are more likely to dominate clusters such as <0,3,6,4>, <0,1,10,4>, and <0,2,8,4> [47, 54]. Similarly, Al‐centered atomic clusters are often found in the forms of <0,2,8,2>, <0,2,8,4>, <0,1,10,3>, <0,0,12,0>, and <0,1,9,3> [54]. Previous studies have frequently reported that the fraction of clusters such as <0,0,12,0> decreases in rejuvenated glassy alloys [47]. Moreover, the population of Zr‐, Cu‐, and Al‐centered icosahedral‐like Voronoi clusters (e.g., <0,0,12,0> and <0,1,10,x>) is also relatively reduced in the rejuvenated state [54]. For Zr_55_Cu_30_Al_10_Ni_5_ glassy alloy processed by combined CTC and lateral ESL, an increase in Al‐centered clusters accompanied by annihilation of Cu‐centered clusters has been observed [126]. Overall, the degree of rejuvenation is highly sensitive to the distribution of Al‐centered clusters, particularly the content of <0,0,12,0> [180].

Synchrotron radiation techniques have been employed to measure the packing density (or atomic volume) of as‐cast, relaxed, and rejuvenated glassy alloys [56]. Compared with as‐cast alloys, the packing density increases upon annealing but decreases after rejuvenation. A second heat treatment at a higher annealing temperature further reduces the packing density while promoting a more ordered atomic structure. The authors suggested that the relaxation enthalpy (Δ*H_rel_*) is closely related to both packing density and structural ordering [56]. In the as‐cast state, both packing density and ordering are relatively low, leading to the highest Δ*H_rel_*. Upon annealing, packing density and ordering increase, resulting in a reduction of Δ*H_rel_* (even approaching 0 kJ/mol). In contrast, rejuvenation decreases the packing density while increasing the degree of ordering, yielding an intermediate Δ*H_rel_*. Given the enhanced plasticity and fracture toughness observed in rejuvenated alloys, the authors concluded that packing density is likely the dominant factor governing the mechanical properties in this alloy system.

## 5 Derivative Processing Methods to Induce Rejuvenation

In addition to mechanical and thermal processing methods, other approaches such as ultrasonic and irradiation treatments have also been employed to induce rejuvenation in glassy alloys. These associated processing methods will be discussed separately in this chapter.

### 5.1 Ultrasonic Vibration Processing

#### 5.1.1 Types of Ultrasonic Vibration (UV) Processing Methods

Recently, the UV techniques have been increasingly applied in various fields related to processing amorphous alloys [83, 85, 87, 92, 181, 182, 183, 184, 185]. To date, more than ten types of UV techniques have been reported, including contact resonance ultrasonic actuation (CRUA), surface mechanical attrition treatment (SMAT), nanosecond laser shock peening (NLSP), ultrasonic plastic/metal welding (UPW), ultrasonic nano‐crystal surface modification (UNSM), ultrasonic pulse‐echo (UPE), ultrasonic vibration assisted shear punching (USP), ultrasonic vibration pre‐compression (UVPC) [85], ultrasonic additive consolidation (UAC), ultrasonic assisted soldering (UAS), ultrasonic beating forming (UBF), and ultrasonic elastic treatment (UET) [83]. These UV processing methods play distinct roles in influencing the degree of structural aging and rejuvenation in metallic glasses.

**FIGURE 22:**
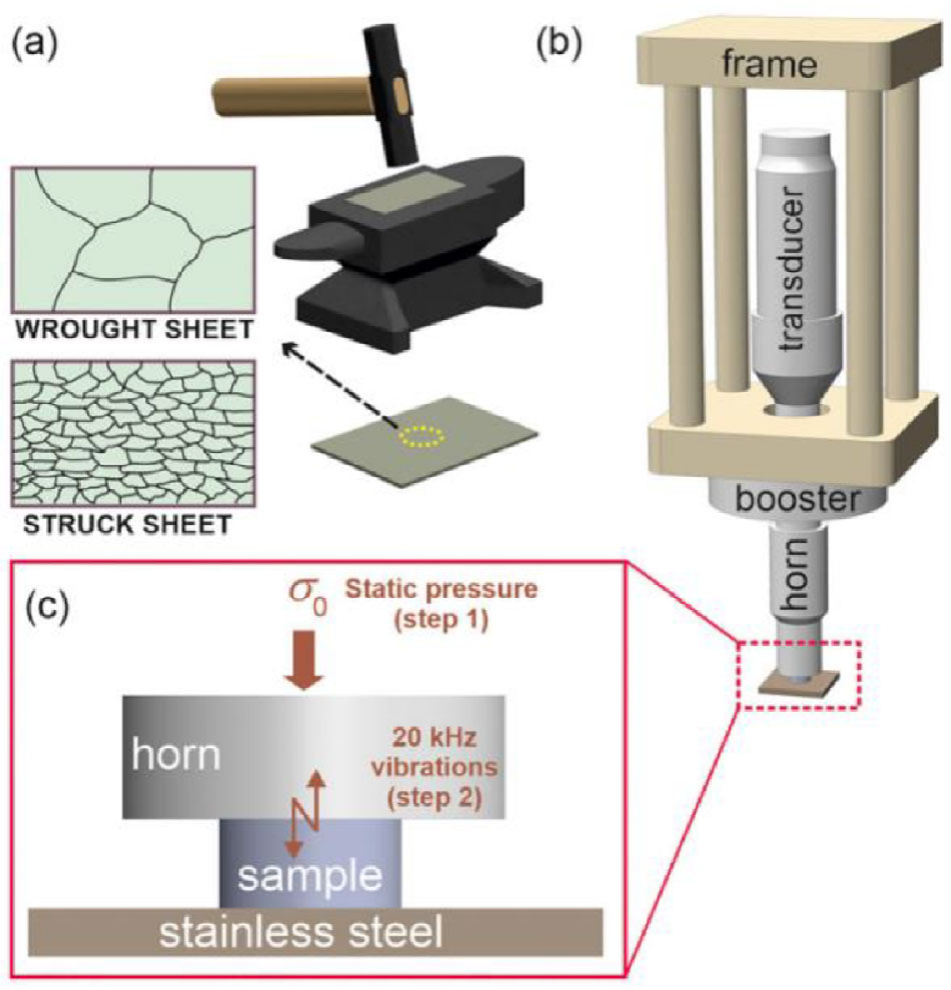
Hammer processing of alloys [83]: (a) when a successive impacts was applied on the processed the sheet of polycrystalline alloys, much work in terms of sound and heat can be observed and small impacts was stored to increase defect concentrations and refinement of the grains; (b) schematic setup of the ultrasonic hammer in this study for processing the glassy alloys; (c) schematic illustration of configuration for horn and sample under the loading conditions. Reprinted from ref. [83], copyright (2020), with permission from American Institute of Physics Publishing Group.

The application of UV techniques to glassy alloys offers several notable advantages. First, they can enhance the GFA and allow modification of physical dimensions of glassy alloys. Second, UV techniques can effectively activate energy state changes in amorphous alloys, such as structural rejuvenation. It should be noted, however, that the extent of such energy‐level changes is highly sensitive to both the initial energy state of alloys and the specific processing parameters. Therefore, it is crucial to identify as many types of UV techniques and their derivative counterparts as possible. The traditional UV rejuvenation process, consisting of multiple stages, is illustrated in Figure 22.

#### 5.1.2 Processing Parameters and Microstructures

##### 5.1.2.1 Processing Parameters

For the UV processing methods, loading stress, duration, and amplitude are three critical parameters. In contrast, for glassy alloys, alloy composition, initial energy state, and dimensions play equally important roles. Different combinations of UV processing parameters and intrinsic factors of glassy alloys can lead to diverse rejuvenated states in the alloys.

The amplitude of vibrations plays a critical role in determining whether a material undergoes rejuvenation or aging [181]. Specifically, small‐amplitude oscillations promote aging, whereas large‐amplitude oscillations induce rejuvenation. For example, at a shear strain amplitude of 2.5%, the potential energy decreases only slightly. When the strain amplitude increases to 5%, the potential energy decreases more significantly. At higher amplitudes of 7.5 and 10%, fluctuations and an eventual rise in potential energy are observed, signaling the onset of rejuvenation, also referred to as structural instability. Remarkable rejuvenation of glassy alloys occurs when the shear strain amplitude exceeds 10%. In summary, small strain amplitudes lead to aging, while larger amplitudes promote rejuvenation.

This raises the question: what is the relationship between the stability of processed alloys (aged or rejuvenated states) and the oscillatory vibrations of the system (shear strain amplitude)? The evolution of potential energy in glassy alloys provides an explanation. For rejuvenated alloys, oscillatory vibrations reduce the potential energy, which nonetheless remains higher than that of the as‐quenched state. In contrast, for aged alloys, the potential energy remains unchanged at a lower value, regardless of whether oscillatory vibrations are applied.

##### 5.1.2.2 Microstructures

The microstructures of rejuvenated glassy alloys induced by UV techniques generally exhibit several key features, such as relaxation enthalpy variations and the presence of loosely packed regions. Since UV methods can be classified into multiple categories, their corresponding microstructural evolutions should also be discussed separately.

For Ultrasonic Hammer (UH) processing, the relaxation enthalpy of a Zr_55_Al_10_Ni_5_Cu_30_ glassy rod initially increases under a stress of 3 MPa but decreases when the stress reaches 64 MPa [83]. In contrast, for the Zr_41.2_Ti_13.8_Cu_12.5_Ni_10_Be_22.5_ alloy (Vitreloy 1), the relaxation enthalpy first rises sharply and then fluctuates between the values of as‐cast and rejuvenated states. For instance, the most relaxed or slowest‐cooled glassy alloys (Δ*H_rel_* = 504 J/mol) can be significantly rejuvenated by UH treatment, whereas the less‐aged or fastest‐cooled alloys (Δ*H_rel_* = 591 J/mol) exhibit a smaller degree of rejuvenation. Alloys with intermediate cooling rates (Δ*H_rel_* = 547 J/mol) display a moderate rejuvenation effect. Similar structural transformations are also observed during UET [83]. These phenomena suggest that the stored oscillatory energy generated during UH processing can uniformly affect the entire alloy.

For the UVPC processing, rapid rejuvenation accompanied by a heterogeneous structure and improved plasticity has been achieved in Zr‐based BMGs [85]. This rapid rejuvenation is likely driven by the activation of loosely packed atomic regions, which occurs through synergistic effects of stress, thermal input, and ultrasonic field. Another contributing factor is the conversion of high‐frequency strain energy into thermal or internal energy. Both the stored enthalpy and the temperature rise are highly sensitive to the magnitude of UVPC [85, 186]. For example, the relaxation enthalpy of an as‐cast alloy (0.579 J/g) increased to 1.528 J/g after UVPC‐3 treatment. In terms of thermal response, a lower vibration amplitude (UVPC‐1) caused only a moderate temperature rise to 82°C, whereas a higher amplitude (UVPC‐3) produced a much larger increase, reaching 271°C.

For traditional UV loading, both as‐cast and aged glassy alloys with compositions of La_60_Ni_15_Al_25_ and La_55_Al_25_Ni_5_Cu_10_Co_5_ have been processed for various durations [92, 181, 183]. For alloys aged at 85% of the *T_g_* for up to 672 h, some main findings can be summarized as follows: (i) for long‐term aged amorphous alloys, the energy threshold required for rejuvenation increases correspondingly; (ii) the maximum plastic strain achieved is larger than that of as‐cast alloys; (iii) aged amorphous alloys can be recovered to their original states. For instance, at an energy release of 30 J, the sample aged for 168 h exhibits higher hardness than the as‐cast one. By contrast, when the release energy increases to 45 J, the same 168‐h aged alloy softens and collapses into a thin disk. These differences in deformation behaviors at different release energies may be attributed to changes in atomic spacing and formation of loosely packed amorphous structures.

Regarding origins of aging and rejuvenation, simulation studies on Cu_65_Zr_35_ and Ni_80_P_20_ glassy alloys suggest that rejuvenation arises from nonlinear responses and formation of nanoscale SBs, while aging is associated with local strain fields induced by string‐like cooperative atomic motion [181]. It should be noted, however, that this study did not attempt to investigate or compare the atomic configurations of the as‐cast, aged, and rejuvenated states, nor did it provide results from tensile tests. Softening behavior in rejuvenated glassy alloys has also been reported in Pd_40_Cu_30_P_20_Ni_10_ under UV loading [87]. Specifically, as the loading rate (mN/s) increases from 0.5 to 100, the hardness decreases sharply, while the elastic modulus first decreases and then increases. Atomic‐level analysis of Cu_65_Zr_35_ glassy alloys further revealed that the fraction of certain atomic clusters, such as <0,0,12,0>, initially increases and then decreases with increasing vibration amplitude [181].

One derivative of UV loading – the SMAT – was applied to a Cu_46_Zr_47_Al_7_ glassy alloy at a frequency of 20 kHz and an impact velocity of 10 m/s, resulting in formation of gradient amorphous structures across the sample thickness [182]. After SMAT processing, both surface roughness and relaxation enthalpy increased. Specifically, the surface roughness reached ∼360 nm, while the relaxation enthalpy rose from 3.54 J/g in the as‐cast state to 5.86 J/g after 25 min of treatment. A grain‐like microstructure was observed at a depth of ∼10 µm from the surface, consisting of dark regions (100–200 nm) and bright regions (10–50 nm). With increasing distance from the surface, the size of the dark regions expanded to 2–10 µm, whereas the bright regions remained nearly unchanged. At a depth of ∼120 µm, the grain‐like structure disappeared. Nevertheless, hardness continued to increase with depth, up to ∼150 µm from the surface.

### 5.2 Irradiation (Laser/ion) and Electric Current Rejuvenation

#### 5.2.1 Types of Rejuvenation Methods

**FIGURE 23:**
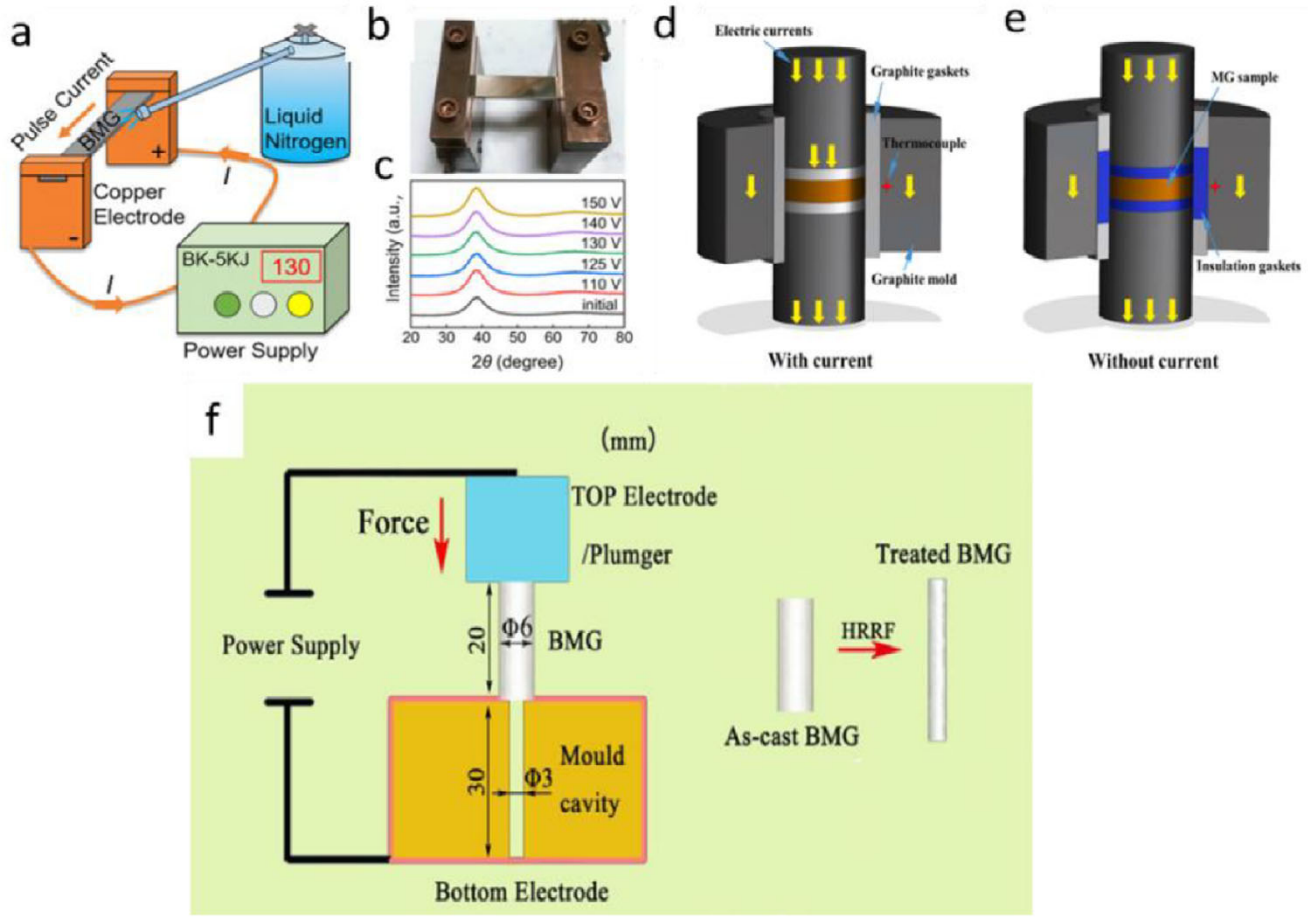
(a‐c) Schematic setup of the electro‐pulsing treatment (EPT) and XRD pattens of Zr‐Cu‐Al‐Ag glassy alloys before and after EPT at various voltages [88]; (d,e) schematics setup of designed equipment [191]: (d) with current and (e) without current; (f) schematic setup of high rheological rate of forming method (HRRF) [78]. Reprinted from refs. [78, 88, 191], copyright (2019, 2021, 2023), with permission from Elsevier BV, American Institute of Physics, and Elsevier Ltd Publishing Group.

Irradiation‐ or laser/ion‐based rejuvenation methods include electron beam (e‐beam) irradiation [82], swift heavy ion (SHI) irradiation [84], ion irradiation [81], laser engraving [187], laser shock peening [188, 189], and laser surface melting (LSM) [190]. Electric current–based rejuvenation methods, on the other hand, comprise electro‐pulsing treatment (EPT) [88], conventional electric current‐assisted sintering (ECAS) [191], high rheological rate forming (HRRF) [78], and pulsed electric current (PEC) [164, 192]. These rejuvenation approaches play different roles in altering microstructures, thereby leading to varying degrees of rejuvenation in glassy alloys. In the following section, these techniques will be discussed in detail (Figure 23).

##### 5.2.1.1 Irradiation/Laser/Ion Rejuvenation Methods

Electron beam (e‐beam) irradiation has been applied to process amorphous TiAl thin films in TEM [82]. The processing is strongly influenced by three key parameters: applied stress, e‐beam conditions, and irradiation time. Although prolonged irradiation (up to several hours) does not induce crystallization, it causes significant structural modifications. Both aging and rejuvenation phenomena have been observed. For glassy TiAl thin films, rejuvenation is characterized by an increase in mean atomic volume and average potential energy per atom. Compared with the non‐irradiated alloy, the irradiated structure (1.0% dose) exhibits a reduced number of 13‐atom icosahedral clusters, which is a distinct feature of rejuvenation.

The SHI irradiation has also been applied to modify microstructures of glassy alloys with compositions of Pd_40_Ni_40_P_20_ [84] and Pt_57.5_Cu_14.3_Ni_5.7_P_22.5_ [81]. Compared with their non‐irradiated counterparts, one of the key features observed after irradiation is mechanical softening, which is an important manifestation of rejuvenation. The SHI irradiation also induces notable structural changes, including an increase in MRO volume and a rise in *T_f_*. Processing temperature plays a crucial role in these effects. At low processing temperatures, irradiation‐induced modifications are more easily preserved and accumulated over time, eventually leading to significant structural transformations in the glassy alloys. In contrast, at higher processing temperatures, the retention and accumulation of these irradiation effects diminish, resulting in a reduced degree of structural change.

**FIGURE 24:**
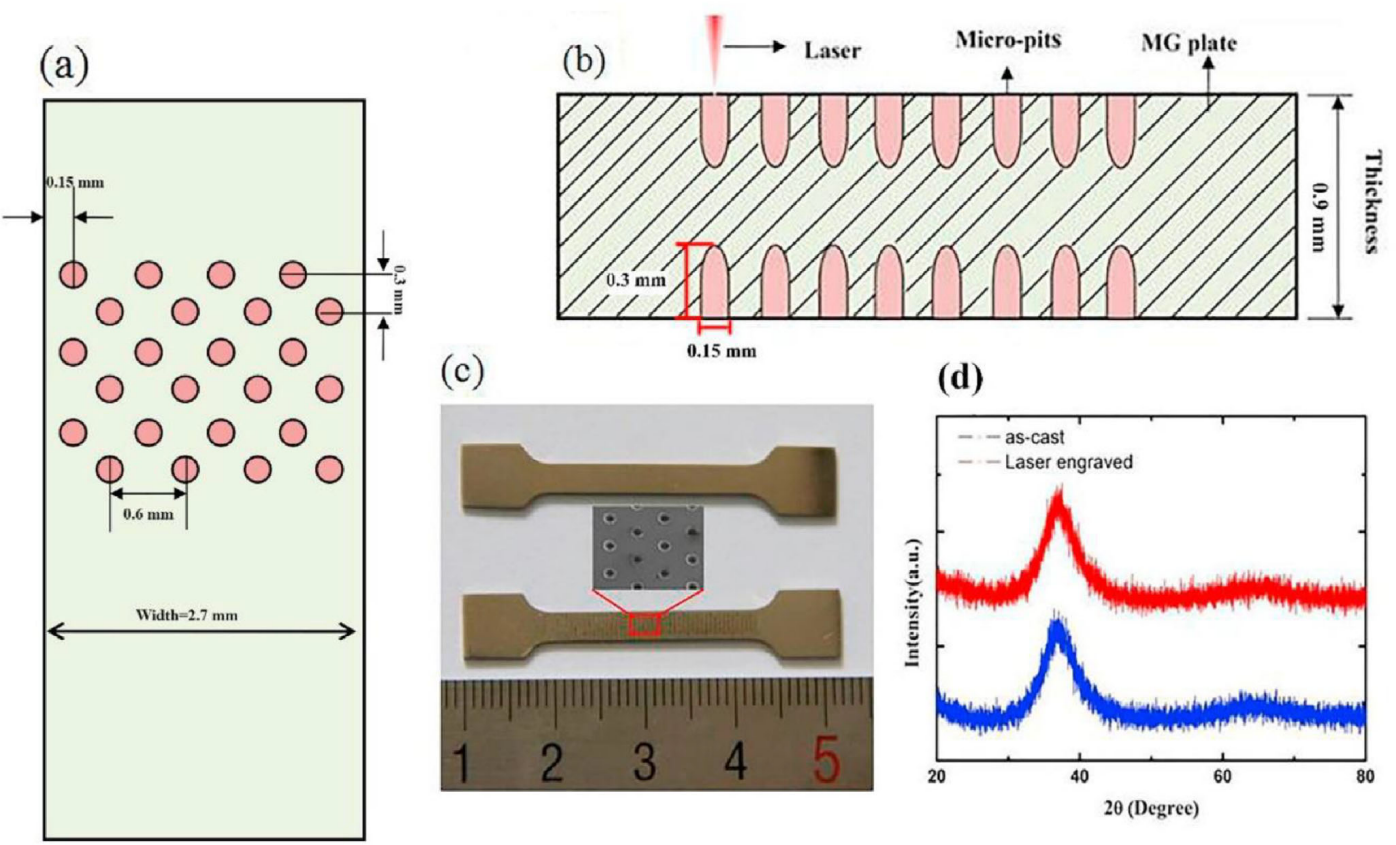
(a, b) The bullet shaped micro‐pits processed on both surfaces of glassy plates via the laser‐engraving technology; (c) the dog bone shaped as‐cast and Laser engraved samples; (d) XRD patterns of as‐cast and engraved samples [187]. Reprinted from ref. [187], copyright (2017), with permission from Elsevier BV Publishing Group.

A laser engraving technique was employed to fabricate a micro‐pit array, thereby activating a complex field [187]. The liquid‐like flow units induced by this complex field contribute to enhancing tensile plasticity. In the engraved samples, the formation of SBs can likely be suppressed, and numerous flame‐like cones are observed on the fracture surface. The laser‐engraved micro‐pits, the corresponding surface morphologies of samples as well as XRD patterns (amorphous states) are presented in Figure 24.

The LSP was applied to a glassy alloy with the composition Zr_35_Ti_30_Cu_10_Ni_5_Be_20_, and rejuvenated regions were observed near the surface [188]. The extent of these rejuvenated zones gradually decreases with increasing depth, while relaxed regions appear at a depth of approximately 1.5–2 mm from the surface. In addition, the processing angle between the laser scanning direction and the axial direction of plastic strain plays an important role. Specifically, the plastic strain induced at processing angles of 60–90° is about twice as large as that obtained at 0–30°, which may be attributed to residual stress. The impact velocity of the laser also exerts a positive influence on the rejuvenation behaviors of glassy alloys.

##### 5.2.1.2 Electric Current Rejuvenation Methods

**FIGURE 25:**
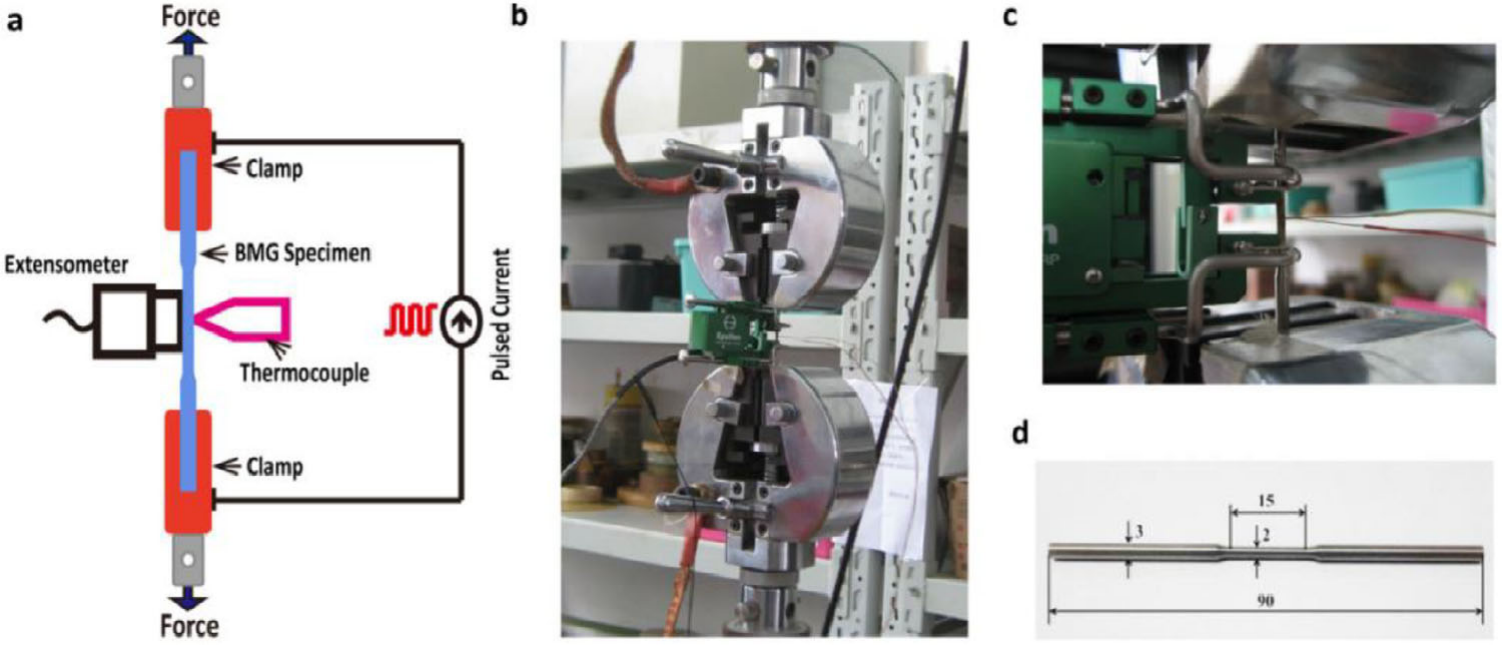
Schematic setup of tensile tests on Zr_55_Cu_30_Al_10_Ni_5_ BMG processed by the pulsed electric current [192]: (a) schematic setup of experimental design; (b) actual experimental operation of Zr_55_Cu_30_Al_10_Ni_5_ BMG; (c) the close‐up of the BMG sample, the thermocouple and the extensometer. Reprinted from ref. [192], copyright (2020), with permission from Springer Nature Publishing Group.

The PEC technique is another effective method for rejuvenating glassy alloys [192], with its experimental setup shown in Figure 25, and the direct current loading described in [193]. In a comparative study, two Zr_55_Cu_30_Al_10_Ni_5_ glassy alloys were examined: one subjected to both PEC treatment followed by external loading, and the other only to external loading. The alloy processed by PEC exhibited homogeneous elongation and pronounced necking, which can be attributed to an effective “temperature rise” and the Joule heating effect. During deformation, the transition from inhomogeneous to homogeneous flow in the glassy alloy appears to be closely related to an increase in current density and dynamic rejuvenation, i.e., the enhanced activation or volume fraction of STZs. In addition, the HRRF method has also been employed to process glassy alloys [78], with its setup shown in Figure 23f. The increase in stored energy introduced by HRRF may account for the enhanced compressive plasticity of 3.3%, which is notably higher than that of the as‐cast alloy.

#### 5.2.2 Processing and Microstructures

##### 5.2.2.1 Processing

For the HRRF process, the as‐cast glassy alloy with the composition Cu_45.75_Zr_46.75_Al_6.5_Co_1_ was first heated into the SCLRs using a rapid Joule heating device [78], after which mechanical forces were applied to squeeze the alloy while in the SCLRs. Following quenching, the treated glassy alloys became thinner and exhibited increased stored energy. During irradiation, increasing the accelerating voltage from 5 to 30 kV led to a higher volume fraction of irradiated regions [81]. The structural changes were also highly sensitive to irradiation intensity. Specifically, as the dosage increased from 0 to 3 dpa, the number of STZs increased correspondingly [194]. However, further increases in dosage diminished the rejuvenation effect.

##### 5.2.2.2 Microstructures

The PEC was applied at frequencies ranging from 1 to 20 kHz, with an optimized current density falling between 30 and 40 A/mm^2^ [192]. When processed under current densities below 30 A/mm^2^, the Cu_45.75_Zr_46.75_Al_6.5_Co_1_ alloy exhibited a fractured deformation behavior. At a current density of 33 A/mm^2^, a reduction in both yield stress and elastic modulus was observed, accompanied by homogeneous plastic deformation with a plastic strain of up to 16.6% and an ultimate tensile strength of 1050 MPa. Further increasing the current density to 35 and 38 A/mm^2^ resulted in pronounced softening, along with a plastic elongation of 16% in the treated glassy alloys. Although authors attributed the transition from inhomogeneous to homogeneous deformation primarily to the increase in current density rather than to temperature effects, the influence of temperature rise should not be overlooked. Specifically, the alloy temperatures at 523, 553, 603, and 643 K correspond to current densities of 30, 33, 35, and 38 A/mm^2^, respectively. For HRRF‐treated alloys, the relaxation enthalpy was found to increase [78]. For instance, the enthalpy of the as‐cast alloy was 2.171 J/g, while the quenched alloy with a diameter of 3 mm exhibited an enthalpy of 4.285 J/g, and the treated as‐cast alloy with a diameter of 6 mm showed an enthalpy of 3.901 J/g. These results indicate that larger‐diameter glassy alloys exhibit a smaller increase in relaxation enthalpy after HRRF treatment compared with their smaller‐diameter counterparts.

Upon electric current processing, enhanced structural fluctuations at atomic scale were observed [191]. Specifically, (i) a reduction in the coordination of Zr‐ and Cu‐centered clusters accompanied by an increase in Al‐centered icosahedra within amorphous matrix; (ii) an increase in volume fraction of 1‐atom and 2‐atom clusters, together with a decrease in 3‐atom and 4‐atom clusters; (iii) a higher fraction of loosely packed amorphous structures in terms of MRO; and (iv) a remarkable increase in relaxation enthalpy, from 0.14 J/g in the as‐quenched state to 2.03 J/g in the treated glassy counterpart. More specifically, in the treated alloys, the most dominant Voronoi clusters centered at Zr, Cu, and Al atoms are Zr<0,2,8,5>, Cu<0,0,12,0>, and Al<0,0,12,0>, with respective volume fractions of 11.5, 14.7, and 16.9%. Voltage also plays a critical role in governing microstructures and degree of rejuvenation [88]. For instance, voltages below 110 V exert a negligible influence, whereas voltages around 130 V have a pronounced effect, leading to reduced density, modulus, and hardness, as well as enhanced *β* relaxation. The relaxation enthalpy exhibits a peak at 125–130 V, beyond which it decreases with further voltage increase.

Upon deformation of peened alloys, two competing mechanisms are activated: softening induced by SBs and hardening arising from residual stresses [195]. Shot‐peening can also trigger both structural relaxation and rejuvenation processes [196]. Glassy alloys were processed by shot‐peening at two temperatures, 77 and 298 K. The relaxation enthalpies of the two treated alloys differ, with significantly higher enthalpy observed at the lower temperature. Specifically, at 77 K, the enthalpy increases with processing time, reaching a maximum at 60 s, and then decreases with further extension of processing. The influence of processing temperature (77 vs. 298 K) on alloys with different relaxation states further shows that the stored energy (or relaxation enthalpy) at 77 K is about 30 times higher than that at 298 K for highly relaxed alloys. In contrast, for less relaxed alloys, the difference is reduced to about fourfold. This indicates that highly relaxed alloys can be much more effectively rejuvenated at low temperature.

Another derivative technique, the UV loading – nonlinear shot peening (NLSP) – has also been employed to rejuvenate Ti‐based and Zr‐based glassy alloys [186]. Work‐softening was observed in the shock‐affected zones. With increasing depth from the treated surface, hardness decreases, whereas relaxation enthalpy increases, reaching 0.995 and 3.817 J/g for Ti‐based and Zr‐based glassy alloys, respectively. The SLM has likewise been applied to process glassy alloys [197]. In this case, the relaxation enthalpy increases from 4–5 J/g in quenched alloys to 9–11 J/g in SLM‐processed alloys, accompanied by a decrease in average hardness. For irradiated alloys, ductile deformation has also been observed at high ion fluences [81]. For other rejuvenation methods, some macroscopic features such as hardness and elastic modulus as a function of various processing parameters can be found in Table 4.

**TABLE 4:** Hardness and elastic modulus as well as relaxation enthalpy as a function of processing parameters.

Alloy composition	Processing parameters	Hardness (GPa)	Elastic modulus (GPa)	Relaxation enthalpy (J/mol)	Relaxation enthalpy (J/g)	References
Zr_55_Cu_30_Al_10_Ni_5_	Smooth one	6.48± 0.06	95.9± 0.81			[77]
Zr_55_Cu_30_Al_10_Ni_5_	Defected one	6.09± 0.03	92.3± 1.08			[77]
Zr_55_Cu_30_Al_10_Ni_5_	As‐cast				∼1.14	[191]
Zr_55_Cu_30_Al_10_Ni_5_	With current				∼2.03	[191]
Cu_45.75_Zr_46.75_Al_6.5_Co_1_	As‐cast				2.17	[78]
Cu_45.75_Zr_46.75_Al_6.5_Co_1_	3‐mm treated HRRF				4.285	[78]
Cu_45.75_Zr_46.75_Al_6.5_Co_1_	6‐mm treated HRRF				3.901	[78]
Zr_44_Ti_11_Ni_10_Cu_10_Be_25_	Cool and load at 215/s			210		[198]
Zr_44_Ti_11_Ni_10_Cu_10_Be_25_	Cool and load at 830/s			810		[198]
Cu_40.6_Ti_37.5_Zr_9.4_Ni_9.4_Sn_3.1_	Mechanical mill for 0 h			558		[142]
Cu_40.6_Ti_37.5_Zr_9.4_Ni_9.4_Sn_3.1_	Mechanical mill for 10 h			1143		[142]
Cu_40.6_Ti_37.5_Zr_9.4_Ni_9.4_Sn_3.1_	Mechanical mill for 15 h			1529		[142]
Cu_40.6_Ti_37.5_Zr_9.4_Ni_9.4_Sn_3.1_	Mechanical mill for 20 h			1693		[142]
Cu_40.6_Ti_37.5_Zr_9.4_Ni_9.4_Sn_3.1_	Mechanical mill for 25 h			1896		[142]
Cu_40.6_Ti_37.5_Zr_9.4_Ni_9.4_Sn_3.1_	Mechanical mill for 30 h			2122		[142]
Cu_40.6_Ti_37.5_Zr_9.4_Ni_9.4_Sn_3.1_	Mechanical mill for 35 h			2245		[142]
Cu_40.6_Ti_37.5_Zr_9.4_Ni_9.4_Sn_3.1_	Mechanical mill for 40 h			2424		[142]
Cu_40.6_Ti_37.5_Zr_9.4_Ni_9.4_Sn_3.1_	Mechanical mill for 45 h			2389		[142]
Cu_40.6_Ti_37.5_Zr_9.4_Ni_9.4_Sn_3.1_	Mechanical mill for 55 h			2387		[142]
Cu_40.6_Ti_37.5_Zr_9.4_Ni_9.4_Sn_3.1_	Mechanical mill for 65 h			2393		[142]
Cu_40.6_Ti_37.5_Zr_9.4_Ni_9.4_Sn_3.1_	Mechanical mill for 80 h			2431		[142]
Cu_40.6_Ti_37.5_Zr_9.4_Ni_9.4_Sn_3.1_	Mechanical mill for 100 h			2454		[142]
Zr_52.5_Cu_17.9_Ni_14.6_Al_10_Ti_5_	As‐cast	6.8			0.579	[85]
Zr_52.5_Cu_17.9_Ni_14.6_Al_10_Ti_5_	UVPC‐1				0.826	[85]
Zr_52.5_Cu_17.9_Ni_14.6_Al_10_Ti_5_	UVPC‐2				0.869	[85]
Zr_52.5_Cu_17.9_Ni_14.6_Al_10_Ti_5_	UVPC‐3	6.6			1.528	[85]
Zr_52.5_Cu_17.9_Ni_14.6_Al_10_Ti_5_	As‐cast	6.83± 0.11			4‐5	[197]
Zr_52.5_Cu_17.9_Ni_14.6_Al_10_Ti_5_	SLM	6.68± 0.14			9‐11	[197]

### 5.3 Defect‐Imprinting to Induce Rejuvenation

#### 5.3.1 Types of Surface Imprinting

Surface modification methods generally include the imprinting method [77], the surface screw thread shape (STS) method, and the fabrication of micro‐pit arrays on alloy surfaces using laser engraving techniques [187]. Certain surface defects, such as geometrical and mechanical heterogeneities in Zr_65_Fe_5_Al_10_Cu_20_ BMG, can be introduced through rapid defect‐printing (RDP) treatment [77]. These geometrical and mechanical heterogeneities help modulate the local stress state and gradient, thereby generating heterogeneous and localized stress concentrations. Plastic deformation processes, such as imprinting, can further lead to formation of spatially heterogeneous structures, including structural anisotropy in both magnitude and direction [199].

**FIGURE 26:**
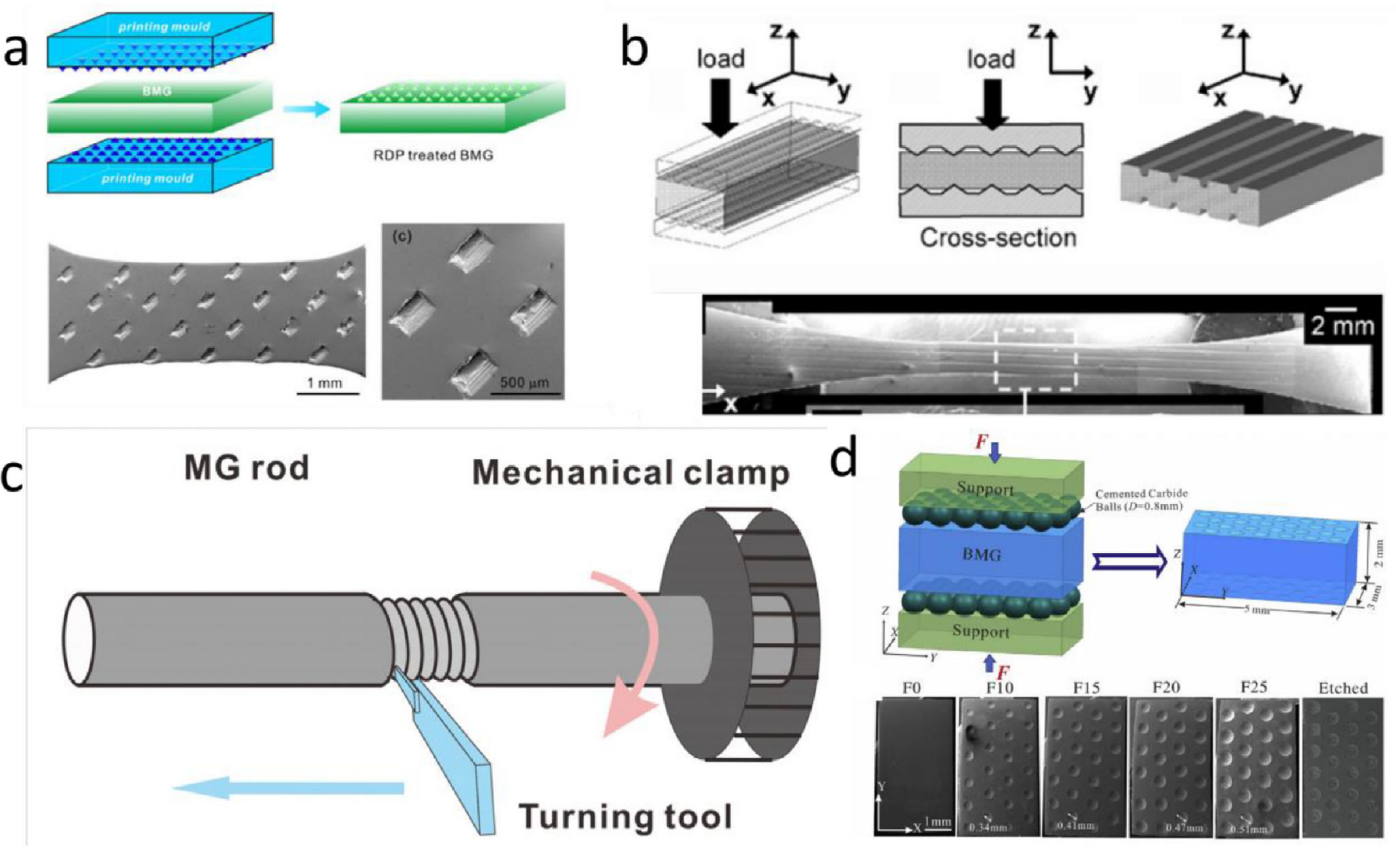
(a) Schematics setup of processes of rapid defect‐printing (RDP) treatment and obtained samples or tension [77]; (b) schematic map of the imprinting process together with resulting surface morphology [80]; (c) the illustration of STS structures developed by the mechanical turning on the surface of glassy rod [86]; (d) schematic illustration of the surface imprinting method in the study and corresponding SEM images [200]. Reprinted from refs. [77, 80, 86, 200], copyright (2011, 2013, 2016, 2021), with permission from Elsevier Ltd, and Elsevier BV Publishing Group.

The imprinting technique has been applied to Cu foil and Zr‐based glassy alloys to fabricate specific geometrical patterns [201]. Such patterned structures enable the otherwise brittle glassy alloys to exhibit homogeneous plastic flow without shear fracture at room temperature. It has been demonstrated that the compressive residual stress generated during imprinting, rather than the geometrical pits themselves, plays a dominant role in enhancing plasticity and fracture strength [200]. The resulting gradient stress effectively suppresses the dynamics of shear band propagation. Furthermore, a STS structure has also been developed in glassy alloys [86]. This unique STS configuration alters the stress field distribution and thereby modifies the initiation and evolution of SBs. The fabricated STS structure is shown in Figure 26. According to authors, the twisted stress field induced by the STS method is analogous to that generated under torsional loading, which produces a restricted stress field. In this sense, the twisted stress field in the STS structure can be regarded as a form of restricted stress.

#### 5.3.2 Processing and Microstructures

##### 5.3.2.1 Processing

For the RDP treatment reported in ref. [77], the key parameters include the designed shape and distribution of the defect imprinting. In the imprinting method described in ref. [80], the critical parameters are the shape of the periodic array of trapezoidal linear teeth and the applied load. By contrast, for the STS structures fabricated by mechanical turning [86], the depth of the STS structure serves as a crucial parameter. In the case of surface imprinting in Ref. [200], the essential parameters are the close‐packed arrangements, such as AB‐stacking or AA‐stacking patterns, together with the applied imprinting forces.

##### 5.3.2.2 Microstructures

Structural analysis reveals that imprinted sites are heterogeneous network domains composed of alternating hard and soft regions. These surface imprints or defects act as geometrical and mechanical heterogeneities, which promote the activation of multiple SBs and facilitate the homogenization of plastic deformation [77]. The fractured surface, characterized by numerous flame‐like cones of ∼10 µm in size, is strongly influenced by micro‐pits [187]. A high density of SBs was observed in fractured alloys containing imprinted defects.

To elucidate effects of imprinting on both plasticity and strength, defect‐free (smooth) samples were compared with samples containing defects. Although the average ultimate tensile stress of defected samples decreases, their tensile elongation correspondingly increases, surpassing that of smooth samples. Moreover, both the average hardness and elastic modulus decrease slightly, from 6.5 and 95.5 GPa in smooth alloys to 6.1 and 92.3 GPa, respectively.

The imprinted defects may play three roles in modifying deformation: (i) serving as nucleation sites for multiple SBs, (ii) inducing local stress concentration, and (iii) creating a negative stress gradient. Simulation results indicate that, around imprinting defects, the interfaces between the defects and the amorphous matrix provide favorable sites for SB branching and rotation [202]. Both the loading angle and the defect–matrix interfaces make significant contributions to the mechanical properties of glassy alloys [203]. In particular, increasing the loading angle (between the imprints and the tensile direction) from 0° to 45° enhances both strength and ductility. A larger loading angle promotes the branching and rotation of SBs toward the orientation of the imprinting defects. Meanwhile, the interfaces between the defects and the glassy matrix further guide the deviation of SB paths.

Another study indicates that with an increase in LSTT pore size, tensile strain increases, whereas the elastic modulus and fracture strength decrease [203]. The arrangement of surface pores, such as the stacking of LSTT pores, can be regarded as the distribution of a secondary soft phase. Three types of LSTT pores, shown in Figure 26, demonstrate the close relationship between pore morphology and fracture surface. For LSTT sample A, a radial‐like fracture pattern with relatively small features corresponds to the presence of smaller pores. In contrast, LSTT sample B exhibits vein‐like and river‐like fracture patterns dominated by shear stress. For LSTT sample C, dense micro‐sized core‐like structures are observed, with sizes of approximately 7.5 µm. Moreover, an increase in the number of surface pores – corresponding to a higher volume fraction of ductile phases – results in reduced strength and enhanced plastic strain. The morphology of the fracture surface and the formation of SBs are also strongly influenced by the angle, size, and distribution of the surface pores.

Atomic arrangement, particularly structural anisotropy, can be tailored by STS [199]. The STS structure plays two critical roles: (i) twisting the distribution of the stress field, and (ii) suppressing the propagation of primary SBs while promoting the formation of multiple secondary SBs [86]. Two types of STS structures have been designed and fabricated. Compared with larger‐sized STS, smaller‐sized STS leads to higher fracture strength; however, the processed alloys still exhibit brittleness. In contrast, the larger‐sized STS induces a transition from brittle to plastic behavior, accompanied by serrated flow in glassy alloys as the tensile strain increases to 1.37%, and fracture morphologies characterized by a mixture of river‐like and radial‐like patterns. For the smaller‐sized STS structure, deformation is dominated by discontinuous and intermittent propagation of primary SBs.

### 5.4 Increasing Cooling Rate (Static Quenching)

Cooling rate directly determines the GFA, thereby influencing the atomic structures of BMGs. For instance, Ti_45_Cu_40_Ni_7.5_Zr_5_Sn_2.5_ BMGs with diameters of 2 and 3 mm have been successfully fabricated [204]. Compared with the 3 mm sample, the 2 mm Ti‐based BMG exhibits enhanced plasticity (fracture strain of 16.7%) and higher strength (ultimate strength of 2277 MPa). Microstructural analyses reveal compositional heterogeneity across different regions, which may account for these significant variations in mechanical properties. These chemical inhomogeneities are the macroscopic manifestations of underlying atomic structures. Previous studies [6, 7, 9] have suggested that such differences in mechanical performance are closely related to the atomic‐level structural features of amorphous alloys. The atomic structures, consisting of various SROs and MROs with differences in length scale, elemental distribution, angular configuration, and spatial arrangement, play a decisive role in governing plastic strain and work‐hardening behavior.

One representative example is the Fe_50_Ni_30_P_13_C_7_ BMG, which exhibits exceptional compressive plasticity exceeding 15%. Its main deformation mechanism has been attributed to the coexistence of hard zones and soft regions composed of homogeneously dispersed nanocrystals [7, 8]. If these nanocrystals are replaced by SROs structures and/or loosely packed MROs structures, the enhanced plasticity and pronounced work‐hardening behaviors can still be maintained [9]. In this regard, designing heterogeneous structures with alternating soft and hard regions (loosely packed MROs and SROs) provides an effective strategy to endow BMGs with improved plasticity and work‐hardening ability.

For instance, static quenching has been employed to produce Zr_60_Ni_25_Al_15_ BMGs, achieving an improved plastic strain of 6.5% [205], in stark contrast to the ∼0.5% plasticity observed in alloys fabricated via conventional suction casting. This remarkable difference is believed to arise from the formation of distinct atomic clusters under different cooling rates. Similarly, other processing techniques, such as flux treatment, have been shown to effectively tailor atomic‐scale structures [144]. In flux‐treated Fe‐Ni based BMGs, the coexistence of ILCs and CLCs has been observed, resulting in a dramatic enhancement of compressive plasticity to above 50%. Furthermore, the flux treatment has been associated with a decrease in both coordination number and fraction of MROs. Composition tuning also plays a significant role. For example, in the Zr‐(Cu,Ag)‐Al system, increasing the Zr content tends to reduce the fraction of icosahedral clusters, which in turn benefits the plastic strain [145].

**FIGURE 27:**
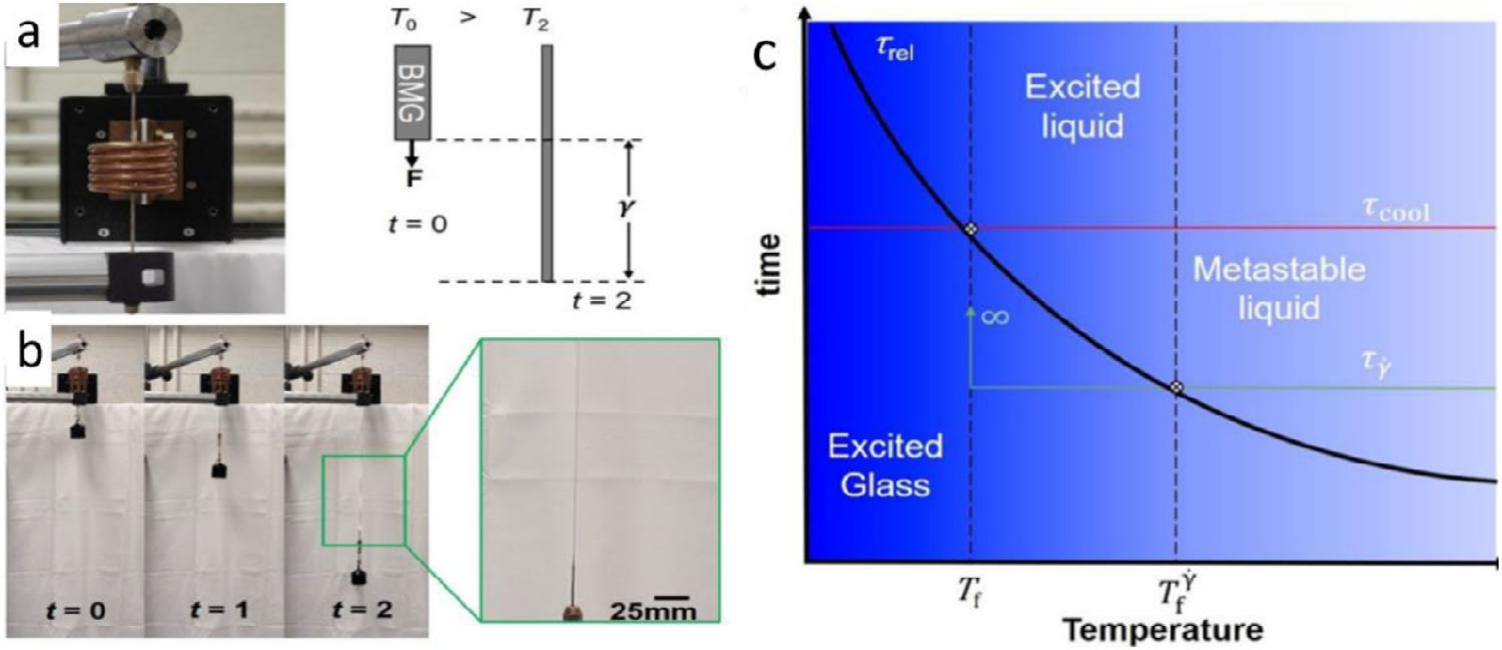
Schematic setup of experiment aiming to excite liquid cooling [141]: (a) induction device to heat glassy rods to a desired temperature of SCLRs; (b) a constant force ranging from 1 to 100 N aims to strain the glassy rods. Upon straining, various cooling rates have been applied to the processed alloys in air. The different cooling rates can be calculated on the basis of measuring decreased and initial diameter of glassy alloys; (c) snap shots of the deforming glassy rods in its SCLR; (d) schematic setup of the characteristic time scales involved in the excited liquid cooling mechanisms as a function of temperature. Reprinted from ref. [141], copyright (2021), with permission from Springer Nature Publishing Group.

An applied strain rate can be used to excite the liquid, which is then retained in glassy alloys through rapid quenching at a significantly high cooling rate [198], as illustrated in Figure 27. Authors argued that exciting the liquid followed by rapid cooling represents one of the most effective approaches to increase the potential energy of glassy alloys. According to their findings, applying a sufficiently high strain rate within a narrow temperature interval induces an accelerated cooling process. This, in turn, leads to an elevated *T_f_* and structural dilation caused by the imposed strain. The increase in *T_f_* is strongly dependent on the mismatch between the time scales imposed by the external load and those inherent to cooling. Typically, a higher cooling rate results in a higher *T_f_*, with reported increases of up to 60 K. Notably, enhanced plastic ductility has been closely correlated with elevated *T_f_*. For instance, in Zr_44_Ti_11_Cu_10_Ni_10_Be_25_ glassy alloy processed by this method, the bending ductility was tripled. Consistently, the relaxation enthalpy also increases with strain rate [198]. Specifically, a maximum relaxation enthalpy of 0.81 kJ/mol was observed at a strain rate of 830 s^−1^, compared to 0.21 kJ/mol at 215 s^−1^.

## 6 Mechanical Properties

### 6.1 Plasticity and Strength

The plasticity and strength of glassy alloys can be compared to a teeterboard: when one side reaches its highest point, the other remains at its lowest. In this analogy, the relationship between plasticity and strength follows the motion pathway of the teeterboard. The plasticity, strength, and hardness of the alloys, together with their compositions, are summarized in Table 5. As shown in Table 5, rejuvenated glassy alloys exhibit a wide range of mechanical properties. These variations are likely attributable to the diverse microstructures that develop during processing. Based on these processing routes, the observed plasticity values can be categorized into several groups: mechanical processing, thermal rejuvenation, and other types of treatments. Previous studies have reported that the degree of rejuvenation plays a crucial role in determining mechanical properties, particularly plasticity [73]. Partially rejuvenated glassy alloys generally show higher plasticity compared to their deeply rejuvenated counterparts, which may result from differences in the amount of nano‐clusters formed and the occurrence of stress‐induced crystallization (SIC) during deformation.

**TABLE 5:** Plasticity, strength of rejuvenated glassy alloys via various rejuvenation methods.

Alloy composition	Processing parameters	(Yielding) Strength (MPa)	(Fracture) strength (MPa)	Plasticity (%)	References
Zr_52.5_Cu_17.9_Ni_14.6_Al_10_Ti_5_	HPT: N = 10	1776± 30		0.30± 0.02	[61]
	HPT: N = 20	1484± 29		0.32± 0.02	[61]
	HPT: N = 40	1460± 32		0.47± 0.02	[61]
Cu_50_Zr_50_	Preload to 90%σ_*y*_	1600 ± 100		4.40± 0.4	[124]
	As‐cast one	1600 ± 100		4.00± 1.0	[124]
Cu_57_Zr_43_	Preload to 90%σ_*y*_	1600 ± 100		4.10± 0.3	[124]
	As‐cast one	2000± 100		0.70± 0.2	[124]
Cu_65_Zr_35_	Preload to 90%σ_*y*_	1700± 100		4.30± 0.5	[124]
	As‐cast one	2200± 100		0.00± 0.0	[124]
Zr_64.13_Cu_15.75_Ni_10.12_Al_10_	As‐cast one			∼80%	[137]
Zr_64.13_Cu_15.75_Ni_10.12_Al_10_	Aged 6 ys	1750		∼27.3%	[137]
Zr_64.13_Cu_15.75_Ni_10.12_Al_10_	Aged 6ys +load 60%σ_*y*_	2650		∼39.4%	[137]
Zr_64.13_Cu_15.75_Ni_10.12_Al_10_	Aged 6.5 ys	1750		∼22%	[137]
Zr_64.13_Cu_15.75_Ni_10.12_Al_10_	Aged 6.5 ys +load 60%σ_*y*_	2050		∼32%	[137]
Zr_64.13_Cu_15.75_Ni_10.12_Al_10_	Aged 8ys	1500		0	[137]
Zr_64.13_Cu_15.75_Ni_10.12_Al_10_	Aged 8ys+load 60%σ_*y*_	1750		∼5%	[137]
Zr_55_Cu_30_Al_10_Ni_5_	As‐cast one	1706± 13	1835± 20	3.75± 0.10	[130]
Zr_55_Cu_30_Al_10_Ni_5_	ESL 40% of σ_*y*_	1679± 15	1855± 25	4.12± 0.12	[130]
Zr_55_Cu_30_Al_10_Ni_5_	ESL 70% of σ_*y*_	1665± 16	1848± 30	4.47± 0.10	[130]
Zr_55_Cu_30_Al_10_Ni_5_	ESL 80% of σ_*y*_	1649± 11	1875± 18	5.29± 0.20	[130]
Zr_55_Cu_30_Al_10_Ni_5_	ESL 90% of σ_*y*_	1620± 14	1916± 22	8.97± 0.18	[130]
Zr_64.13_Cu_15.75_Ni_10.12_Al_10_	As‐cast			2.5	[28]
Zr_64.13_Cu_15.75_Ni_10.12_Al_10_	PA‐ESL + 1h		1904	3.5	[28]
Zr_44_Ti_11_Cu_9.8_Ni_10_Be_25_	As‐cast		1890	<0.5%	[122]
Zr_44_Ti_11_Cu_9.8_Ni_10_Be_25_	Cold roll		2600	15%	[122]
Zr_55_Ti_5_Al_10_Cu_20_Ni_10_	As‐cast			<0.5%	[122]
Zr_55_Ti_5_Al_10_Cu_20_Ni_10_	Cold roll			15%	[122]
Zr_61_Cu_25_Al_12_Ti_2_	As‐cast	1440± 160			[39]
Zr_61_Cu_25_Al_12_Ti_2_	Heating to 673 K	1460± 40			[39]
Zr_61_Cu_25_Al_12_Ti_2_	Heating to 773 K	1420± 60		1.0%	[39]
Zr_61_Cu_25_Al_12_Ti_2_	Heating to 813 K	1240± 110		1.4%	[39]
Zr_59.5_Nb_4.8_Cu_14.4_Ni_11.6_Al_9.7_	As‐cast	1733		0.9%	[67]
Zr_59.5_Nb_4.8_Cu_14.4_Ni_11.6_Al_9.7_	10 number of DCT	1744		1.25%	[67]
Zr_59.5_Nb_4.8_Cu_14.4_Ni_11.6_Al_9.7_	20 number of DCT	1755		2.25%	[67]
Zr_59.5_Nb_4.8_Cu_14.4_Ni_11.6_Al_9.7_	30 number of DCT	1745		1.3%	[67]
Zr_59.5_Nb_4.8_Cu_14.4_Ni_11.6_Al_9.7_	40 number of DCT	1740		0.85%	[67]
Ba_96_Nb_4_	As‐cast	>4200		2.3%	[51]
Ba_96_Nb_4_	CTC 353 K	>4200		3.8%	[51]
Ba_96_Nb_4_	CTC 373 K	>4200		4.7%	[51]
Ba_96_Nb_4_	CTC 393 K	4350		7.4%	[51]
Ba_96_Nb_4_	CTC 433 K	>4200		3.6%	[51]
Ba_96_Nb_4_	CTC 473 K	>4200		1.7%	[51]
Ba_96_Nb_4_	CTC 513 K	4050		6.1%	[53]
Ba_96_Nb_4_	CTC 533 K	4180		3.9%	[53]
Ba_96_Nb_4_	CTC 563 K	4230		1.8%	[53]
Zr_58_Cu_22_Fe_8_Al_12_	As‐cast	2150		0.91%	[8]
Zr_58_Cu_22_Fe_8_Al_12_	T6	2140		1.35%	[8]
Zr_58_Cu_22_Fe_8_Al_12_	T25	2195		2.3%	[8]
Zr_58_Cu_22_Fe_8_Al_12_	T70	2215		4.65%	[8]
Zr_58_Cu_22_Fe_8_Al_12_	T150	2240		4.95%	[8]
Zr_58_Cu_22_Fe_8_Al_12_	T_fc_ = 675 K	2150		3.25%	[8]
Zr_58_Cu_22_Fe_8_Al_12_	T_fc_ = 753 K	2135		5.7%	[8]
Zr_58_Cu_22_Fe_8_Al_12_	T_fc_ = 711 K	2140		6.25%	[8]
Zr_46_Cu_46_Al_8_	As‐cast	1838± 40		0.22± 0.06	[179]
Zr_46_Cu_46_Al_8_	10 numbers of cycling	1845± 30		0.50± 0.08	[179]
Zr_46_Cu_46_Al_8_	20 numbers of cycling	1862± 36		0.82± 0.06	[179]
Zr_46_Cu_46_Al_8_	60 numbers of cycling	1760± 55		2.02± 0.12	[179]
Ti_20_Zr_20_Hf_20_Be_20_Cu_20_	As‐cast	1990± 20	2027± 30	0.62	[150]
Ti_20_Zr_20_Hf_20_Be_20_Cu_20_	DCT 5	1818± 28	2007± 51	2.10	[150]
Ti_20_Zr_20_Hf_20_Be_20_Cu_20_	DCT 10	1807± 35	2076± 58	3.11	[150]
Ti_20_Zr_20_Hf_20_Be_20_Cu_20_	DCT 15	1820± 31	2081± 33	3.64	[150]
Ti_20_Zr_20_Hf_20_Be_20_Cu_20_	DCT 20	1831± 42	2114± 47	5.38	[150]
Zr_55_Cu_30_Al_10_Ni_5_	As‐cast	1876	2070	6.6%	[91]
Zr_55_Cu_30_Al_10_Ni_5_	DCT 30	1706	2165	12.7%	[91]
(Zr_0.55_Cu_0.3_Ni_0.05_Al_0.1_)_99_Ta_1_	As‐cast	1660	2080	5.8%	[175]
(Zr_0.55_Cu_0.3_Ni_0.05_Al_0.1_)_99_Ta_1_	DCT30	1440	2132	13.9%	[175]
(Zr_0.55_Cu_0.3_Ni_0.05_Al_0.1_)_97_Ta_3_	As‐cast	1638	2072	7.0%	[175]
(Zr_0.55_Cu_0.3_Ni_0.05_Al_0.1_)_97_Ta_3_	DCT30	1520	2133	14.7%	[175]
(Zr_0.55_Cu_0.3_Ni_0.05_Al_0.1_)_95_Ta_5_	As‐cast	1620	2122	10.5%	[175]
(Zr_0.55_Cu_0.3_Ni_0.05_Al_0.1_)_95_Ta_5_	DCT30	1568	2161	16.2%	[175]
Zr_46_Cu_46_Al_8_	As‐cast	1775± 50	1955± 50	3.7± 0.6	[54]
Zr_46_Cu_46_Al_8_	Relax	1795± 40	2052± 40	2.4± 0.2	[54]
Zr_46_Cu_46_Al_8_	Rej300	1727± 30	2059± 30	3.6± 0.5	[54]
Zr_46_Cu_46_Al_8_	DCT30	1646± 50	2039± 50	4.8± 0.6	[54]
Zr_52.5_Cu_17.9_Ni_14.6_Al_10_Ti_5_	As‐cast			5.15± 0.74	[56]
Zr_52.5_Cu_17.9_Ni_14.6_Al_10_Ti_5_	Relaxed at 570 K for 9h			3.42±.08	[56]
Zr_52.5_Cu_17.9_Ni_14.6_Al_10_Ti_5_	Heating to 640 K			3.23± 1.03	[56]
Zr_52.5_Cu_17.9_Ni_14.6_Al_10_Ti_5_	Heating to 650 K			7± 0.665	[56]
Zr_52.5_Cu_17.9_Ni_14.6_Al_10_Ti_5_	Heating to 655 K			7.25± 1.5	[56]
Zr_52.5_Cu_17.9_Ni_14.6_Al_10_Ti_5_	Heating to 660 K			8.53± 1.74	[56]
Zr_52.5_Cu_17.9_Ni_14.6_Al_10_Ti_5_	Heating to 661 K				[56]
Zr_52.5_Cu_17.9_Ni_14.6_Al_10_Ti_5_	Heating to 662 K				[56]
Zr_52.5_Cu_17.9_Ni_14.6_Al_10_Ti_5_	Heating to 663 K				[56]
Zr_52.5_Cu_17.9_Ni_14.6_Al_10_Ti_5_	Heating to 664 K				[56]
Zr_52.5_Cu_17.9_Ni_14.6_Al_10_Ti_5_	Heating to 665 K				[56]
Zr_52.5_Cu_17.9_Ni_14.6_Al_10_Ti_5_	Heating to 666 K				[56]
Zr_52.5_Cu_17.9_Ni_14.6_Al_10_Ti_5_	Heating to 670 K			6.35± 0.45	[56]
Zr_52.5_Cu_17.9_Ni_14.6_Al_10_Ti_5_	Heating to 680 K			5.1± 1.7	[56]
Zr_52.5_Cu_17.9_Ni_14.6_Al_10_Ti_5_	Heating to 720 K			3.7± 0.245	[56]
Cu_47.5_Zr_47.5_Al_5_	As‐cast	1680	1980	4.2	[73]
Cu_47.5_Zr_47.5_Al_5_	Relaxed	1617	2186	8.3	[73]
Cu_47.5_Zr_47.5_Al_5_	Rej700 (700 K/min)	1769± 20	1979± 20	4.4± 0.2	[73]
Cu_47.5_Zr_47.5_Al_5_	Rej300 (300 K/min)	1742± 20	1984± 20	4.6± 0.2	[73]
Cu_47.5_Zr_47.5_Al_5_	Rej100 (100 K/min)	1716± 30	2127± 30	9.1± 0.3	[73]
Zr_65_Fe_5_Al_10_Cu_20_	Smooth one	1452± 25	1650± 28	1.9%	[77]
Zr_65_Fe_5_Al_10_Cu_20_	Defected one	1183± 29	1498± 45	2.2%	[77]
Zr_55_Cu_30_Al_10_Ni_5_	As‐cast		∼1737	∼1.5%	[191]
Zr_55_Cu_30_Al_10_Ni_5_	With current		∼1972	∼7.2%	[191]
Fe_39_Ni_39_B_12.82_Si_2.75_Nb_2.3_P_4.13_	As‐cast		3300	∼9.8%	[144]
Fe_39_Ni_39_B_12.82_Si_2.75_Nb_2.3_P_4.13_	Fluxing treatment		4220	>50%	[144]
Zr_46_Cu_37.6_Ag_8.4_Al_8_		1716	1754	0.7	[145]
Zr_53.8_Cu_31.6_Ag_7_Al_7.6_		1518	1816	7.4	[145]
Zr_46.9_Cu_38.4_Ag_8.5_Al_6.2_		1497	1564	1.0	[145]
Zr_43.2_Cu_39.1_Ag_8.7_Al_9_		1690	1715	0.3	[145]
Zr_44.6_Cu_36.5_Ag_8.1_Al_10.8_		1447	1447	0	[145]
Zr_52.5_Ti_5_Cu_18_Ni_14.5_Al_10_	imprinting	1440± 30	1750± 35	0.9± 0.15	[80]
Zr_52.5_Cu_17.9_Ni_14.6_Al_10_Ti_5_	As‐cast	1794± 7		0.14± 0.05	[85]
Zr_52.5_Cu_17.9_Ni_14.6_Al_10_Ti_5_	UVPC‐1	1695± 55		0.76± 0.40	[85]
Zr_52.5_Cu_17.9_Ni_14.6_Al_10_Ti_5_	UVPC‐2	1652± 57		1.47± 0.29	[85]
Zr_52.5_Cu_17.9_Ni_14.6_Al_10_Ti_5_	UVPC‐3	1566± 70		6.29± 1.70	[85]
Zr_64.13_Cu_15.75_Ni_10.12_Al_10_	As‐cast	1613		0	[203]
Zr_64.13_Cu_15.75_Ni_10.12_Al_10_	Pores size – 42 um	1476		0.11	[203]
Zr_64.13_Cu_15.75_Ni_10.12_Al_10_	Pores size – 85 um	1288		0.19	[203]
Zr_64.13_Cu_15.75_Ni_10.12_Al_10_	Pores size – 150 um	1103		0.51	[203]
Zr_64.13_Cu_15.75_Ni_10.12_Al_10_	As‐cast		1614	0	[86]
Zr_64.13_Cu_15.75_Ni_10.12_Al_10_	STS structure 1		1590	0.002	[86]
Zr_64.13_Cu_15.75_Ni_10.12_Al_10_	STS structure 1		1506	0.90	[86]
Zr_55_Cu_30_Al_10_Ni_5_	As‐cast	1778± 17	1789± 13	0.3± 0.1	[200]
Zr_55_Cu_30_Al_10_Ni_5_	Imprinting at 10 kN	1685± 15	1845± 15	2.7± 0.6	[200]
Zr_55_Cu_30_Al_10_Ni_5_	Imprinting at 15 kN	1610± 20	1920± 21	5.9± 0.7	[200]
Zr_55_Cu_30_Al_10_Ni_5_	Imprinting at 20 kN	1590± 25	1910± 20	6.6± 0.8	[200]
Zr_55_Cu_30_Al_10_Ni_5_	Imprinting at 25 kN	1545± 25	1905± 16	7.1± 1.0	[200]
Zr_60_Ni_25_Al_15_	Suction casting	1866	1893	0.5	[205]
Zr_60_Ni_25_Al_15_	Static quenching	1879	2023	6.8	[205]

**FIGURE 28:**
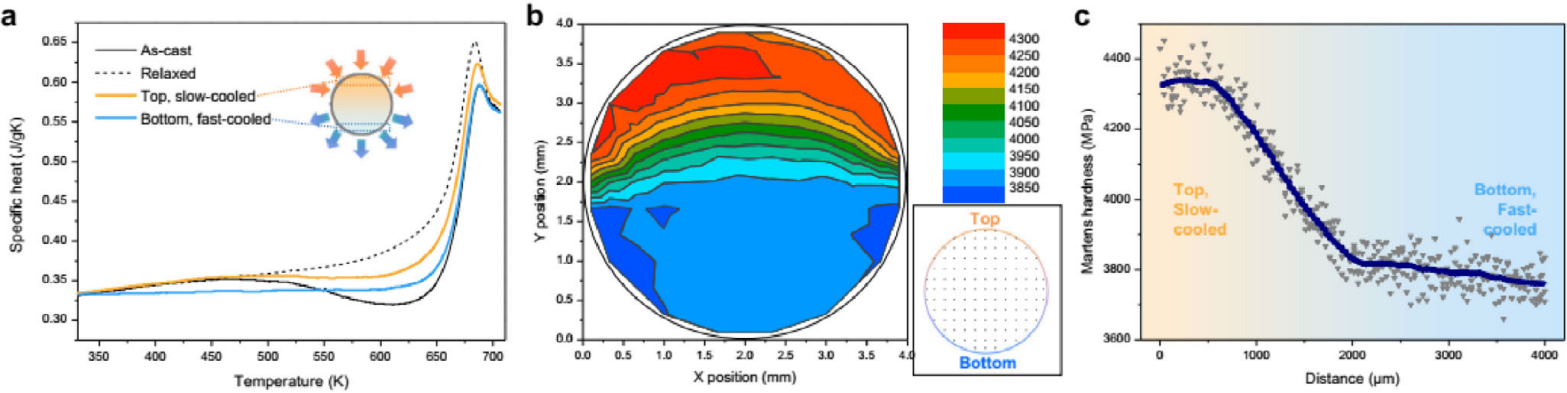
Structures of 2D gradient rejuvenation in a heat‐treated Zr_60_Cu_30_Al_10_ glassy alloys [168]: (a) specific heat curves of glass alloys with various energy states (as‐cast, relaxed, slow‐cooled, and fast‐cooled); (b) 2D nano‐hardness map of the cross‐section of heat processed glassy alloy. The color contour is the value of hardness which has been measured from 16 time from one point and the total measured hardness is from 121 points; (c) hardness‐distance curve of both slow‐cooled and fast‐cooled parts of processed glassy alloy. Reprinted from ref. [168], copyright (2020), with permission from Springer Nature Publishing Group.

The structures of 2D gradient rejuvenation in heat‐treated Zr_60_Cu_30_Al_10_ glassy alloys are shown in Figure 28. Four distinct glassy states – as‐cast, relaxed, slow‐cooled, and fast‐cooled – exhibit different energy levels (relaxation enthalpy) and micro‐hardness, as presented in Figure 28a,b. These variations in physical properties ultimately lead to differences in mechanical performance. Table 5 summarizes various alloy compositions, processing parameters, and the resulting yield strength, fracture strength, and plasticity. Several examples can be highlighted: brittle samples subjected to notching and compression show enhanced plasticity of up to 12% [18]. Amorphous alloys with compositions Zr_44_Ti_11_Cu_9.8_Ni_10.2_Be_2.5_ and Zr_55_Ti_5_Al_10_Cu_20_Ni_10_, processed by cold rolling, display an increase in plastic strain from 0.5% to 15% [122]. Glassy alloys treated with electric current exhibit an improvement in strength from 1.7 to 1.9 GPa, along with enhanced plasticity from 1.5 to 7.2% [191]. BMG with composition Zr_58_Cu_22_Fe_8_Al_12_, after thermal treatment at 711 K, achieves a yield strength of 2.1 GPa and plasticity of 6.25% [8]. Similarly, the glassy alloy Zr_55_Cu_30_Al_10_Ni_5_ shows an increase in plasticity from 6.6% in the as‐cast state to 12.7% after DCT [91]. Although an increasing number of studies have explored influences of processing methods and parameters on mechanical properties, as summarized in Table 5, the fundamental mechanisms responsible for these property enhancements remain unclear. Further investigations are therefore needed.

### 6.2 Work‐Hardening Behaviors

Work‐hardening behavior plays a critical role in determining suitability of amorphous alloys for structural and engineering applications. Structural materials that lack work‐hardening typically exhibit pronounced necking or brittle failure immediately after elastic deformation. In amorphous alloys, the activation of work‐hardening is closely related to the suppression or hindrance of dominant SBs propagation. At the same time, the multiplication and diversification of SBs contribute to enhanced plasticity. In addition, several other hardening mechanisms have been proposed. This section first reviews the work‐hardening mechanisms that have been reported or suggested, and then presents representative examples to demonstrate the relationship between work‐hardening and mechanical properties under both tensile and compressive loading.

When glassy alloys are reduced to the nanometer scale, SBs formation upon deformation is often suppressed. Instead, tensile elongation can exhibit noticeable work‐hardening [206, 207, 208, 209]. Such work‐hardening behavior may be attributed to hinder the nucleation of SBs. In some cases, delayed SBs propagation can also trigger strain hardening, as observed in Cu_49_Zr_51_ melt‐spun alloys under tension [206]. Similarly, electroplated glassy pillars with a composition of Ni_85.1_P_14.9_ display pronounced work‐hardening under tension, which has been attributed to surface aging and the associated reduction in free volume [207]. For many glassy alloy systems subjected to uniaxial compression, strain hardening has been reported to originate from the progressive interactions of SBs [208]. Moreover, when pressure is applied to Zr_50_Cu_44_Al_5_ BMG during uniaxial compression, the resulting hardening is believed to stem from the melting and subsequent re‐solidification of the dominant SBs [209].

For BMG composites, second phases – particularly B2 phases – can induce an apparent activation of strain‐hardening behavior [210, 211, 212, 213]. Two main mechanisms have been proposed. The first involves nanometer‐ or sub‐nanometer‐scale heterogeneities, which play a critical role in formation of a high density of SBs. In this case, strain hardening can be activated through multiple interactions among SBs and the hindrance of their rapid propagation [210]. The second mechanism is transformation‐induced plasticity (TRIP) [211, 212, 213, 214, 215]. Here, the transformation of B2 phases into martensitic crystals under loading, together with the shape change of the remaining B2 phases due to their low shear modulus and subsequent twinning, promotes the activation of work‐hardening. A typical example is the Zr_48_Cu_47.5_Al_4_Co_0.5_ BMG composite, which exhibits a tensile elongation of about 7%. Moreover, phase separation in certain glassy alloys can also play a similar role to that of nanometer‐ or sub‐nanometer‐scale heterogeneities in facilitating strain hardening [148, 216, 217, 218, 219, 220, 221, 222, 223, 224].

Up to now, it has been frequently observed that enhanced plasticity or ductility is usually accompanied by the activation of work hardening [10, 18, 212, 225]. Although there are some exceptions in which extended deformability under uniaxial compression occurs together with strain softening or without any sign of work hardening, such cases will not be discussed here. A few representative examples are as follows. Zr_48_Cu_47.5_Al_4_Co_0.5_ BMG composites containing B2 phase exhibit pronounced work hardening and remarkable tensile ductility [212]. Rejuvenated glassy rods with a composition Zr_64.13_Cu_15.75_Ni_10.12_Al_10_ display clear strain hardening and compressive plasticity up to 12% [18]. Other rejuvenated glassy alloys processed through thermal cycling have also been reported to show both compressive plasticity and activated strain hardening [8, 56, 73, 148, 151, 225]. Moreover, when the characteristic size of glassy alloys is reduced to the nanometer scale, they exhibit tensile elongation of up to 20% along with obvious work‐hardening behavior [206]. Overall, glassy alloys produced through different processing routes inevitably develop distinct microstructures – particularly in terms of atomic cluster configurations – which in turn lead to diverse mechanical properties. It should be emphasized that the direct characterization of atomic clusters remains challenging if one relies solely on conventional trial‐and‐error experiments. In this regard, new approaches, such as the Al method, may prove useful in identifying possible categories of clusters, for example, ductile versus brittle clusters.

### 6.3 Fractured Surface

**FIGURE 29:**
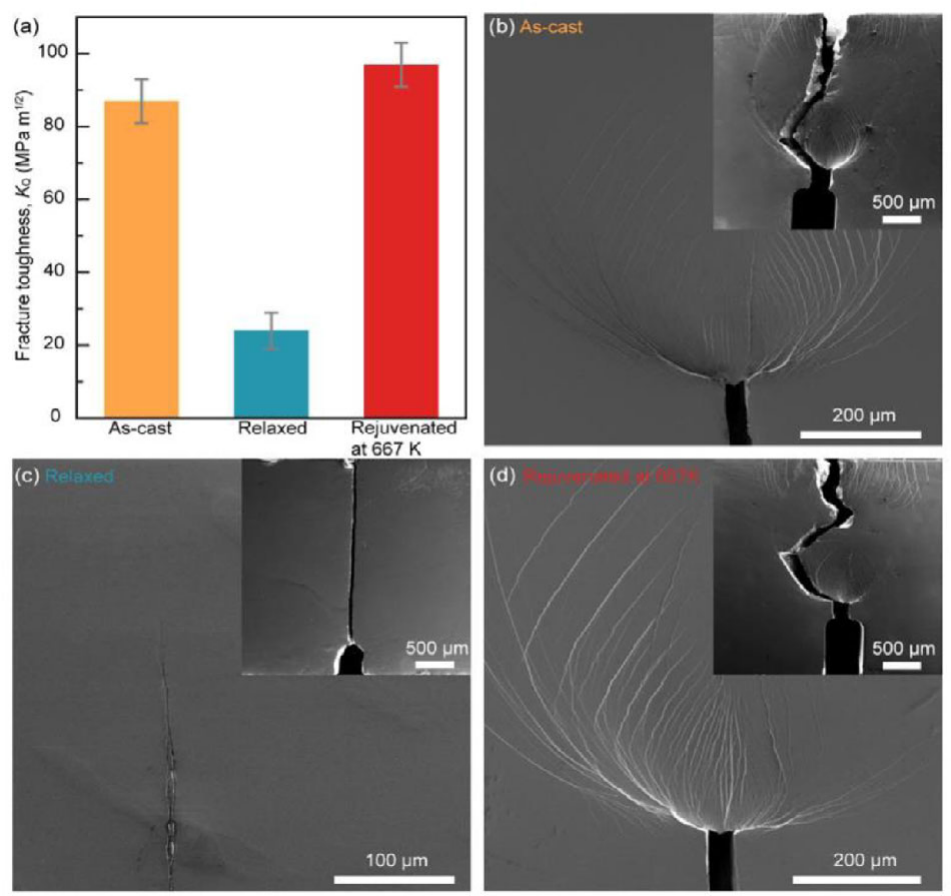
Calculated fracture toughness and SEM images of fractured samples of as‐cast, relaxed and rejuvenated state (Zr_52.5_Cu_17.9_Ni_14.6_Al_10_Ti_5_) [56]: (a) calculated fracture toughness; (b‐d) SEM images of fractured surfaces for three processed alloys. Reprinted from ref. [56], copyright (2022), with permission from Elsevier Ltd Publishing Group.

Many studies have reported that fracture surfaces and modes of brittle glassy alloys differ significantly from those of ductile amorphous alloys. In other words, the underlying microstructures play a decisive role in governing distinct deformation and fracture behaviors. Therefore, analyzing fracture surfaces and modes provides an alternative approach to investigate the evolution of deformation mechanisms and microstructures. In this subsection, a series of fracture morphologies, modes, and rejuvenated glassy alloys will be summarized. To gain a clearer understanding of the correlation between microstructure and fracture, studies on fractured rejuvenated glassy alloys are classified into at least two categories. It should be noted that there is no definite or precise criterion that strictly separates the brittle state from the ductile state in glassy alloys. Accordingly, two representative types of fracture surfaces are illustrated: (i) brittle alloys that fracture prior to yielding, and (ii) ductile alloys that exhibit relatively high plasticity. For glassy alloys, a brittle‐to‐ductile transition is frequently observed when appropriate rejuvenation methods are applied. Such a transition can be regarded as an indicator of the degree of rejuvenation, ranging from partial to full rejuvenation [73] (Figure 29).

**FIGURE 30:**
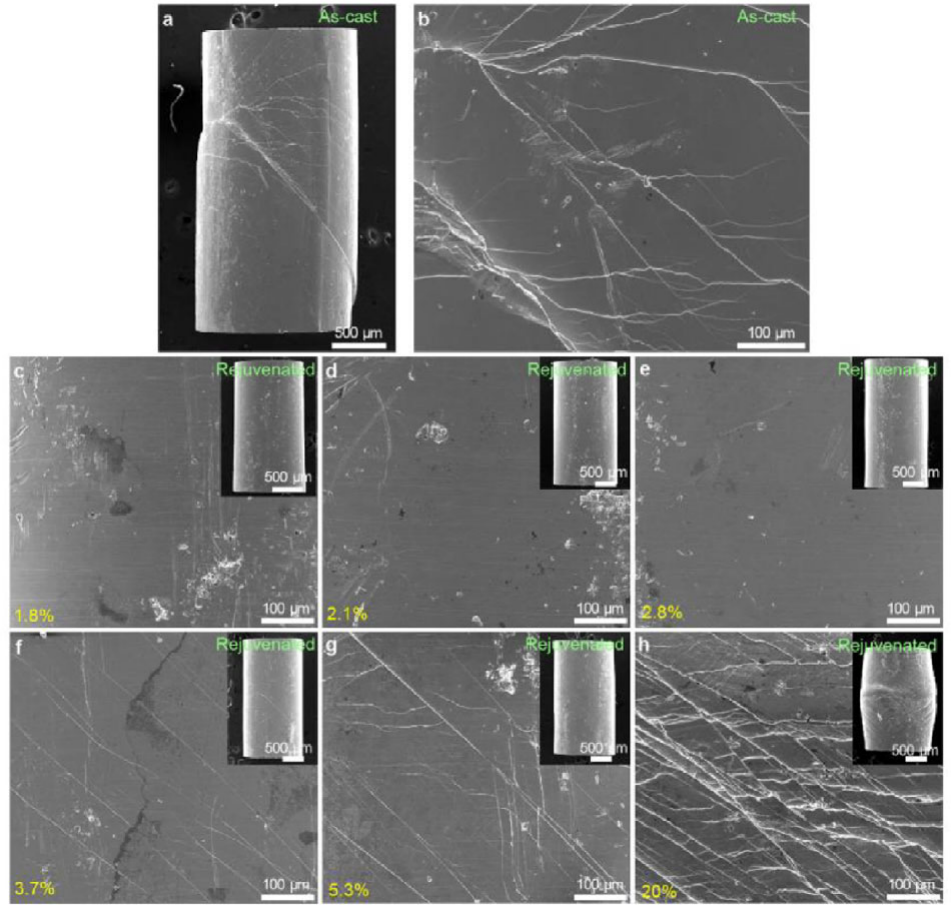
SEM images of comparison between deformation surfaces of as‐cast one and different rejuvenated glassy alloys [18]: (a, b) fractured as‐cast rod and deformed surface; (c–h) different deformation surfaces of various degrees of rejuvenated glassy alloys, insets in (c–h) are fractured or deformed glassy rods. Reprinted from ref. [18], copyright (2020), with permission from Springer Nature Publishing Group.

The transition from brittle to ductile behavior and the corresponding deformed surfaces of glassy alloys are illustrated in Figure 30. As shown, rejuvenated glassy alloys can be transformed into ductile ones, accompanied by a noticeable increase in SBs in terms of spacing, length, and density. Analysis of fractured or deformed surfaces reveals that the distribution and density of SBs are key characteristics of ductile amorphous alloys. In contrast, brittle glassy alloys, as presented in Figure 29b,c, typically exhibit either no SBs or only a single few SBs. Previous studies have also provided evidence that certain processing techniques can be employed to facilitate the deformation transition from brittle to ductile [34, 45, 46, 47, 226].

## 7 Summaries and Unsolved Issues

This review focuses on effects of various rejuvenation methods on microstructures and mechanical properties of glassy alloys, along with associated processing techniques. These rejuvenation approaches play a critical role in tailoring microstructures of glassy alloys, although certain limitations still remain. Therefore, a brief summary of recent findings and discussions is provided.

For three rejuvenation methods – mechanical processing, thermal processing, and other related approaches – a number of brittle glassy alloys have been successfully transformed into ductile ones, with notable improvements in plastic strain and work‐hardening being reported. However, many studies indicate that the processing parameters previously used or reported often need to be re‐evaluated due to mechanical failure. Therefore, systematic summaries of both positive and negative correlations between processing parameters and specific glassy alloys are essential.

The primary goal of these three rejuvenation strategies is to modify microstructures, especially atomic clusters, thereby achieving optimized mechanical properties. According to existing studies, several factors – most importantly the processing parameters and the initial structural states of the glassy alloys – play decisive roles. Future work should focus on consolidating reported findings and exploring variations in both processing parameters and alloy states to achieve more reliable rejuvenation outcomes.

Rejuvenation methods are increasingly regarded as novel processing routes for tailoring microstructures of glassy alloys and their composites. Consequently, the mechanical properties of certain glassy alloys can be substantially enhanced. Nevertheless, despite these advances, many rejuvenated glassy alloys still exhibit brittle behaviors. Since rejuvenation is a relatively new approach designed to mitigate unexpected mechanical failure, a wide ranges of processing factors remain to be investigated. For mechanical rejuvenation, several potential processing concepts can be explored. The SPD, which has long been applied to tune microstructures and mechanical performances of crystalline alloys, may provide valuable insights into parameter selection for glassy alloys. Similarly, the application of artificial intelligence (AI) methods could open new pathways to optimize processing parameters and to better account for the initial states of the glassy alloys. In the case of constrained compressive rejuvenation – such as in notched glassy alloys – enhancing the GFA is a critical prerequisite before scaling up processing tool dimensions. For thermal rejuvenation routes and other processing strategies, the key challenge remains the careful matching of processing parameters with the initial alloy states. At present, such selections must largely rely on published experimental results and AI‐based computations.

The rejuvenated amorphous alloys, precisely, are heterogeneous glassy counterparts, and consist of soft and hard regions macroscopic scale or various types of atomic clusters at microscopic scales. These huge differences in microstructures directly determine the potential energy landscape and therefore decide some features such as physical and mechanical properties. For three general rejuvenation methods – mechanical and thermal rejuvenation and other types of processing rejuvenation routes, they exert similar roles in modifying energy states and microstructures. And macroscopically, the enthalpy and entropy, activation energies as well as calculated flow defects of processed alloys changes with variations of processing parameters and methods. Generally, the higher increase in enthalpy, the higher degree of rejuvenation states, although a few exceptions have been identified [37, 56]. For example, upon LES preloading method [129, 227], some parameters like strain rate and compressive strain indeed influence degree of rejuvenated states. However, the difference in materials like compositions and initial states of alloys, and loading parameters, etc., plays a complex and complicated roles in determining finally rejuvenated states. In this regard, establishing a kinetic criterion that governs the rejuvenation seems to be very difficult. Till now, some pressing issues might involve: (1) identifying relationship between atomic clusters and rejuvenated states; (2) classification of various kinds of atomic clusters corresponding to brittle and ductile features of rejuvenated glassy alloys; (3) precisely control or understanding of developing atomic clusters of rejuvenated glassy alloys. As for relaxation behaviors such as *β*‐relaxations or α‐relaxations, they are still two forms of changed atomic clusters.

Unresolved issues in this field are diverse and complex. Among them, one primary challenge lies in determining the appropriate processing scales for mechanical rejuvenation, particularly in the case of notched samples. While other mechanical approaches, such as SPD, can be applied to bulk glassy alloys, it remains difficult to precisely control the resulting microstructures and to predict their mechanical properties. For instance, glassy alloys subjected to SPD often exhibit reduced plasticity compared with their as‐cast counterparts. In contrast, compressive loading of notched samples has been reported to yield reproducible improvements in mechanical properties; however, this method is restricted to relatively small‐sized specimens. For other mechanical processing techniques, unresolved challenges largely center on optimization of processing parameters, which is inherently complex. The mechanical properties of treated glassy alloys are strongly influenced by two factors: the processing parameters and the initial states of the alloys. Each of these factors encompasses multiple variables, further complicating efforts to achieve consistent and predictable outcomes.

The second issue arises in the thermal rejuvenation method. Thermal rejuvenation processing generally requires: (i) liquid nitrogen or other cryogenic liquids, along with the corresponding processing environment and equipment; and (ii) careful control of the heating rate and processing temperature during the annealing of glassy alloys. The selection of these parameters must also take into account the original states of the glassy alloys. In addition, several critical challenges remain unresolved: (i) the optimal number of processing cycles during DCT; (ii) the appropriate heating rate and annealing temperature; and (iii) the influence of these factors on the rejuvenation effect. The third unresolved issue concerns other types of rejuvenation processing routes. These routes involve a variety of treatments, yet their processing parameters and the resulting states of glassy alloys remain largely unknown. This lack of systematic understanding represents a critical challenge for further advancement.

## Conflicts of Interest

The authors declare no conflicts of interest.

## Data Availability

The data that support the findings of this study are available on request from the corresponding author. The data are not publicly available due to privacy or ethical restrictions.

## References

[1] R. O. Ritchie , “The Conflicts Between Strength and Toughness,” Nature Materials 10, no. 11 (2011): 817–822, 10.1038/nmat3115. 22020005

[2] Y. Sun , A. Concustell , and A. L. Greer , “Thermomechanical Processing of Metallic Glasses: Extending the Range of the Glassy state,” Nature Reviews Materials 1, no. 9 (2016): 16039, 10.1038/natrevmats.2016.39.

[3] H. Lou , Z. Zeng , F. Zhang , et al., “Two‐Way Tuning of Structural Order in Metallic Glasses,” Nature Communications 11 (2020): 314, 10.1038/s41467-019-14129-7. PMC6965127 31949139

[4] E. D. Cubuk , R. J. S. Ivancic , S. S. Schoenholz , et al., “Structure‐Property Relationships From Universal Signatures of Plasticity in Disordered Solids,” Science 358, no. 6366 (2017): 1033–1037, 10.1126/science.aai88. 29170231 PMC6047528

[5] Y. H. Liu , G. Wang , R. J. Wang , D. Q. Zhao , M. X. Pan , and W. H. Wang , “Super Plastic Bulk Metallic Glasses at Room Temperature,” Science 315, no. 5817 (2007): 1385–1388, 10.1126/science.1136726. 17347434

[6] W. Yang , H. Liu , Y. Zhao , et al., “Mechanical Properties and Structural Features of Novel Fe‐Based Bulk Metallic Glasses With Unprecedented Plasticity,” Scientific Report 4 (2014): 6233, 10.1038/srep06233. PMC5385824 25167887

[7] B. Sarac , Y. P. Ivanov , A. Chuvilin , et al., “Origin of Large Plasticity and Multiscale Effects in Iron‐Based Metallic Glasses,” Nature Communications 9, no. 1 (2018): 1333, 10.1038/s41467-018-03744-5. PMC5889395 29626189

[8] Y. Tang , H. Zhou , H. Lu , et al., “Extra Plasticity Governed by Shear Band Deflection in Gradient Metallic Glasses,” Nature Communications 13, no. 1 (2022): 2120, 10.1038/s41467-022-29821-4. PMC9018681 35440578

[9] S. Liu , W. Dong , Z. Ren , et al., “Medium‐Range Order Endows a Bulk Metallic Glass With Enhanced Tensile Ductility,” Journal of Materials Science & Technology 159 (2023): 10–20, 10.1016/j.jmst.2023.02.036.

[10] Y. Chen , C. Tang , and J. Z. Jiang , “Bulk Metallic Glass Composites Containing B2 Phase,” Progress in Materials Science 121 (2021): 100799, 10.1016/j.pmatsci.2021.100799.

[11] M. D. Demetriou , M. E. Launey , G. Garrett , et al., “A Damage‐Tolerant Glass,” Nature Materials 10, no. 2 (2011): 123–128, 10.1038/nmat2930. 21217693

[12] Y. Song , X. Xie , J. Luo , P. K. Liaw , H. Qi , and Y. Gao , “Seeing the Unseen: Uncover the Bulk Heterogeneous Deformation Processes in Metallic Glasses Through Surface Temperature Decoding,” Materials Today 20, no. 1 (2017): 9–15, 10.1016/j.mattod.2016.12.002.

[13] N. V. Priezjev , “Accelerated Rejuvenation in Metallic Glasses Subjected to Elastostatic Compression Along Alternating Directions,” Journal of Non‐Crystalline Solids 556 (2021): 120562, 10.1016/j.jnoncrysol.2020.120562.

[14] L. T. Zhang , Y. J. Wang , E. Pineda , Y. Yang , and J. C. Qiao , “Achieving Structural Rejuvenation in Metallic Glass by Modulating β Relaxation Intensity via Easy‐to‐Operate Mechanical Cycling,” International Journal of Plasticity 157 (2022): 103402, 10.1016/j.ijplas.2022.103402.

[15] Z. Lu , W. H. Wang , and H. Y. Bai , “Classification of Metallic Glasses Based on Structural and Dynamical Heterogeneities by Stress Relaxation,” Science China Materials 58, no. 2 (2015): 98–105, 10.1007/s40843-015-0025-6.

[16] S. Lan , L. Zhu , Z. Wu , et al., “A Medium‐Range Structure Motif Linking Amorphous and Crystalline States,” Nature Materials 20, no. 10 (2021): 1347–1352, 10.1038/s41563-021-01011-5. 34017117

[17] H. W. Sheng , W. K. Luo , F. M. Alamgir , J. M. Bai , and E. Ma , “Atomic Packing and Short‐to‐Medium‐Range Order in Metallic Glasses,” Nature 439, no. 7075 (2006): 419–425, 10.1038/nature04421. 16437105

[18] J. Pan , Y. P. Ivanov , W. H. Zhou , Y. Li , and A. L. Greer , “Strain‐Hardening and Suppression of Shear‐Banding in Rejuvenated Bulk Metallic Glass,” Nature 578, no. 7796 (2020): 559–562, 10.1038/s41586-020-2016-3. 32103194

[19] E. Ma and J. Ding , “Tailoring Structural Inhomogeneities in Metallic Glasses to Enable Tensile Ductility at Room Temperature,” Materials Today 19, no. 10 (2016): 568–579, 10.1016/j.mattod.2016.04.001.

[20] E. Ma , “Tuning Order in Disorder,” Nature Materials 14 (2015): 547–552, 10.1038/nmat4300. 25990900

[21] F. Spaepen , “Metallic Glasses Rejuvenated to Harden Under Strain,” Nature 578, no. 7796 (2020): 521–522, 10.1038/d41586-020-00468-9. 32103189

[22] T. C. Hufnagel , “Cryogenic Rejuvenation,” Nature Materials 14, no. 9 (2015): 867–868, 10.1038/nmat4394. 26288974

[23] S. V. Ketov , Y. H. Sun , S. Nachum , et al., “Rejuvenation of Metallic Glasses by Non‐Affine Thermal Strain,” Nature 524, no. 7564 (2015): 200–203, 10.1038/nature14674. 26268190

[24] P. Kozikowski , M. Ohnuma , R. Hashimoto , et al., “Temperature Memory Effect of Stress Annealing‐Induced Anisotropy in Metallic Glasses,” Physical Review Materials 4, no. 9 (2020): 095604, 10.1103/PhysRevMaterials.4.095604.

[25] M. Tan , Y. Wang , F. Wang , et al., “Rejuvenation Induced Suppression of Crystallization in One La‐Based Metallic Glass by Ultraslow Cold Rolling,” Journal of Non‐Crystalline Solids 636 (2024): 123012, 10.1016/j.jnoncrysol.2024.123012.

[26] F. Meng , K. Tsuchiya , I. I. Seiichiro , and Y. Yokoyama , “Reversible Transition of Deformation Mode by Structural Rejuvenation and Relaxation in Bulk Metallic Glass,” Applied Physics Letters 101, no. 12 (2012): 121914, 10.1063/1.4753998.

[27] H. B. Yu , J. Hu , X. X. Xia , B. Sun , X. X. Li , and H. Bai , “Stress‐Induced Structural Inhomogeneity and Plasticity of Bulk Metallic Glasses,” Scripta Materialia 61 (2009): 640–643, 10.1016/j.scriptamat.2009.06.005.

[28] S. Zhang , B. Shi , J. Wang , and P. Jin , “Stress‐Induced Gradient Rejuvenation Framework and Memory Effect in a Metallic Glass,” Scripta Materialia 213 (2022): 114636, 10.1016/j.scriptamat.2022.114636.

[29] C. Ebner , B. Escher , C. Gammer , J. Eckert , S. Pauly , and C. Rentenberger , “Structural and Mechanical Characterization of Heterogeneities in a CuZr‐Based Bulk Metallic Glass Processed by High Pressure Torsion,” Acta Materialia 160 (2018): 147–157, 10.1016/j.actamat.2018.08.032.

[30] W. Dmowski , Y. Yokoyama , A. Chuang , et al., “Structural Rejuvenation in a Bulk Metallic Glass Induced by Severe Plastic Deformation,” Acta Materialia 58, no. 2 (2010): 429–438, 10.1016/j.actamat.2009.09.021.

[31] Y. Tang , Q. K. Zhao , H. F. Zhou , et al., “Tailoring Microstructure of Metallic Glass for Delocalized Plasticity by Pressure Annealing: Forward and Inverse Studies,” Acta Materialia 220 (2021): 117282, 10.1016/j.actamat.2021.117282.

[32] A. D. Phan , A. Zaccone , V. D. Lam , and K. Wakabayashi , “Theory of Pressure‐Induced Rejuvenation and Strain Hardening in Metallic Glasses,” Physical Review Letters 126, no. 2 (2021): 025502, 10.1103/PhysRevLett.126.025502. 33512192

[33] H. Zhou , R. Hubek , M. Peterlechner , and G. Wilde , “Two‐Stage Rejuvenation and the Correlation Between Rejuvenation Behavior and the Boson Heat Capacity Peak of a Bulk Metallic Glass,” Acta Materialia 179 (2019): 308–316, 10.1016/j.actamat.2019.08.040.

[34] D. Şopu , A. Foroughi , M. Stoica , and J. Eckert , “Brittle‐to‐Ductile Transition in Metallic Glass Nanowires,” Nano Letter 16, no. 7 (2016): 4467–4471, 10.1021/acs.nanolett.6b01636. 27248329

[35] F. Zhu , A. Hirata , P. Liu , et al., “Correlation Between Local Structure Order and Spatial Heterogeneity in a Metallic Glass,” Physical Review Letters 119, no. 21 (2017): 215501, 10.1103/PhysRevLett.119.215501. 29219421

[36] G. Kumar , P. Neibecker , Y. H. Liu , and J. Schroers , “Critical Fictive Temperature for Plasticity in Metallic Glasses,” Nature Communications 4, no. 1 (2013): 1536, 10.1038/ncomms2546. PMC3586724 23443564

[37] S. Y. Zhang , W. H. Zhou , L. J. Song , et al., “Decoupling Between Enthalpy and Mechanical Properties in Rejuvenated Metallic Glass,” Scripta Materialia 223 (2023): 115056, 10.1016/j.scriptamat.2022.115056.

[38] S. Sayad , M. Khanzadeh , G. Alahyarizadeh , and N. Amigo , “A Molecular Dynamics Study on the Mechanical Response of Thermal‐pressure Rejuvenated Cu_x_Zr_100−x_ Metallic Glasses,” Scientific Reports 13, no. 1 (2023): 16109, 10.1038/s41598-023-43432-z. 37752281 PMC10522610

[39] K. Sun , L. M. Xu , H. Weber , et al., “A Refined Local Structure in a Metallic Glass Tailored via Flash‐annealing,” Materials Characterization 178 (2021): 111214, 10.1016/j.matchar.2021.111214.

[40] M. B. Costa , J. J. Londoño , A. Blatter , et al., “Anelastic‐Like Nature of the Rejuvenation of Metallic Glasses by Cryogenic Thermal Cycling,” Acta Materialia 244 (2023): 118551, 10.1016/j.actamat.2022.118551.

[41] X. L. Bian , G. Wang , J. Yi , et al., “Atomic Origin for Rejuvenation of a Zr‐based Metallic Glass at Cryogenic Temperature,” Journal of Alloys and Compounds 718 (2017): 254–259, 10.1016/j.jallcom.2017.05.124.

[42] B. Huang , H. Lv , J. Yi , Q. Wang , and G. Wang , “Size Dependence of Electrical Resistivity Caused by Stress‐Induced Rejuvenation for Metallic Glassy Microfibers,” Materials Research Bulletin 181 (2025): 113112, 10.1016/j.materresbull.2024.113112.

[43] A. Jabed and G. Kumar , “Size‐Effects in Tensile Fracture of Rejuvenated and Annealed Metallic Glass,” Scripta Materialia 241 (2024): 115889, 10.1016/j.scriptamat.2023.115889.

[44] K. Tao , F. Li , Y. Liu , E. Pineda , K. Song , and J. Qiao , “Distinct Avalanche Dynamics Detected in Metallic Glasses With High Energy State Revealing the Crack‐Like Shear Banding Mechanism,” International Journal of Plasticity 174 (2024): 103873, 10.1016/j.ijplas.2023.103873.

[45] L. Wang , Z. Wang , W. Chu , X. Zhao , and L. Hu , “Evolution Path of Metallic Glasses Under Extensive Cryogenic Thermal Cycling: Rejuvenation or Relaxation?,” Materials Science and Engineering: A 850 (2022): 143551, 10.1016/j.msea.2022.143551.

[46] T. J. Lei , L. R. DaCosta , M. Liu , et al., “Microscopic Characterization of Structural Relaxation and Cryogenic Rejuvenation in Metallic Glasses,” Acta Materialia 164 (2019): 165–170, 10.1016/j.actamat.2018.10.036.

[47] M. Wang , S. Lü , S. Wu , and W. Guo , “Rejuvenation Behavior and Microstructural Evolution of Cu‐Zr Metallic Glass During Multiple Recovery Annealing Treatment via Molecular Dynamic Simulation,” Journal of Alloys and Compounds 945 (2023): 169294, 10.1016/j.jallcom.2023.169294.

[48] N. Amigo , “Cryogenic Thermal Cycling Rejuvenation in Metallic Glasses: Structural and Mechanical Assessment,” Journal of Non‐Crystalline Solids 596 (2022): 121850, 10.1016/j.jnoncrysol.2022.121850.

[49] H. Chen , Y. Hai , R. Li , et al., “Defects Controlled Rejuvenation in the Zr_47.5_Cu_47.5_Al_5_ Metallic Glass,” Journal of Alloys and Compounds 927 (2022): 166876, 10.1016/j.jallcom.2022.166876.

[50] Z. Ma , P. Huang , and F. Wang , “Effects of Cryogenic Thermal Cycling on a La‐Based Metallic Glass: Relaxation or Rejuvenation?,” Journal of Alloys and Compounds 909 (2022): 164741, 10.1016/j.jallcom.2022.164741.

[51] S. Di , Q. Wang , Y. Yang , et al., “Efficient Rejuvenation of Heterogeneous {[(Fe_0_._5_Co_0_._5_)_0_._75_B_0_._2_Si_0_._05_]_96_Nb_4_}_99_._9_Cu_0_._1_ Bulk Metallic Glass Upon Cryogenic Cycling Treatment,” Journal of Materials Science & Technology 97 (2022): 20–28, 10.1016/j.jmst.2021.04.034.

[52] S. Küchemann , P. M. Derlet , C. Liu , et al., “Energy Storage in Metallic Glasses via Flash Annealing,” Advanced Functional Materials 28, no. 50 (2018): 1805385, 10.1002/adfm.201805385.

[53] S. Di , Q. Wang , J. Zhou , et al., “Enhancement of Plasticity for FeCoBSiNb Bulk Metallic Glass With Superhigh Strength Through Cryogenic Thermal Cycling,” Scripta Materialia 187 (2020): 13–18, 10.1016/j.scriptamat.2020.05.059.

[54] M. Wang , S. Lü , S. Wu , and W. Guo , “Rejuvenation Behavior of Cu‐Zr‐Al Metallic Glass Under Different Thermal Treatment: Experiments and Simulation,” Journal of Alloys and Compounds 934 (2023): 168058, 10.1016/j.jallcom.2022.168058.

[55] M. Wang , S. Lü , S. Wu , J. Wang , and W. Guo , “Rejuvenation Behavior of Cu–Zr Metallic Glass Under Different Holding Times During Deep Cryogenic Cycling Treatment: Experiments and Simulation,” Journal of Materials Research and Technology 29 (2024): 2750–2757, 10.1016/j.jmrt.2024.02.019.

[56] Y. H. Meng , S. Y. Zhang , W. H. Zhou , et al., “Rejuvenation by Enthalpy Relaxation in Metallic Glasses,” Acta Materialia 241 (2022): 118376, 10.1016/j.actamat.2022.118376.

[57] Y. Yang , J. Geng , Y. Cao , L. Fan , and B. Shi , “Rejuvenation of La‐Based Metallic Glass by Controlling Different Modes of Relaxation,” Scripta Materialia 256 (2025): 116418, 10.1016/j.scriptamat.2024.116418.

[58] X. D. Wang , J. Zhang , T. D. Xu , et al., “Structural Signature of β‐Relaxation in La‐Based Metallic Glasses,” The Journal of Physical Chemistry Letters 9, no. 15 (2018): 4308–4313, 10.1021/acs.jpclett.8b02013. 30016114

[59] L. T. Zhang , Y. J. Wang , Y. Yang , and J. C. Qiao , “Training β Relaxation to Rejuvenate Metallic Glasses,” Journal of Materials Science & Technology 158 (2023): 53–62, 10.1016/j.jmst.2023.02.031.

[60] J. Qiang and K. Tsuchiya , “Composition Dependence of Mechanically‐Induced Structural Rejuvenation in Zr‐Cu‐Al‐Ni Metallic Glasses,” Journal of Alloys and Compounds 712 (2017): 250–255, 10.1016/j.jallcom.2017.04.096.

[61] X. L. Bian , D. Zhao , J. T. Kim , et al., “Controlling the Distribution of Structural Heterogeneities in Severely Deformed Metallic Glass,” Materials Science and Engineering: A 752 (2019): 36–42, 10.1016/j.msea.2019.02.092.

[62] C. Ebner , S. Pauly , J. Eckert , and C. Rentenberger , “Effect of Mechanically Induced Structural Rejuvenation on the Deformation Behaviour of CuZr Based Bulk Metallic Glass,” Materials Science and Engineering: A 773 (2020): 138848, 10.1016/j.msea.2019.138848.

[63] J. Pan , Y. X. Wang , Q. Guo , D. Zhang , A. L. Greer , and Y. Li , “Extreme Rejuvenation and Softening in a Bulk Metallic Glass,” Nature Communications 9, no. 1 (2018): 560, 10.1038/s41467-018-02943-4. PMC5805766 29422622

[64] Y. B. Wang , D. D. Qu , X. H. Wang , et al., “Introducing a Strain‐Hardening Capability to Improve the Ductility of Bulk Metallic Glasses via Severe Plastic Deformation,” Acta Materialia 60, no. 1 (2012): 253–260, 10.1016/j.actamat.2011.09.026.

[65] Q. Hao , G. J. Lyu , E. Pineda , et al., “Deciphering Non‐Elastic Deformation in Amorphous Alloy: Simultaneous Aging‐Induced Ordering and Rejuvenation‐Induced Disordering,” International Journal of Plasticity 175 (2024): 103926, 10.1016/j.ijplas.2024.103926.

[66] Z. Wang , J. Pan , Y. Li , and C. Schuh , “Densification and Strain Hardening of a Metallic Glass Under Tension at Room Temperature,” Physical Review Letters 111 (2013): 135504, 10.1103/PhysRevLett.111.135504. 24116793

[67] Y. Zhu , Y. Zhou , A. Wang , et al., “Atomic‐Scale Icosahedral Short‐Range Ordering in a Rejuvenated Zr‐Based Bulk Metallic Glass Upon Deep Cryogenic Treatment,” Materials Science and Engineering: A 850 (2022): 143565, 10.1016/j.msea.2022.143565.

[68] M. Wakeda , J. Saida , J. Li , and S. Ogata , “Controlled Rejuvenation of Amorphous Metals With Thermal Processing,” Scientific Reports 5 (2015): 10545, 10.1038/srep10545. 26010470 PMC4443766

[69] C. Wang , Q. P. Cao , X. D. Wang , et al., “Intermediate Temperature Brittleness in Metallic Glasses,” Advanced Materials 29, no. 14 (2017): 1605537, 10.1002/adma.201605537. 28181309

[70] S. J. Kang , Q. P. Cao , J. Liu , et al., “Intermediate Structural State for Maximizing the Rejuvenation Effect in Metallic Glass via Thermo‐Cycling Treatment,” Journal of Alloys and Compounds 795 (2019): 493–500, 10.1016/j.jallcom.2019.05.026.

[71] N. V. Priezjev , “The Effect of Cryogenic Thermal Cycling on Aging, Rejuvenation, and Mechanical Properties of Metallic Glasses,” Journal of Non‐Crystalline Solids 503‐504 (2019): 131–138, 10.1016/j.jnoncrysol.2018.09.041.

[72] J. Ketkaew , R. Yamada , H. Wang , et al., “The Effect of Thermal Cycling on the Fracture Toughness of Metallic Glasses,” Acta Materialia 184 (2020): 100–108, 10.1016/j.actamat.2019.11.046.

[73] W. Guo , R. Yamada R , J. Saida , S. Lü , and S. Wu , “Thermal Rejuvenation of a Heterogeneous Metallic Glass,” Journal of Non‐Crystalline Solids 498 (2018): 8–13, 10.1016/j.jnoncrysol.2018.05.038.

[74] Q. Sun , D. M. Miskovic , H. Kong , and M. Ferry , “Transition From Relaxation to Rejuvenation in Ultrastable Metallic Glass Driven by Annealing,” Applied Surface Science 546 (2021): 149048, 10.1016/j.apsusc.2021.149048.

[75] N. Z. Zhang , X. L. Bian , C. Ren , et al., “Manipulation of Relaxation Processes in a Metallic Glass Through Cryogenic Treatment,” Journal of Alloys and Compounds 894 (2022): 162407, 10.1016/j.jallcom.2021.162407.

[76] M. T. Asadi Khanouki , R. Tavakoli , and H. Aashuri , “On the Origin of Intermediate Temperature Brittleness in La‐based Bulk Metallic Glasses,” Journal of Alloys and Compounds 770 (2019): 535–539, 10.1016/j.jallcom.2018.08.131.

[77] R. T. Qu , Q. S. Zhang , and Z. F. Zhang , “Achieving Macroscopic Tensile Plasticity of Monolithic Bulk Metallic Glass by Surface Treatment,” Scripta Materialia 68, no. 11 (2013): 845–848, 10.1016/j.scriptamat.2013.02.005.

[78] Y. B. Ma , B. Z. Wang , Q. D. Zhang , et al., “Change Dynamic Behaviors by Heightening its Stored Energy of Monolithic Bulk Metallic Glass,” Materials & Design 181 (2019): 107971, 10.1016/j.matdes.2019.107971.

[79] C. X. Peng , D. Şopu , Y. Cheng , et al., “Deformation Behavior of Designed Dual‐Phase CuZr Metallic Glasses,” Materials & Design 168 (2019): 107662, 10.1016/j.matdes.2019.107662.

[80] S. Scudino , B. Jerliu , S. Pauly , K. B. Surreddi , U. Kühn , and J. Eckert , “Ductile Bulk Metallic Glasses Produced Through Designed Heterogeneities,” Scripta Materialia 65, no. 9 (2011): 815–818, 10.1016/j.scriptamat.2011.07.039.

[81] D. J. Magagnosc , G. Kumar , J. Schroers , P. Felfer , J. M. Cairney , and D. S. Gianola , “Effect of Ion Irradiation on Tensile Ductility, Strength and Fictive Temperature in Metallic Glass Nanowires,” Acta Materialia 74 (2014): 165–182, 10.1016/j.actamat.2014.04.002.

[82] C. Ebner , J. Rajagopalan , C. Lekka , and C. Rentenberger , “Electron Beam Induced Rejuvenation in a Metallic Glass Film During In‐Situ TEM Tensile Straining,” Acta Materialia 181 (2019): 148–159, 10.1016/j.actamat.2019.09.033.

[83] S. Sohrabi , M. X. Li , H. Y. Bai , J. Ma , W. H. Wang , and A. L. Greer , “Energy Storage Oscillation of Metallic Glass Induced by High‐Intensity Elastic Stimulation,” Applied Physics Letters 116, no. 8 (2020): 081901, 10.1063/1.5140208.

[84] S. Khademorezaian , M. Tomut , M. Peterlechner , et al., “Extreme Rejuvenation of a Bulk Metallic Glass at the Nanoscale by Swift Heavy Ion Irradiation,” Journal of Alloys and Compounds 980 (2024): 173571, 10.1016/j.jallcom.2024.173571.

[85] Y. Lou , X. Liu , X. Yang , et al., “Fast Rejuvenation in Bulk Metallic Glass Induced by Ultrasonic Vibration Precompression,” Intermetallics 118 (2020): 106687, 10.1016/j.intermet.2019.106687.

[86] M. Gao , C. Wang , J. Dong , Y. Huan , H. Y. Bai , and W. H. Wang , “Macroscopic Tensile Plasticity of Zr‐based Bulk Metallic Glass With Surface Screw Thread Shaped Structure,” Materials Science and Engineering: A 673 (2016): 417–422, 10.1016/j.msea.2016.07.024.

[87] C. Yuan , R. Liu , Z. Lv , et al., “Softening in an Ultrasonic‐Vibrated Pd‐Based Metallic Glass,” Intermetallics 144 (2022): 107527, 10.1016/j.intermet.2022.107527.

[88] X. Y. Gong , X. D. Wang , P. Zhang , et al., “Structural Rejuvenation in a Zr‐based Bulk Metallic Glass via Electropulsing Treatment,” Applied Physics Letters 119, no. 4 (2021): 043901, 10.1063/5.0058633.

[89] S. Y. Liang , L. T. Zhang , B. Wang , Y. J. Wang , E. Pineda , and J. C. Qiao , “Structural Rejuvenation and Relaxation of a Metallic Glass Under the Periodically Thermal‐Mechanical Loading,” Intermetallics 164 (2024): 108115, 10.1016/j.intermet.2023.108115.

[90] D. Şopu , F. Spieckermann , X. Bian , S. Fellner , J. Wright , et al., “Rejuvenation Engineering in Metallic Glasses by Complementary Stress and Structure Modulation,” NPG Asia Materials 15, no. 1 (2023): 61, 10.1038/s41427-023-00509-5.

[91] W. Guo , R. Yamada , and J. Saida , “Rejuvenation and Plasticization of Metallic Glass by Deep Cryogenic Cycling Treatment,” Intermetallics 93 (2018): 141–147, 10.1016/j.intermet.2017.11.015.

[92] Z. Chen , S. Ren , R. Zhao , et al., “Plasticity and Rejuvenation of Aged Metallic Glasses by Ultrasonic Vibrations,” Journal of Materials Science & Technology 181 (2024): 231–239, 10.1016/j.jmst.2023.09.029.

[93] S. Su , W. Zhao , X. Su , et al., “Optimizing Structural Ordering Degree to Improve the Mechanical Reliability of Metallic Glasses,” Journal of Materials Science & Technology 227 (2025): 304–314, 10.1016/j.jmst.2024.12.024.

[94] S. Liu , L. Wang , J. Ge , et al., “Deformation‐Enhanced Hierarchical Multiscale Structure Heterogeneity in a Pd‐Si Bulk Metallic Glass,” Acta Materialia 200 (2020): 42–55, 10.1016/j.actamat.2020.08.077.

[95] G. Ding , C. Li , A. Zaccone , et al., “Ultrafast Extreme Rejuvenation of Metallic Glasses by Shock Compression,” Science Advance 5, no. 8 (2019): eaaw6249, 10.1126/sciadv.aaw6249. PMC6707777 31467974

[96] B. Huang , T. P. Ge , G. L. Liu , et al., “Density Fluctuations With Fractal Order in Metallic Glasses Detected by Synchrotron X‐ray Nano‐Computed Tomography,” Acta Materialia 155 (2018): 69–79, 10.1016/j.actamat.2018.05.064.

[97] J. Schroers and W. L. Johnson , “Ductile Bulk Metallic Glass,” Physical Review Letters 93, no. 25 (2004): 255506, 10.1103/PhysRevLett.93.255506. 15697909

[98] C. Liu and R. Maaß , “Elastic Fluctuations and Structural Heterogeneities in Metallic Glasses,” Advanced Functional Materials 28, no. 30 (2018): 1800388, 10.1002/adfm.201800388.

[99] Y. Fan , T. Iwashita , and T. Egami , “Energy Landscape‐Driven Non‐Equilibrium Evolution of Inherent Structure in Disordered Material,” Nature Communications 8, no. 1 (2017): 15417, 10.1038/ncomms15417. PMC5454540 28524879

[100] F. Zhu , H. Nguyen , S. Song , et al., “Intrinsic Correlation Between β‐Relaxation and Spatial Heterogeneity in a Metallic Glass,” Nature Communications 7 (2016): 11516, 10.1038/ncomms11516. PMC4865810 27158084

[101] S. Di , H. Ke , Q. Wang , J. Zhou , Y. Zhao , and B. Shen , “Large Tensile Plasticity Induced by Pronounced β‐Relaxation in Fe‐Based Metallic Glass via Cryogenic Thermal Cycling,” Materials & Design 222 (2022): 111074, 10.1016/j.matdes.2022.111074.

[102] P. Ross , S. Küchemann , P. M. Derlet , et al., “Linking Macroscopic Rejuvenation to Nano‐Elastic Fluctuations in a Metallic Glass,” Acta Materialia 138 (2017): 111–118, 10.1016/j.actamat.2017.07.043.

[103] Y. J. Duan , L. T. Zhang , J. C. Qiao , et al., “Intrinsic Correlation Between the Fraction of Liquidlike Zones and the β Relaxation in High‐Entropy Metallic Glasses,” Physical Review Letters 129, no. 17 (2022): 175501, 10.1103/PhysRevLett.129.175501. 36332263

[104] K. L. Ngai , L. M. Wang , R. Liu , and W. H. Wang , “Microscopic Dynamics Perspective on the Relationship Between Poisson's Ratio and Ductility of Metallic Glasses,” The Journal of Chemical Physics 140, no. 4 (2014): 044511, 10.1063/1.4862822. 25669559

[105] D. P. Wang , Z. G. Zhu , R. Xue , D. W. Ding , H. Bai , and W. H. Wang , “Structural Perspectives on the Elastic and Mechanical Properties of Metallic Glasses,” Journal of Applied Physics 114 (2013): 173505, 10.1063/1.4829028.

[106] Y. Gao , C. Yang , G. Ding , L. H. Dai , and M. Q. Jiang , “Structural Rejuvenation of a Well‐Aged Metallic Glass,” Fundamental Research 4, no. 5 (2024): 1266–1271, 10.1016/j.fmre.2022.12.004. 39431125 PMC11489507

[107] Y. Tong , T. Iwashita , W. Dmowski , H. Bei , Y. Yokoyama , and T. Egami , “Structural Rejuvenation in Bulk Metallic Glasses,” Acta Materialia 86 (2015): 240–246, 10.1016/j.actamat.2014.12.020.

[108] F. Spieckermann , D. Sopu , V. Soprunyuk , et al., “Structure‐Dynamics Relationships in Cryogenically Deformed Bulk Metallic Glass,” Nature Communications 13, no. 1 (2022): 127, 10.1038/s41467-021-27661-2. PMC8748940 35013192

[109] H. B. Yu , X. Shen , Z. Wang , L. Gu , W. H. Wang , and H. Y. Bai , “Tensile Plasticity in Metallic Glasses With Pronounced β Relaxations,” Physical Review Letters 108, no. 1 (2012): 015504, 10.1103/PhysRevLett.108.015504. 22304268

[110] P. Wang and X. Yang , “Atomistic Investigation of Aging and Rejuvenation in CuZr Metallic Glass Under Cyclic Loading,” Computational Materials Science 185 (2020): 109965, 10.1016/j.commatsci.2020.109965.

[111] D. Ouyang , L. Zhao , N. Li , J. Pan , L. Liu , and K. C. Chan , “Atomistic Investigation of Modulating Structural Heterogeneities to Achieve Strength‐ductility Synergy in Metallic Glasses,” Computational Materials Science 217 (2023): 111918, 10.1016/j.commatsci.2022.111918.

[112] B. S. Shang , M. Z. Li , Y. G. Yao , Y. J. Lu , and W. H. Wang , “Evolution of Atomic Rearrangements in Deformation in Metallic Glasses,” Physical Review E 90, no. 4 (2014): 042303, 10.1103/PhysRevE.90.042303. 25375490

[113] S. D. Feng , K. C. Chan , L. Zhao , et al., “Rejuvenation by Weakening the Medium Range Order in Zr_46_Cu_46_Al_8_ Metallic Glass With Pressure Preloading: A Molecular Dynamics Simulation Study,” Materials & Design 158 (2018): 248–255, 10.1016/j.matdes.2018.08.040.

[114] S. Li , P. Huang , and F. Wang , “Rejuvenation Saturation Upon Cyclic Elastic Loading in Metallic Glass,” Computational Materials Science 166 (2019): 318–325, 10.1016/j.commatsci.2019.05.007.

[115] I. Lobzenko , Y. Shiihara , T. Iwashita , and T. Egami , “Shear Softening in a Metallic Glass: First‐Principles Local‐Stress Analysis,” Physical Review Letters 124, no. 8 (2020): 085503, 10.1103/PhysRevLett.124.085503. 32167329

[116] J. Yu , M. Wang , and S. Lin , “Slower Icosahedral Cluster Rejuvenation Drives the Brittle‐to‐Ductile Transition in Nanoscale Metallic Glasses,” Computational Materials Science 140 (2017): 235–243, 10.1016/j.commatsci.2017.08.038.

[117] L. Zhao , K. C. Chan , S. H. Chen , S. D. Feng , D. X. Han , and G. Wang , “Tunable Tensile Ductility of Metallic Glasses With Partially Rejuvenated Amorphous Structures,” Acta Materialia 169 (2019): 122–134, 10.1016/j.actamat.2019.03.007.

[118] L. M. Ruschel , S. Jakovlev , O. Gross , et al., “Unraveling the Role of Relaxation and Rejuvenation on the Structure and Deformation Behavior of the Zr‐based Bulk Metallic Glass Vit105,” Materials Today Advances 23 (2024): 100522, 10.1016/j.mtadv.2024.100522.

[119] F. Meng , K. Tsuchiya , M. J. Kramer , and R. T. Ott , “Reduction of Shear Localization Through Structural Rejuvenation in Zr–Cu–Al Bulk Metallic Glass,” Materials Science and Engineering: A 765 (2019): 138304, 10.1016/j.msea.2019.138304.

[120] P. Denis , C. M. Meylan , C. Ebner , A. L. Greer , M. Zehetbauer , and H. J. Fecht , “Rejuvenation Decreases Shear Band Sliding Velocity in Pt‐based Metallic Glasses,” Materials Science and Engineering: A 684 (2017): 517–523, 10.1016/j.msea.2016.12.075.

[121] L. Zhang , Y. Wu , S. Feng , et al., “Rejuvenated Metallic Glass Strips Produced via Twin‐Roll Casting,” Journal of Materials Science & Technology 38 (2020): 73–79, 10.1016/j.jmst.2019.08.022.

[122] M. H. Lee , K. S. Lee , J. Das , J. Thomas , U. Kühn , and J. Eckert , “Improved Plasticity of Bulk Metallic Glasses Upon Cold Rolling,” Scripta Materialia 62, no. 9 (2010): 678–681, 10.1016/j.scriptamat.2010.01.024.

[123] B. Li , K. Nomoto , S. Xie , S. P. Ringer , B. Gludovatz , and J. J. Kruzic , “Controlling the Relaxation Versus Rejuvenation Behavior in Zr‐Based Bulk Metallic Glasses Induced by Elastostatic Compression,” Materials Science and Engineering: A 855 (2022): 143906, 10.1016/j.msea.2022.143906.

[124] K. W. Park , C. M. Lee , M. Wakeda , Y. Shibutani , M. L. Falk , and J. C. Lee , “Elastostatically Induced Structural Disordering in Amorphous Alloys,” Acta Materialia 56, no. 19 (2008): 5440–5450, 10.1016/j.actamat.2008.07.033.

[125] M. B. Costa and A. L. Greer , “Enthalpy of Anelasticity and Rejuvenation of Metallic Glasses,” Acta Materialia 265 (2024): 119609, 10.1016/j.actamat.2023.119609.

[126] M. Samavatian , R. Gholamipour , A. A. Amadeh , and S. Mirdamadi , “Extra Rejuvenation of Zr_55_Cu_30_Al_10_Ni_5_ Bulk Metallic Glass Using Elastostatic Loading and Cryothermal Treatment Interaction,” Journal of Non‐Crystalline Solids 506 (2019): 39–45, 10.1016/j.jnoncrysol.2018.12.007.

[127] M. Samavatian , R. Gholamipour , A. A. Amadeh , and V. Samavatian , “Inherent Relation Between Atomic‐level Stresses and Nanoscale Heterogeneity in Zr‐Based Bulk Metallic Glass Under a Rejuvenation Process,” Physica B: Condensed Matter 595 (2020): 412390, 10.1016/j.physb.2020.412390.

[128] M. Zhang , Y. M. Wang , F. X. Li , S. Q. Jiang , M. Z. Li , and L. Liu , “Mechanical Relaxation‐to‐Rejuvenation Transition in a Zr‐Based Bulk Metallic Glass,” Scientific Reports 7, no. 1 (2017): 625, 10.1038/s41598-017-00768-7. 28377604 PMC5429611

[129] S. Zhang , B. Shi , J. Wang , Y. Xu , and P. Jin , “Rejuvenation of a Naturally Aged Bulk Metallic Glass by Elastostatic Loading,” Materials Science and Engineering: A 806 (2021): 140843, 10.1016/j.msea.2021.140843.

[130] S. Sohrabi , B. Y. Sun , M. Mahmoodan , Y. H. Sun , R. Gholamipour , and W. H. Wang , “Rejuvenation by Compressive Elasto‐Static Loading: The Role of Static Stress on a Zr‐Based Metallic Glass,” Journal of Alloys and Compounds 933 (2023): 167715, 10.1016/j.jallcom.2022.167715.

[131] K. W. Shao , W. H. Zhou , K. Gao , X. G. Zhu , P. Jia , and Y. Li , “Rejuvenation by Triaxial Compression in a Brittle La‐Based Bulk Metallic Glass,” Materials Letters 320 (2022): 132336, 10.1016/j.matlet.2022.132336.

[132] A. H. Balal , X. L. Bian , D. X. Han , et al., “Long‐Term Elasto‐Static Compressive Loading Drives Rejuvenation of a Metallic Glass,” Materials Characterization 212 (2024): 113977, 10.1016/j.matchar.2024.113977.

[133] W. Lu , B. Huang , S. Liao , et al., “Structural Heterogeneity and Plasticity of a Zr‐Based Metallic Glass Modulated by High‐Temperature Deformation,” Journal of Applied Physics 135, no. 19 (2024): 195102, 10.1063/5.0204346.

[134] N. Ren , R. Wan , T. Meng , et al., “Understanding the Atomic‐Scale Effects of Cyclic Strains on Metallic Glasses,” Journal of Non‐Crystalline Solids 652 (2025): 123424, 10.1016/j.jnoncrysol.2025.123424.

[135] S. Y. Liang , L. T. Zhang , Y. J. Wang , B. Wang , J. M. Pelletier , and J. C. Qiao , “A Model on the Coupling Between Cyclic Fatigue and Microstructure Evolution in a Metallic Glass,” International Journal of Fatigue 187 (2024): 108446, 10.1016/j.ijfatigue.2024.108446.

[136] C. Yang , H. B. Zhou , J. Duan , et al., “Evaluating Plasticity of Rejuvenated Metallic Glasses by Effective Enthalpy,” Fundamental Research (2025), 10.1016/j.fmre.2025.03.008.

[137] C. M. Meylan , J. Orava , and A. L. Greer , “Rejuvenation Through Plastic Deformation of a La‐Based Metallic Glass Measured by Fast‐Scanning Calorimetry,” Journal of Non‐Crystal Solids X 8 (2020): 100051, 10.1016/j.nocx.2020.100051.

[138] S. M. Mirhashemi and M. Malekan , “Elastostatic Loading Rejuvenation Behavior in Bulk Metallic Glasses and its Origin: A Review,” Journal of Materials Research and Technology 35 (2025): 3349–3370, 10.1016/j.jmrt.2025.02.033.

[139] A. Foroughi , H. Ashuri , R. Tavakoli , M. Stoica , D. Şopu , and J. Eckert , “Structural Modification Through Pressurized Sub‐Tg Annealing of Metallic Glasses,” Journal of Applied Physics 122, no. 21 (2017): 215106, 10.1063/1.5004058.

[140] R. Yamada , Y. Shibazaki , Y. Abe , W. Ryu , and J. Saida , “Unveiling a New Type of Ultradense Anomalous Metallic Glass With Improved Strength and Ductility Through a High‐Pressure Heat Treatment,” NPG Asia Materials 11, no. 1 (2019): 72, 10.1038/s41427-019-0175-1.

[141] R. M. O. Mota , E. T. Lund , S. Sohn , et al., “Enhancing Ductility in Bulk Metallic Glasses by Straining During Cooling,” Communications Materials 2, no. 1 (2021): 23, 10.1038/s43246-021-00127-0.

[142] X. X. Li , J. G. Wang , H. B. Ke , C. Yang , and W. H. Wang , “Extreme Rejuvenation and Superior Stability in a Metallic Glass,” Materials Today Physics 27 (2022): 100782, 10.1016/j.mtphys.2022.100782.

[143] S. Li , Y. Yu , P. S. Branicio , and Z. D. Sha , “Effects of Rejuvenation Modes on the Microstructures and Mechanical Properties of Metallic Glasses,” Materials Today Communications 36 (2023): 106493, 10.1016/j.mtcomm.2023.106493.

[144] J. Zhou , Q. Wang , Q. Zeng , et al., “A Plastic FeNi‐Based Bulk Metallic Glass and its Deformation Behavior,” Journal of Materials Science & Technology 76 (2021): 20–32, 10.1016/j.jmst.2020.11.016.

[145] X. Wang , Q. P. Cao , Y. M. Chen , et al., “A Plastic Zr–Cu–Ag–Al Bulk Metallic Glass,” Acta Materialia 59, no. 3 (2011): 1037–1047, 10.1016/j.actamat.2010.10.034.

[146] C. C. Yuan , Z. W. Lv , C. M. Pang , et al., “Atomic‐Scale Heterogeneity in Large‐Plasticity Cu‐Doped Metallic Glasses,” Journal of Alloys and Compounds 798 (2019): 517–522, 10.1016/j.jallcom.2019.05.282.

[147] Y. Tang , H. F. Zhou , X. D. Wang , Q. P. Cao , D. X. Zhang , and J. Z. Jiang , “Origin of Different Thermal Cycling Effects in Fe_80_P_20_ and Ni_60_Nb_40_ Metallic Glasses,” Materials Today Physics 17 (2021): 100349, 10.1016/j.mtphys.2021.100349.

[148] Y. Tang , H. B. Xiao , X. D. Wang , Q. P. Cao , D. X. Zhang , and J. Z. Jiang , “Mechanical Property and Structural Changes by Thermal Cycling in Phase‐Separated Metallic Glasses,” Journal of Materials Science & Technology 78 (2021): 144–154, 10.1016/j.jmst.2020.10.050.

[149] M. Bruns , M. Hassani , F. Varnik , A. Hassanpour , S. Divinski , and G. Wilde , “Decelerated Aging in Metallic Glasses by Low Temperature Thermal Cycling,” Physical Review Research 3, no. 1 (2021): 013234, 10.1103/PhysRevResearch.3.013234.

[150] P. Gong , G. Yin , Z. Jamili‐Shirvan , H. Ding , X. Wang , and J. Jin , “Influence of Deep Cryogenic Cycling on the Rejuvenation and Plasticization of TiZrHfBeCu High‐Entropy Bulk Metallic Glass,” Materials Science and Engineering: A 797 (2020): 140078, 10.1016/j.msea.2020.140078.

[151] L. L. Wang , Y. C. Li , F. L. Shi , Z. Wang , and L. N. Hu , “An Effective Criterion for Predicting Rejuvenation Capacity of Metallic Glasses: Quantification of Relaxation‐Mode Competitions,” Acta Materialia 303 (2026): 121702, 10.1016/j.actamat.2025.121702.

[152] Q. Dong , C. J. Li , B. Sarac , J. Eckert , and J. Tan , “Enhancing Plasticity of Metallic Glasses via Rejuvenation: A Review,” Transactions of Nonferrous Metals Society of China 35 (2025): 3961–3984, 10.1016/S1003-6326(25)66924-X.

[153] H. M. Guan and M. C. Li , “Enhancement of Plasticity by Cryogenic Thermal Cycling on Fe_80_P_13_C_7_ Bulk Amorphous Alloy,” Materials Letters 300 (2021): 130195, 10.1016/j.matlet.2021.130195.

[154] J. C. Qiao , Y. J. Wang , L. Z. Zhao , et al., “Transition From Stress‐Driven to Thermally Activated Stress Relaxation in Metallic Glasses,” Physical Review B 94, no. 10 (2016): 104203, 10.1103/PhysRevB.94.104203.

[155] W. Guo , S. Yu , J. Ding , S. L. LÜ , S. S. Wu , and M. Zhao , “Tailoring Rejuvenation Behavior of Zr‐Based Metallic Glass Upon Deep Cryogenic Cycling Treatment,” Transactions of Nonferrous Metals Society of China 34, no. 2 (2024): 582–591, 10.1016/S1003-6326(23)66419-2.

[156] S. Zhang , X. Wang , J. Hay , U. D. Schwarz , and A. Datye , “Thermal Cycling‐Induced Evolution of Structure and Local Mechanical Properties in Metallic Glass,” Journal of Alloys and Compounds 994 (2024): 174709, 10.1016/j.jallcom.2024.174709.

[157] N. Zhang , F. Spieckermann , X. Yuan , et al., “Unusual Hardness and String‐Like Structures Relaxation of Metallic Glass Investigated by In‐Situ Synchrotron Radiation,” Journal of Alloys and Compounds 1010 (2025): 178287, 10.1016/j.jallcom.2024.178287.

[158] T. Yan , L. Zhang , Y. Wu , et al., “Non‐Monotonic Influence of Cryogenic Thermal Cycling on Rejuvenation and Impact Toughness of Ti‐Based Bulk Metallic Glass Composites,” Scripta Materialia 228 (2023): 115340, 10.1016/j.scriptamat.2023.115340.

[159] B. Huang , H. Bai , and W. H. Wang , “Relationship Between Boson Heat Capacity Peaks and Evolution of Heterogeneous Structure in Metallic Glasses,” Journal of Applied Physics 115 (2014): 153505, 10.1063/1.4871676.

[160] W. J. Sun , Y. Q. Wang , J. D. Zuo , J. Y. Zhang , G. Liu , and J. Sun , “Thermal Annealing Affected Microstructure Evolution and Creep Behavior in Amorphous TaTiZr Medium‐Entropy Alloy,” Journal of Materials Science & Technology 225 (2025): 174–187, 10.1016/j.jmst.2024.11.034.

[161] W. J. Sun , T. Q. Li , Y. Q. Wang , et al., “Thermally‐Driven Structural Inhomogeneity and Serrated Plastic Flow in TaTiZr Amorphous Medium‐Entropy Alloy,” Acta Materialia 286 (2025): 120764, 10.1016/j.actamat.2025.120764.

[162] K. K. Qiu , X. D. Wang , T. D. Xu , et al., “Two‐Step Annealing Induced Structural Rejuvenation: A Cause for Memory Effect in Metallic Glasses,” Materials Today Physics 27 (2022): 100824, 10.1016/j.mtphys.2022.100824.

[163] L. T. Zhang , Y. J. Wang , Y. Yang , and J. C. Qiao , “Aging and Rejuvenation During High‐Temperature Deformation in a Metallic Glass,” Science China Physics, Mechanics & Astronomy 65 (2022): 106111, 10.1007/s11433-022-1953-x.

[164] J. Tan , Y. Zhang , B. Sun , et al., “Correlation Between Internal States and Plasticity in Bulk Metallic Glass,” Applied Physics Letters 98 (2011): 151906, 10.1063/1.3580774.

[165] L. Wang , Z. Wang , and L. Hu , “Rejuvenation to Relaxation Transition and Liquid Memory Effect in La‐Based Metallic Glasses With Different Energy States,” Intermetallics 156 (2023): 107864, 10.1016/j.intermet.2023.107864.

[166] S. Yuan , A. Liang , C. Liu , L. Tian , N. Mousseau , and P. S. Branicio , “Effect of Heat Treatment Paths on the Aging and Rejuvenation of Metallic Glasses,” Physical Review Materials 7, no. 12 (2023): 123603, 10.1103/PhysRevMaterials.7.123603.

[167] K. Sun , X. D. Yuan , N. Z. Zhang , et al., “Non‐Monotonic Fluctuation of Structural Heterogeneity in Metallic Glass Due to Cyclic Rapid Heat Treatment,” Applied Physics Letters 126, no. 2 (2025): 021901, 10.1063/5.0245102.

[168] W. Ryu , R. Yamada , and J. Saida , “Tailored Hardening of ZrCuAl Bulk Metallic Glass Induced by 2D Gradient Rejuvenation,” NPG Asia Materials 12 (2020): 52, 10.1038/s41427-020-0233-8.

[169] A. Tabassum , T. Bashir , Y. Liu , et al., “Tailoring the Pressure Effects to Optimize the Global Structural Features in Ni_80_P_20_ Metallic Glasses,” Solid State Communications 398 (2025): 115872, 10.1016/j.ssc.2025.115872.

[170] Z. Chang , G. Yao , Y. Ge , and X. Yue , “Tunable Rejuvenation Behavior of Zr_55_Cu_35_Al_10_ Metallic Glass at the Atomic Scale During Recovery Annealing and Pressure Treatment,” Materials Today Communications 41 (2024): 110646, 10.1016/j.mtcomm.2024.110646.

[171] T. P. Ge , C. Wang , J. Tan , et al., “Unusual Energy State Evolution in Ce‐Based Metallic Glass Under High Pressure,” Journal of Applied Physics 121, no. 20 (2017): 205109, 10.1063/1.4983017.

[172] W. Guo , Y. Shao , M. Zhao , S. Lü , and S. Wu , “Varying the Treating Conditions to Rejuvenate Metallic Glass by Deep Cryogenic Cycling Treatment,” Journal of Alloys and Compounds 819 (2020): 152997, 10.1016/j.jallcom.2019.152997.

[173] A. L. Greer and Y. H. Sun , “Stored Energy in Metallic Glasses due to Strains Within the Elastic Limit,” Philosophical Magazine 96, no. 16 (2016): 1643–1663, 10.1080/14786435.2016.1177231.

[174] Y. Tong , W. Dmowski , H. Bei , Y. Yokoyama , and T. Egami , “Mechanical Rejuvenation in Bulk Metallic Glass Induced by Thermo‐Mechanical Creep,” Acta Materialia 148 (2018): 384–390, 10.1016/j.actamat.2018.02.019.

[175] W. Guo , Y. Shao , J. Saida , M. Zhao , S. Lü , and S. Wu , “Rejuvenation and Plasticization of Zr‐Based Bulk Metallic Glass With Various Ta Content Upon Deep Cryogenic Cycling,” Journal of Alloys and Compounds 795 (2019): 314–318, 10.1016/j.jallcom.2019.04.340.

[176] J. Z. Wang , W. H. Zhou , Z. P. Luo , et al., “Repeated Rejuvenation From Relaxed Metallic Glasses Through the Memory Effect,” Acta Materialia 303 (2026): 121732, 10.1016/j.actamat.2025.121732.

[177] S. Y. Liang , L. T. Zhang , B. Wang , et al., “Decoupling Thermal and Mechanical Effects on Metallic Glasses Creep,” International Journal of Mechanical Sciences 302 (2025): 110573, 10.1016/j.ijmecsci.2025.110573.

[178] M. Wang , S. Lü , S. Wu , X. Chen , and W. Guo , “Rejuvenation Behaviors of Recovery‐Annealed Cu–Zr Metallic Glass With Different Thermal Treatment Conditions: A Molecular Dynamics Study,” Journal of Materials Research and Technology 20 (2022): 3355–3362, 10.1016/j.jmrt.2022.08.083.

[179] W. Song , X. Meng , Y. Wu , et al., “Improving Plasticity of the Zr_46_Cu_46_Al_8_ Bulk Metallic Glass via Thermal Rejuvenation,” Science Bulletin 63, no. 13 (2018): 840–844, 10.1016/j.scib.2018.04.021. 36658963

[180] X. Li , Y. Hong , H. Ke , L. Zhong , Y. Zou , and J. Wang , “In Situ TEM Study of Pulse‐Enhanced Plasticity of Monatomic Metallic Glasses,” Journal of Materials Science & Technology 195 (2024): 208–217, 10.1016/j.jmst.2023.12.068.

[181] Z. Y. Zhou , H. L. Peng , and H. B. Yu , “Structural Origin for Vibration‐Induced Accelerated Aging and Rejuvenation in Metallic Glasses,” The Journal of Chemical Physics 150, no. 20 (2019): 204507, 10.1063/1.5094825. 31153173

[182] Q. Wang , Y. Yang , H. Jiang , C. Liu , H. H. Ruan , and J. Lu , “Superior Tensile Ductility in Bulk Metallic Glass With Gradient Amorphous Structure,” Scientific Reports 4 (2014): 4757, 10.1038/srep04757. 24755683 PMC3996486

[183] C. C. Yuan , Z. W. Lv , X. Li , et al., “Ultrasonic‐Promoted Defect Activation and Structural Rejuvenation in a La‐Based Metallic Glass,” Intermetallics 153 (2023): 107803, 10.1016/j.intermet.2022.107803.

[184] W. Li , C. Wang , L. Y. Li , et al., “Manipulating Defects in Metallic Glasses via Ultrasonic Treatment,” International Journal of Mechanical Sciences 287 (2025): 109960, 10.1016/j.ijmecsci.2025.109960.

[185] H. J. Cai , M. Zhang , J. R. Shi , et al., “Enhancing Rejuvenation of Metallic Glass via Vibration‐Superimposed Elastic Loads,” International Journal of Mechanical Sciences 295 (2025): 110285, 10.1016/j.ijmecsci.2025.110285.

[186] Y. Li , Y. Wei , K. Zhang , et al., “Rejuvenation, Embryonic Shear Bands and Improved Tensile Plasticity of Metallic Glasses by Nanosecond Laser Shock Wave,” Journal of Non‐Crystalline Solids 513 (2019): 76–83, 10.1016/j.jnoncrysol.2019.02.031.

[187] J. Dong , M. Gao , Y. Huan , Y. H. Feng , W. Liu , and W. H. Wang , “Enhanced Tensile Plasticity of Zr Based Bulk Metallic Glasses by a Stress Induced Large Scale Flow,” Journal of Alloys and Compounds 727 (2017): 297–303, 10.1016/j.jallcom.2017.08.046.

[188] Z. H. Mahmoud , H. Barazandeh , S. M. Mostafavi , et al., “Identification of Rejuvenation and Relaxation Regions in a Zr‐Based Metallic Glass Induced by Laser Shock Peening,” Journal of Materials Research and Technology 11 (2021): 2015–2020, 10.1016/j.jmrt.2021.02.025.

[189] C. Yang , J. Duan , G. Ding , et al., “Splitting of Fast Relaxation in a Metallic Glass by Laser Shocks,” Physical Review B 109, no. 2 (2024): 024201, 10.1103/PhysRevB.109.024201.

[190] Y. Cheng , S. Pang , C. Chen , and T. Zhang , “Tailoring Residual Stress to Achieve Large Plasticity in Zr_55_Al_10_Ni_5_Cu_30_ Bulk Metallic Glass,” Journal of Alloys and Compounds 690 (2017): 176–181, 10.1016/j.jallcom.2016.08.075.

[191] H. Ding , P. Gong , W. Chen , et al., “Achieving Strength‐Ductility Synergy in Metallic Glasses via Electric Current‐Enhanced Structural Fluctuations,” International Journal of Plasticity 169 (2023): 103711, 10.1016/j.ijplas.2023.103711.

[192] Q. Chen , M. Zhang , J. Gu , et al., “Expanding the Homogeneous Regime of Deformation in Bulk Metallic Glass by Electromigration‐Induced Rejuvenation,” Communications Materials 1, no. 1 (2020): 44, 10.1038/s43246-020-0046-x.

[193] G. Liu , Y. Zhou , W. Chen , G. Xing , J. Qiao , and T. Wang , “Self‐Thermal Cycling Rejuvenation of Zr‐Based Bulk Metallic Glasses by the Treatment of Loading Direct Current,” Intermetallics 178 (2025): 108622, 10.1016/j.intermet.2024.108622.

[194] K. Sun , G. Wang , Y. W. Wang , et al., “Structural Rejuvenation and Relaxation of a Metallic Glass Induced by Ion Irradiation,” Scripta Materialia 180 (2020): 34–39, 10.1016/j.scriptamat.2020.01.023.

[195] Y. Zhang , W. H. Wang , and A. L. Greer , “Making Metallic Glasses Plastic by Control of Residual Stress,” Nature Materials 5 (2006): 857–860, 10.1038/nmat1758. 17041581

[196] A. Concustell , F. O. Méar , S. Suriñach , M. D. Baró , and A. L. Greer , “Structural Relaxation and Rejuvenation in a Metallic Glass Induced by Shot‐Peening,” Philosophical Magazine Letters 89, no. 12 (2009): 831–840, 10.1080/09500830903337919.

[197] L. Deng , K. Kosiba , R. Limbach , L. Wondraczek , U. Kühn , and S. Pauly , “Plastic Deformation of a Zr‐Based Bulk Metallic Glass Fabricated by Selective Laser Melting,” Journal of Materials Science & Technology 60 (2021): 139–146, 10.1016/j.jmst.2020.06.007.

[198] S. Scudino , B. Jerliu , K. B. Surreddi , U. Kühn , and J. Eckert , “Effect of Cold Rolling on Compressive and Tensile Mechanical Properties of Zr_52.5_Ti_5_Cu_18_Ni_14.5_Al_10_ Bulk Metallic Glass,” Journal of Alloys and Compounds 509 (2011): S128–S130, 10.1016/j.jallcom.2011.01.022.

[199] S. Scudino , H. S. Shahabi , M. Stoica , et al., “Structural Features of Plastic Deformation in Bulk Metallic Glasses,” Applied Physics Letters 106, no. 3 (2015): 031903, 10.1063/1.4906305.

[200] L. Zhao , D. Han , S. Guan , X. Lu , K. Chan , and G. Wang , “Simultaneous Improvement of Plasticity and Strength of Metallic Glasses by Tailoring Residual Stress: Role of Stress Gradient on Shear Banding,” Materials & Design 197 (2021): 109246, 10.1016/j.matdes.2020.109246.

[201] S. Y. Kim , E. S. Park , R. T. Ott , et al., “Imprinting Bulk Amorphous Alloy at Room Temperature,” Scientific Reports 5 (2015): 16540, 10.1038/srep16540. 26563908 PMC4643295

[202] S. Scudino , J. J. Bian , H. Shakur Shahabi , et al., “Ductile Bulk Metallic Glass by Controlling Structural Heterogeneities,” Scientific Reports 8, no. 1 (2018): 9174, 10.1038/s41598-018-27285-5. 29907778 PMC6003957

[203] M. Gao , J. Dong , Y. Huan , Y. Wang , and W. H. Wang , “Macroscopic Tensile Plasticity by Scalarizating Stress Distribution in Bulk Metallic Glass,” Scientific Reports 6 (2016): 21929, 10.1038/srep21929. 26902264 PMC4763289

[204] K. B. Kim , X. F. Zhang , S. Yi , M. H. Lee , J. Das , and J. Eckert , “Effect of Local Chemistry, Structure and Length Scale of Heterogeneities on the Mechanical Properties of a Ti_45_Cu_40_Ni_7_._5_Zr_5_Sn_2_._5_ Bulk Metallic Glass,” Philosophical Magazine Letters 88, no. 1 (2008): 75–81, 10.1080/09500830701736338.

[205] Q. Hou , T. Wang , J. Zhou , X. Zhou , Q. Hao , and J. Qiao , “The Improvement of the Plasticity of a Zr—Ni—Al Bulk Metallic Glass by Static Quenching,” Materials Science and Engineering: A 851 (2022): 143624, 10.1016/j.msea.2022.143624.

[206] L. Tian , Y. Q. Cheng , Z. W. Shan , et al., “Approaching the Ideal Elastic Limit of Metallic Glasses,” Nature Communications 3, no. 1 (2012): 609, 10.1038/ncomms1619. PMC3272572 22215084

[207] D. Z. Chen , X. W. Gu , Q. An , W. A. Goddard III , and J. R. Greer , “Ductility and Work Hardening in Nano‐Sized Metallic Glasses,” Applied Physics Letters 106, no. 6 (2015): 061903, 10.1063/1.4907773.

[208] R. T. Qu , Z. Q. Liu , G. Wang , and Z. F. Zhang , “Progressive Shear Band Propagation in Metallic Glasses Under Compression,” Acta Materialia 91 (2015): 19–33, 10.1016/j.actamat.2015.03.026.

[209] J. G. Wang , Y. C. Hu , P. F. Guan , et al., “Hardening of Shear Band in Metallic Glass,” Scientific Reports 7, no. 1 (2017): 7076, 10.1038/s41598-017-07669-9. 28765652 PMC5539228

[210] J. Das , M. B. Tang , K. B. Kim , et al., ““Work‐Hardenable” Ductile Bulk Metallic Glass,” Physical Review Letters 94, no. 20 (2005): 205501, 10.1103/PhysRevLett.94.205501. 16090260

[211] S. Pauly , S. Gorantla , G. Wang , U. Kühn , and J. Eckert , “Transformation‐Mediated Ductility in CuZr‐Based Bulk Metallic Glasses,” Nature Materials 9, no. 6 (2010): 473–477, 10.1038/nmat2767. 20473286

[212] Y. Wu , Y. Xiao , G. Chen , C. T. Liu , and Z. Lu , “Bulk Metallic Glass Composites With Transformation‐Mediated Work‐Hardening and Ductility,” Advanced Materials 22, no. 25 (2010): 2770–2773, 10.1002/adma.201000482. 20422654

[213] Y. Chen , C. Tang , K. Laws , Q. Zhu , and M. Ferry , “Zr‐Co‐Al Bulk Metallic Glass Composites Containing B2 ZrCo via Rapid Quenching and Annealing,” Journal of Alloys and Compounds 820 (2020): 153079, 10.1016/j.jallcom.2019.153079.

[214] K. K. Song , S. Pauly , Y. Zhang , et al., “Triple Yielding and Deformation Mechanisms in Metastable Cu_47.5_Zr_47.5_Al_5_ Composites,” Acta Materialia 60, no. 17 (2012): 6000–6012, 10.1016/j.actamat.2012.07.015.

[215] C. P. Kim , Y. S. Oh , S. Lee , and N. J. Kim , “Realization of High Tensile Ductility in a Bulk Metallic Glass Composite by the Utilization of Deformation‐Induced Martensitic Transformation,” Scripta Materialia 65, no. 4 (2011): 304–307, 10.1016/j.scriptamat.2011.04.037.

[216] R. Rashidi , M. Malekan , and R. Gholamipour , “Microstructure and Mechanical Properties of a Cu‐Zr Based Bulk Metallic Glass Containing Atomic Scale Chemical Heterogeneities,” Materials Science and Engineering: A 729 (2018): 433–438, 10.1016/j.msea.2018.05.082.

[217] K. F. Yao and C. Q. Zhang , “Fe‐Based Bulk Metallic Glass With High Plasticity,” Applied Physics Letters 90, no. 6 (2007): 061901, 10.1063/1.2437722.

[218] S. Xie , X. Tu , and J. J. Kruzic , “Inducing Strain Hardening in a Zr‐Based Bulk Metallic Glass via Cobalt Mediated Phase Separations,” Journal of Alloys and Compounds 735 (2018): 1576–1581, 10.1016/j.jallcom.2017.11.223.

[219] T. Wang , J. Si , Y. Wu , K. Lv , Y. Liu , and X. Hui , “Two‐Step Work‐Hardening and its Gigantic Toughening Effect in Zr‐Based Bulk Metallic Glasses,” Scripta Materialia 150 (2018): 106–109, 10.1016/j.scriptamat.2018.03.006.

[220] B. J. Park , H. J. Chang , D. H. Kim , et al., “Phase Separating Bulk Metallic Glass: A Hierarchical Composite,” Physical Review Letters 96, no. 24 (2006): 245503, 10.1103/PhysRevLett.96.245503. 16907253

[221] E. S. Park , J. S. Kyeong , and D. H. Kim , “Phase Separation and Improved Plasticity by Modulated Heterogeneity in Cu–(Zr,Hf)–(Gd,Y)–Al Metallic Glasses,” Scripta Materialia 57, no. 1 (2007): 49–52, 10.1016/j.scriptamat.2007.03.008.

[222] S. Chen , J. Tu , J. Wu , et al., “Phase Separation and Significant Plastic Strain in a Zr–Cu–Ni–Al–Fe Bulk Metallic Glass,” Materials Science and Engineering: A 656 (2016): 84–89, 10.1016/j.msea.2016.01.032.

[223] X. H. Du , J. C. Huang , H. M. Chen , et al., “Phase‐Separated Microstructures and Shear‐Banding Behavior in a Designed Zr‐Based Glass‐Forming Alloy,” Intermetallics 17, no. 8 (2009): 607–613, 10.1016/j.intermet.2009.01.019.

[224] S. S. Chen , H. R. Zhang , and I. Todd , “Phase‐Separation‐Enhanced Plasticity in a Cu_47_._2_Zr_46_._5_Al_5_._5_Nb_0_._8_ Bulk Metallic Glass,” Scripta Materialia 72‐73 (2014): 47–50, 10.1016/j.scriptamat.2013.10.011.

[225] D. Wang , M. Du , Y. Lin , et al., “Hierarchical Micro‐Nanostructured Zr‐Based Metallic Glass With Tensile Plasticity,” Acta Materialia 283 (2025): 120553, 10.1016/j.actamat.2024.120553.

[226] X. Yuan , D. Şopu , F. Moitzi , K. K. Song , and J. Eckert , “Intrinsic and Extrinsic Effects on the Brittle‐to‐Ductile Transition in Metallic Glasses,” Journal of Applied Physics 128, no. 12 (2020): 125102, 10.1063/5.0020201.

[227] L. T. Zhang , Y. J. Duan , Y. J. Wang , et al., “Creep and Recovery Behavior of Metallic Glasses in a Global Strain Approach Within Transition State Theory,” Acta Mechanica Sinica 42 (2026): 425311, 10.1007/s10409-025-25311-x.

